# Robustness of AI Models to Synthetic Data in Color Image Classification: A Comparative Analysis

**DOI:** 10.3390/jimaging12100502

**Published:** 2026-10-10

**Authors:** Marina Barulina, Sergey Okunkov, Ivan Ulitin

**Affiliations:** 1 Institute of Physical and Mathematical Sciences, Perm State University, 614068 Perm, Russia; sergeiokunkov@yandex.ru (S.O.); ivulitin@psu.ru (I.U.); 2 Institute of Precision Mechanics and Control, Russian Academy of Sciences, 410028 Saratov, Russia

**Keywords:** deep learning, class imbalance, augmentation, synthetic data, multiclass classification, model performance improvement, state-of-the-art architectures, vision transformer

## Abstract

This study analyzed how the performance of various neural network architectures responds to offline image augmentation using standard geometric and photometric transformations. We evaluated a composite training-data enrichment protocol using two multiclass color image classification tasks: a dataset of leg photographs classified according to the CEAP system (sourced from open-source repositories; 20,971 images, seven classes) and a dermatological image dataset (6786 images, eight classes). To enrich the initially balanced subsets, transformed images were added to classes underrepresented in the original datasets, while additional real images were added to the remaining classes. Twelve architectures were investigated, including classical CNNs, vision transformers, and a modernized CNN. Across the 12 architectures, 11 augmentation levels, and 20 runs per configuration, mean accuracy ranged from 0.55 to 0.68 (leg photographs) and from 0.59 to 0.79 (dermatological images). The expansion levels yielding the highest observed mean accuracy varied across configurations, with descriptive maxima occurring at levels from 0% to 100%. For each model–dataset configuration, the highest mean accuracy across the eleven levels was treated as a descriptive maximum. Standard deviations across runs characterized the variability of the training process, whereas standard errors reflected the uncertainty of mean estimates given a fixed test set. Transformer-based configurations demonstrated lower average run-to-run standard deviations; however, this tendency was configuration- and dataset-dependent, warranting further research. An exploratory augmentation-as-biased-variance-reduction (ABVR) model is proposed to describe the observed accuracy curves. Its fitted coefficients and maximizers summarize the available data and have not been independently validated.

## 1. Introduction

High-quality data are the foundation of any effective artificial intelligence system. The performance of such systems depends heavily on the characteristics of the dataset used, including sample size, data balance, example diversity, number of classes, presence of bias, and the overall representativeness of the data within the studied domain. Consequently, poor model performance in machine learning and deep learning tasks often stems from insufficient or low-quality source data.

To address these limitations, data augmentation techniques are frequently applied to generate synthetic data, offering a cost-effective way to expand datasets and improve model generalization. The underlying assumption is that label-preserving perturbations of the input expand the effective coverage of the data distribution and thus provide an additional learning signal [1,2].

Data augmentation has attracted sustained research interest. However, most research in this area focuses on studying the impact of synthetic data on specific architectures and tasks, and rarely addresses the design questions that arise when planning experiments with synthetic data. The common finding of such work is that moderate use of augmentation slightly improves prediction quality.

Yang et al. [1] evaluate augmentation across image classification, semantic segmentation and object detection. Their results include both improvements and small deteriorations, indicating that the effect depends on the task, model and transformation.

Alomar et al. [3] review augmentation methods for classification and segmentation and propose a random local rotation strategy, which outperformed conventional rotation in their reported comparisons.

Shorten and Khoshgoftaar [2] review conventional and more complex image-augmentation approaches. The cited gains of approximately 3–3.5% refer to particular CIFAR-10 experiments and do not establish superiority over results obtained on other datasets or with other baselines.

Article [4] discusses an approach to increasing the sample size using synthetic data in a highly specialized field (medicine), where some image augmentation methods are completely inapplicable.

Mariani et al. [5] propose BAGAN to augment underrepresented classes and evaluate downstream classification using ResNet-18 on MNIST, CIFAR-10, Flowers and GTSRB. The relative benefits vary across datasets and augmentation methods.

Shorten et al. [6] review symbolic and neural methods for text augmentation, including rule-based transformations, back-translation and generative approaches. Recent work addresses LLM-based synthetic-data generation [7,8], adaptation to low-resource languages [9], and augmentation for named entity recognition [10].

The authors of article [11] focus on the results of applying augmentation when training classical machine learning models (SVM, KNN, RF, DT). They show that synthetic data can have a very positive effect when training models that are not related to neural networks. The reported gain exceeded 3.5%. Note that gains from different studies are not directly comparable because they were obtained on different datasets, tasks and baselines.

However, despite its popularity and demonstrated benefits, data augmentation is not a universal solution. Suitable algorithms are not available for all data types or tasks, and in some cases, the generation of synthetic examples can distort key features or alter target labels, leading to degraded model performance. The present study compared the responses of CNN, transformer and modernized-CNN configurations to a graded, class-selective enrichment protocol combining transformed and additional real images. Its focus was the observed response of these configurations, rather than an isolated causal effect of synthetic data or a controlled test of class-imbalance correction.

Several practical questions arise when applying augmentation techniques:

1. What is the optimal percentage of synthetic data beyond which performance gains cease or begin to decline?
2. How do synthetic data affect different neural network architectures?
3. How do class-wise metrics respond to enrichment of classes that were underrepresented in the original dataset?
4. How do different augmentation strategies influence model prediction quality?
5. Can this approach be reliably applied to specific types of tasks?

This study addressed three research questions:

RQ1: How do observed accuracy curves and their maxima vary across the evaluated model–dataset configurations?
RQ2: How does run-to-run variability differ between these configurations?
RQ3: How do class-wise metrics change for classes that were underrepresented in the original datasets?

RQ3 is descriptive because the baseline pools are balanced and enrichment changes several components of the training data simultaneously. Our contributions are a common evaluation protocol with 11 enrichment levels, 12 architectures and 20 runs per configuration on two datasets, a comparison of the resulting performance and variability, and an exploratory ABVR response model fitted to the measured curves. Section 2 describes the datasets, models, protocol and modeling assumptions, Section 3 reports the results, Section 4 discusses their interpretation and limitations, and Section 5 concludes.

## 2. Materials and Methods

### 2.1. Datasets

Two datasets were used: the primary CEAP dataset [12,13] and a dermatological replication dataset [14] from a different domain. Models were trained independently for each dataset. In the implementation focused on skin lesion analysis, the evaluation subsample was used both for checkpoint selection and for the subsequent calculation of metrics, rather than as a hold-out test set. Thus, the process involved replicating the experimental methodology rather than externally validating a model trained on the primary dataset. The term “internal validation subsample” is reserved for the subset of data used during the training process.

**Figure 1:**
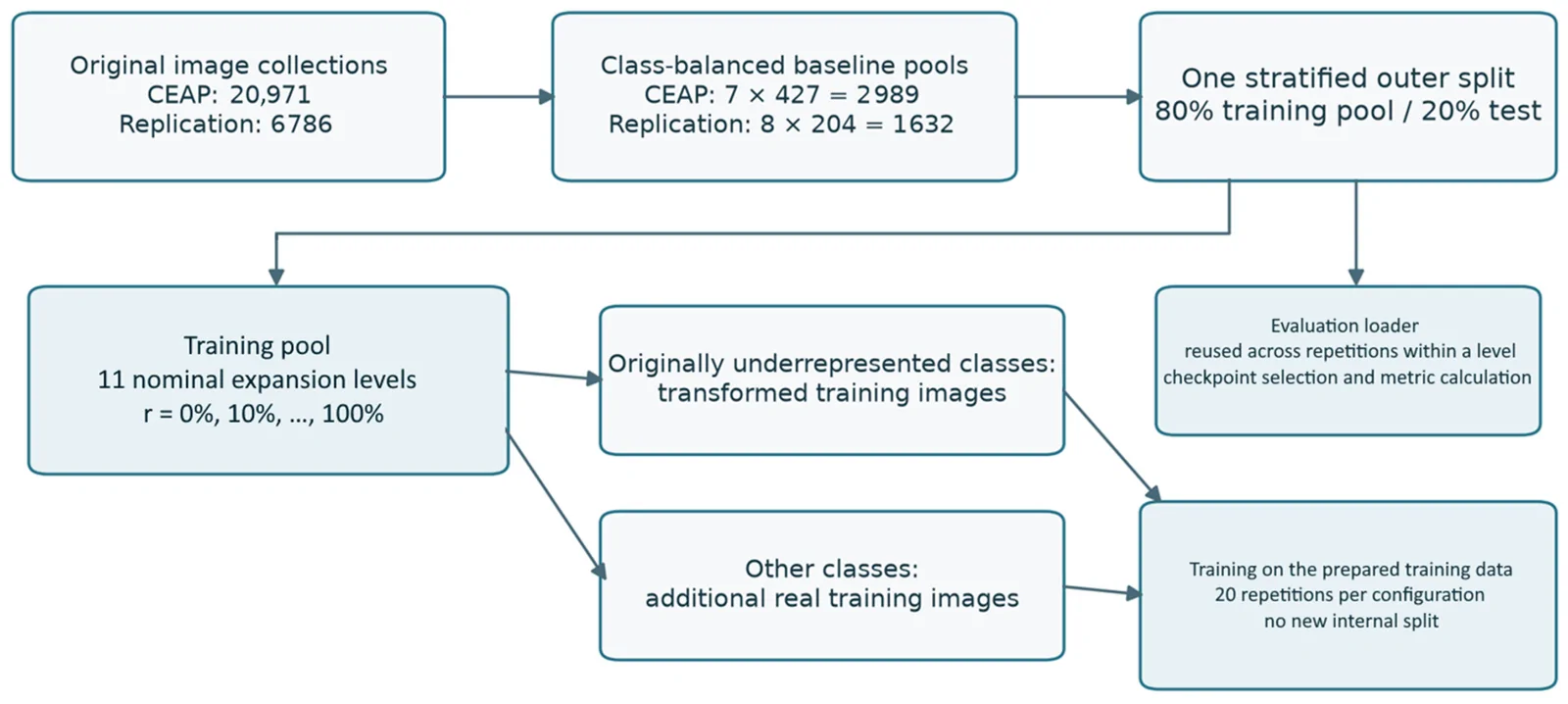
Construction of the experimental datasets: undersampling to a balanced pool, stratified training–testing partitioning, and graded expansion of the training pool with transformed images from originally underrepresented classes and additional real images from the remaining classes. Percentages denote nominal expansion relative to the baseline training-pool size, not the transformed-image fraction. The held-out test part remains fixed.

Both datasets were processed using the following procedure (Figure 1).

1. Initial data: The initial samples were highly unbalanced sets of images divided into several classes.
2. Data balancing: each dataset was undersampled to the size of its smallest class (427 images per class for the main dataset, i.e., 2989 of 20,971 images, 14.3%; 204 images per class for the second dataset, i.e., 1632 of 6786 images, 24.0%). After undersampling, all classes in a dataset contain the same number of images. The terms “originally underrepresented” and “originally frequent” refer only to their frequencies before undersampling. Let *N*_0_ be the baseline training-pool size, *N_s_* the number of added transformed images and *Nᵣ* the number of added real images at a given level, all counted within the constructed training pool. The nominal expansion level is *r* = (*N_s_* + *N_r_*)/*N*_0_, whereas the actual transformed-image fraction is *f_s_* = *N_s_*/(*N*_0_ + *N_s_* + *N_r_*). A nominal level of 100% denotes doubling the baseline size, subject to integer rounding, not a fully synthetic dataset. In this mixed-addition protocol, *f_s_* ≤ *r*/(1 + *r*), with equality only if *Nᵣ* = 0.
3. Sample division: The balanced dataset was loaded from pre-prepared train–test directories, with an 80%–20% split of samples. The same test set, consisting of real images, was used for all data augmentation levels and all 20 experimental runs. Augmented derivatives of the training images were not included in the test set. These precautions prevent any direct overlap between the original data and their derivatives, as required by the protocol. The reported results do not evaluate model performance under patient-level, duplicate-cluster-level, or source-level (website-level) data splits.
4. The process of constructing training sets involved incrementally increasing the size of the training portion in 10% steps relative to the initial size (+239 images per step for the main dataset), resulting in 11 expansion levels ranging from 0% to 100%. For classes initially lacking sufficient data, transformed images derived from the original training images were added, whereas other classes incorporated previously unused real images from the original set. Each training set generated in this manner was saved separately. Consequently, the level metric characterizes a comprehensive data enrichment procedure that alters the quantity and type of training images (and potentially the class distribution), rather than merely reflecting the proportion of synthetic images within the combined dataset. The 0% expansion condition provides a real-only baseline. Comparisons with this baseline evaluate changes associated with the enrichment protocol, but do not isolate the contribution of transformed images from accompanying changes in training-set size, real-image additions or class composition.

**Figure 2:**
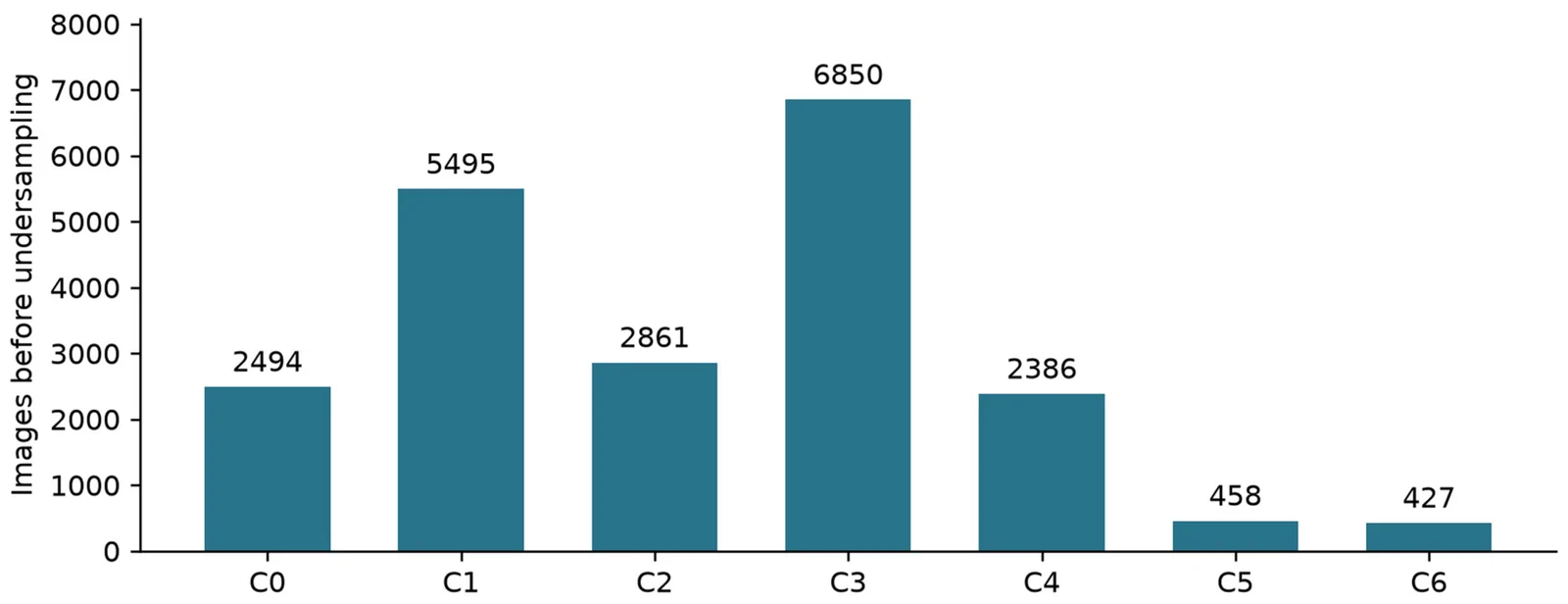
Main-dataset image counts by CEAP class before undersampling, reproduced from Table 1.

**Figure 3:**
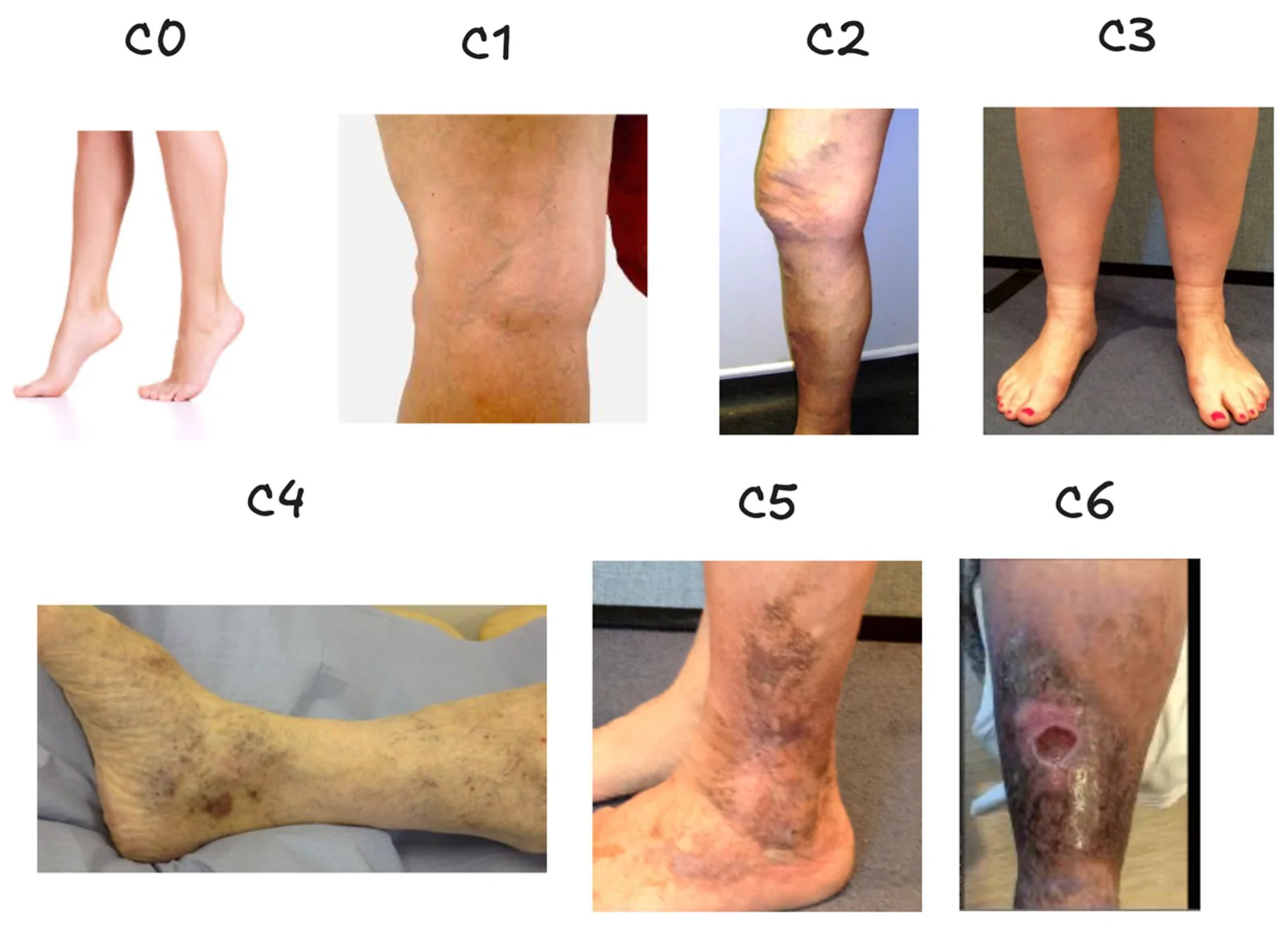
Representative images from each class of the main dataset. (**C0**) no visible signs; (**C1**) telangiectasia or reticular veins; (**C2**) varicose veins; (**C3**) edema; (**C4**) skin changes; (**C5**) healed ulcer; (**C6**) active ulcer.

The main dataset comprised 20,971 RGB leg photographs assigned to the seven clinical CEAP classes C0–C6 [12,13,15]: C0, no visible signs; C1, telangiectasia or reticular veins; C2, varicose veins; C3, edema; C4, skin changes; C5, healed ulcer; and C6, active ulcer. The current repository description identifies the labels as assigned by a certified phlebologist [13]. The earlier dataset-development study [12] describes web collection using Scrapy for Instagram material and Selenium for Google image searches, manual removal of non-leg and low-quality images, a ResNet50-based “legs–no legs” filter, and CEAP categorization by a certified phlebologist. The present experiments use the 20,971-image release and the class counts in Table 1 and in Figure 2. The class distribution in the present release was C0 (11.89%), C1 (26.2%), C2 (13.64%), C3 (32.66%), C4 (11.38%), C5 (2.18%) and C6 (2.04%). Examples are shown in Figure 3. The published dataset-development report documents expert CEAP categorization. The clinical-class labels should not be interpreted as a complete patient-level CEAP assessment including etiological, anatomical and pathophysiological information.

**Figure 4:**
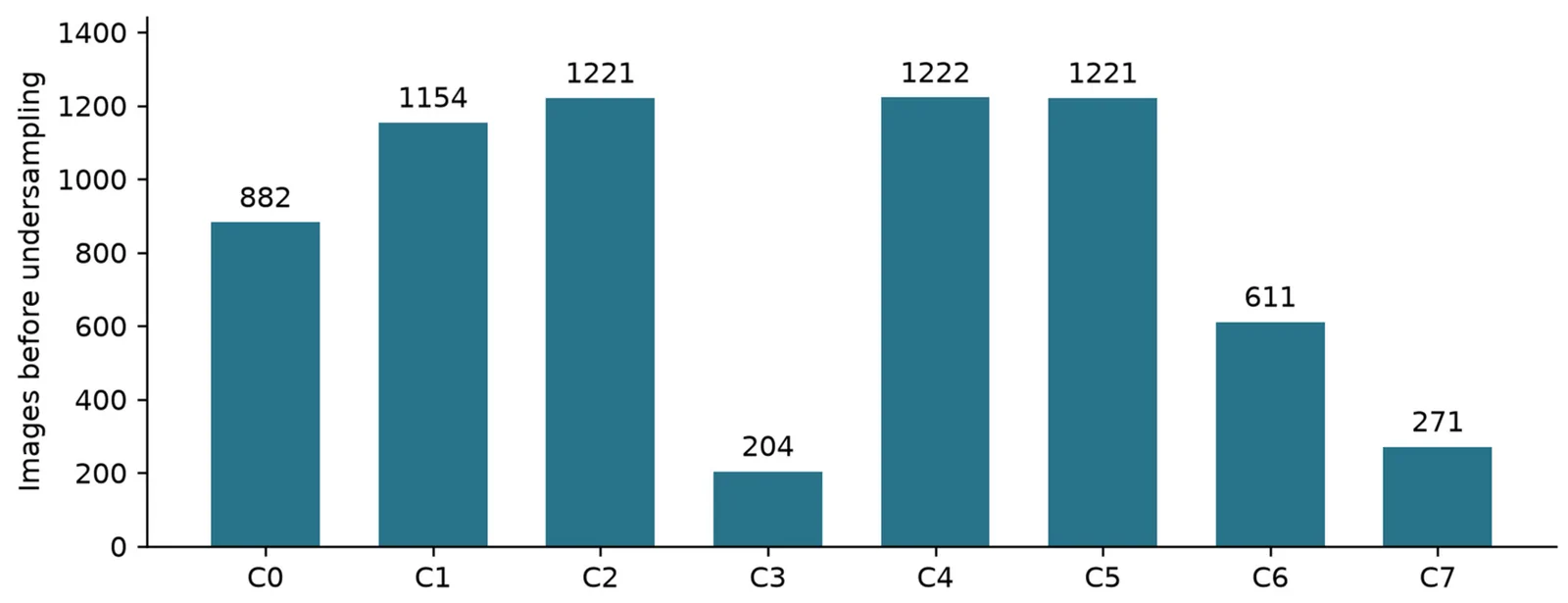
Replication-dataset image counts by class before undersampling, reproduced from Table 2.

**Figure 5:**
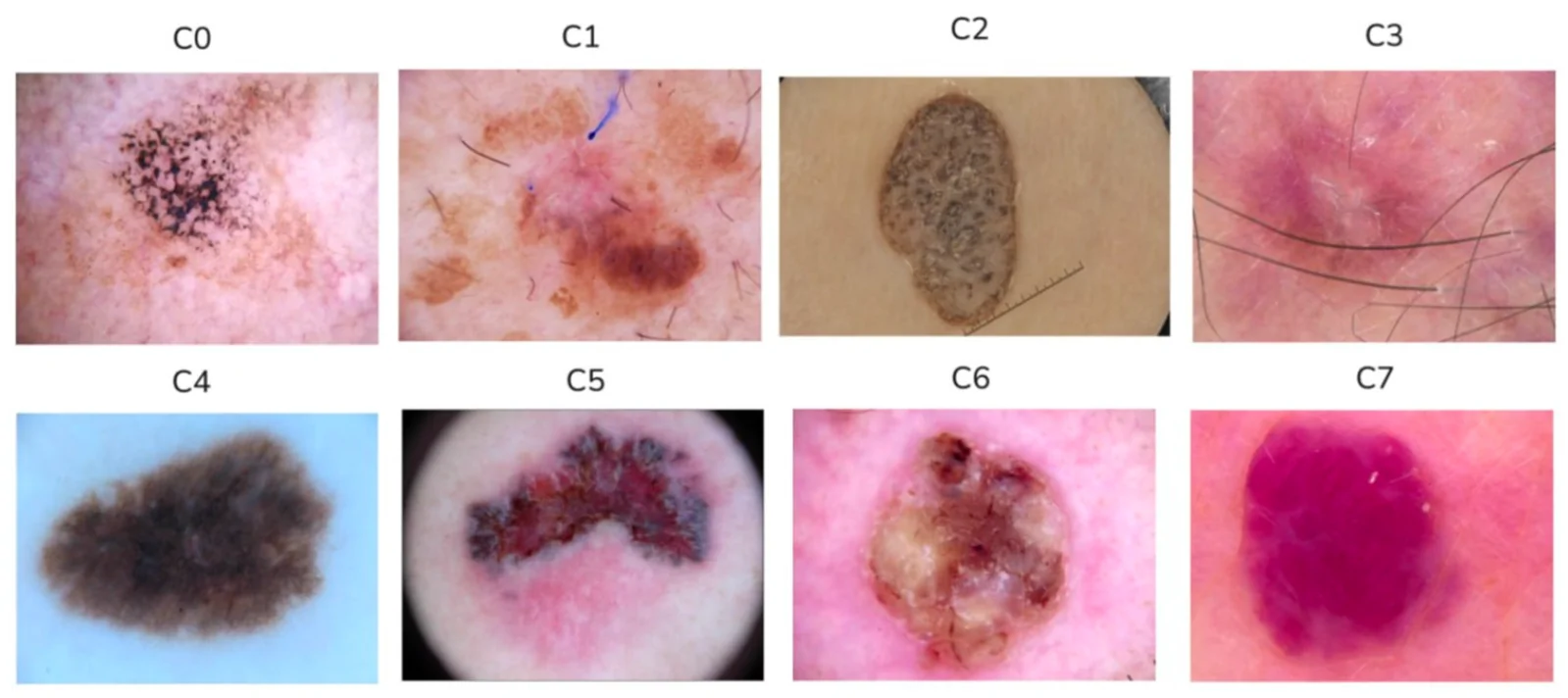
Representative images from each class of the replication dataset. (**C0**) actinic keratosis; (**C1**) cell carcinoma; (**C2**) benign keratosis-like lesions; (**C3**) dermatofibroma; (**C4**) melanocytic nevi; (**C5**) melanoma; (**C6**) squamous cell carcinoma; (**C7**) vascular lesions.

An open dataset of skin lesions from Kaggle [14] (version 1; license CC0: Public Domain; accessed on 20 June 2026) was used as the second dataset. It contained 13,202 RGB images of 14 skin-image categories: actinic keratosis, basal cell carcinoma, benign keratosis-like lesions, chickenpox, cowpox, dermatofibroma, hand, foot and mouth disease (HFMD), healthy skin, measles, melanocytic nevi, melanoma, monkeypox, squamous cell carcinoma, and vascular lesions. The preprocessing was intended to screen duplicate and potentially non-independent content. After preprocessing, including perceptual-hash screening at the reported similarity threshold of 0.95 and manual review by two annotators of classes whose size exceeded the number of distinct source images, 6786 images in 8 classes remained. Perceptual similarity identifies candidate duplicates or related images but does not by itself establish synthetic provenance. The retained class distribution is shown in Figure 4 and Table 2, where C0 is actinic keratosis, C1 is basal cell carcinoma, C2 is benign keratosis-like lesions, C3 is dermatofibroma, C4 is melanocytic nevi, C5 is melanoma, C6 is squamous cell carcinoma, and C7 is vascular lesions. Examples of each class of samples are presented in Figure 5.

### 2.2. Deep Learning Neural Networks

We evaluated twelve pretrained neural-network configurations on the two datasets: ResNet34, ResNet50, ResNet101 [16], ViT-base-patch16-224 [17], DeiT-base-patch16-224 [18], beit-base-patch16-384 [19], DenseNet161, DenseNet201 [20], VGG19 [21], Inception [22], Xception [23], ConvNeXt [24]. To assess the effect of randomness rather than eliminate it, the training of each model was repeated 20 times. In each repetition, the model was reinitialized from the pretrained configuration. No random seeds were explicitly set for the random-number generators in Python 3.9, NumPy 1.24.0, or PyTorch 1.13.1 during the evaluated runs, and the repetition indices were not used as such seeds. The data loaders for training and evaluation were prepared once for each augmentation level and reused across all iterations, without creating a new training–validation split for each run. The results are reported as the mean and standard deviation across the 20 repetitions. No pairing based on identical random-number-generator seeds across different augmentation levels was implemented. This source–derivative constraint should not be confused with grouping all photographs from the same patient or website. Reuse of seed identifiers across enrichment levels also does not, by itself, demonstrate identical source membership in the internal partitions. Our goal was to compare architecture-specific responses to the composite enrichment protocol, including the class-wise response of classes that were underrepresented before undersampling.

**Figure 6:**
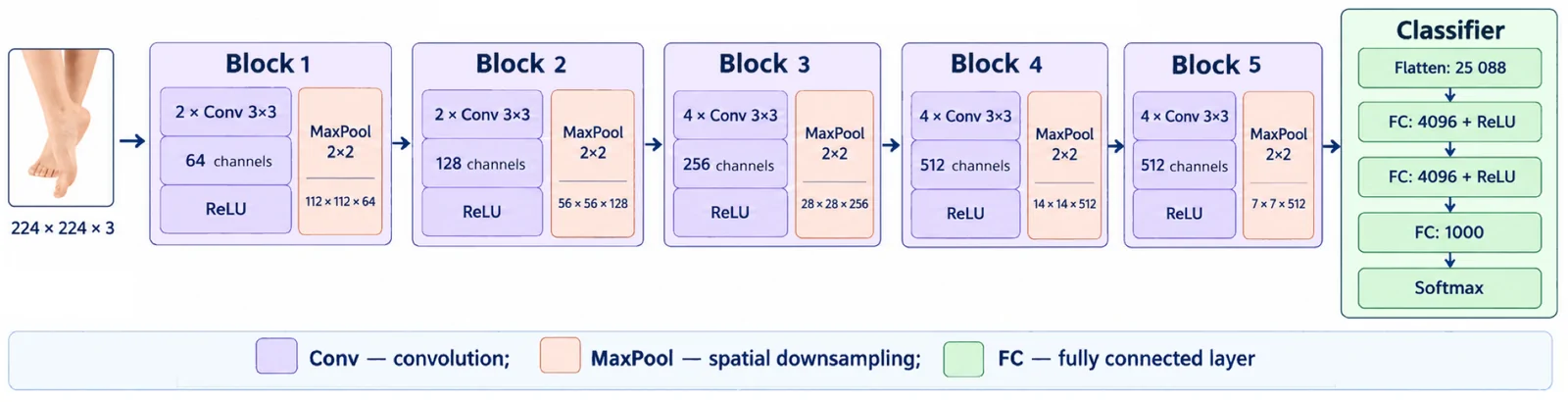
Architecture of VGG19.

The VGG architecture (Figure 6) [21] is one of the first widely adopted deep convolutional networks, introduced to study the effect of depth on recognition performance. Its main design principle is to build a hierarchical representation of images by stacking uniform convolutional blocks, each composed of multiple 3 × 3 convolutional layers followed by 2 × 2 max pooling, and then to classify these features using a series of fully connected layers. This modular, depth-based structure enables the network to learn increasingly complex visual patterns in successive blocks, making VGG a popular choice for transfer learning and feature-extraction in downstream computer-vision tasks.

**Figure 7:**
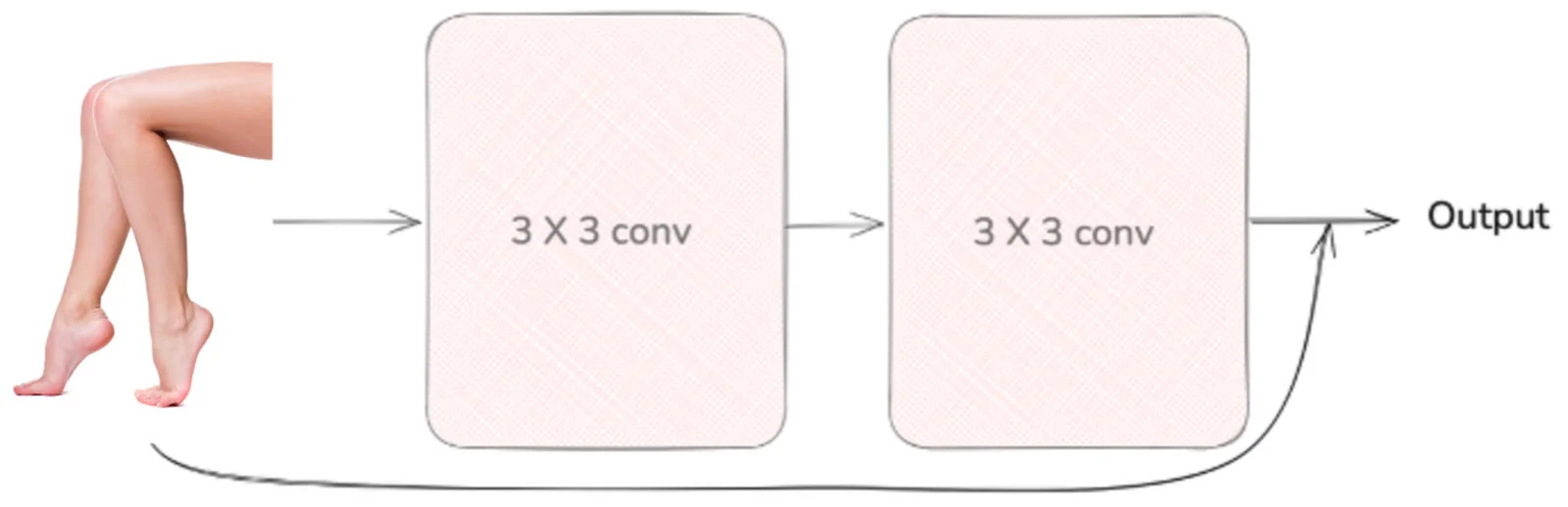
Architecture of a residual block.

ResNet models use residual connections to facilitate the optimization of deep convolutional networks [16]. Rather than learning an unreferenced mapping, the stacked layers learn a residual function relative to their input. A residual block is shown in Figure 7.

**Figure 8:**
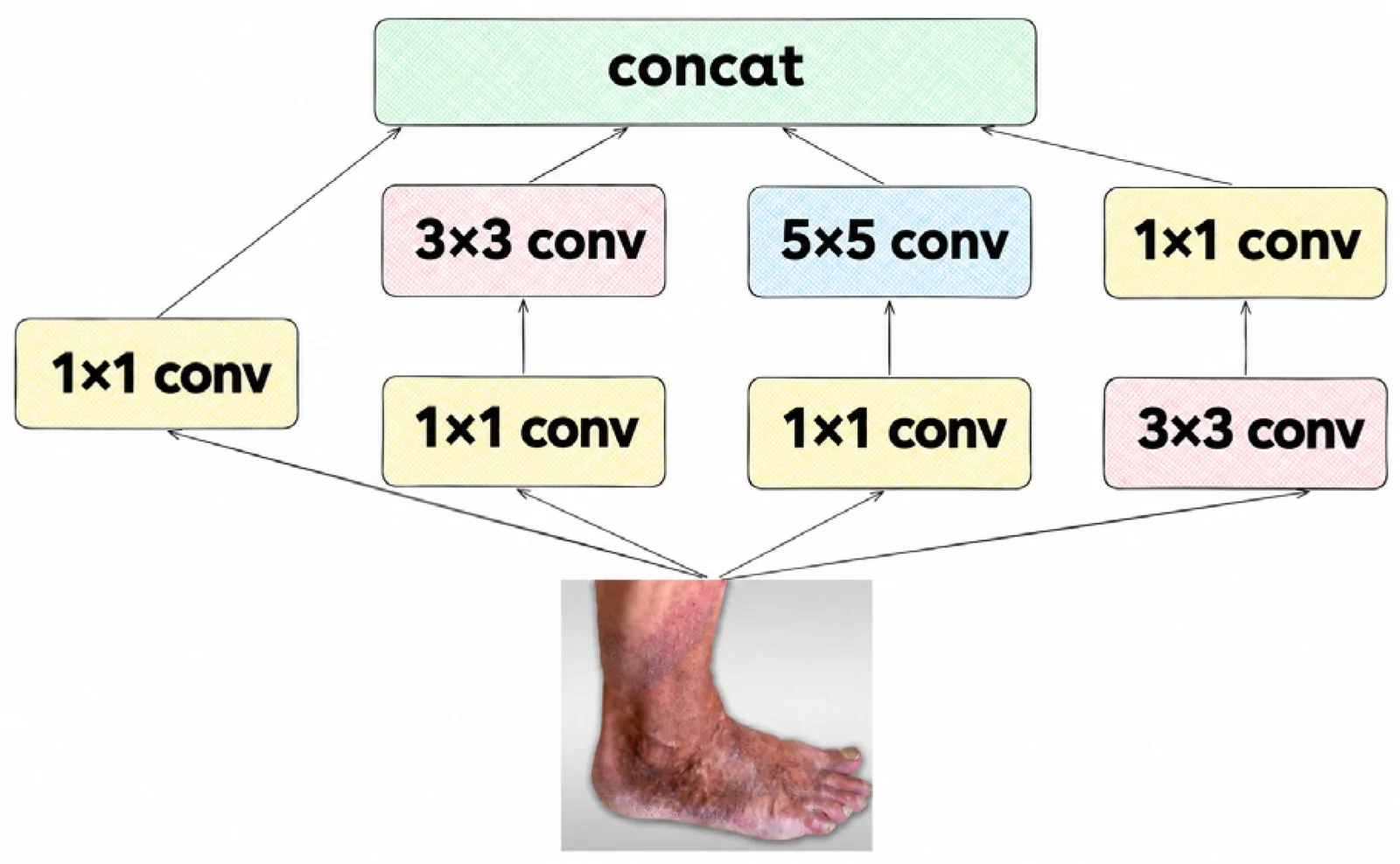
Architecture of an Inception module.

The Inception architecture was introduced in GoogLeNet by Szegedy et al. [22]. Inception modules process feature maps through parallel convolutional and pooling branches and concatenate their outputs along the channel dimension. The use of 1 × 1 bottleneck convolutions reduces computational cost. Figure 8 illustrates a basic Inception module.

**Figure 9:**
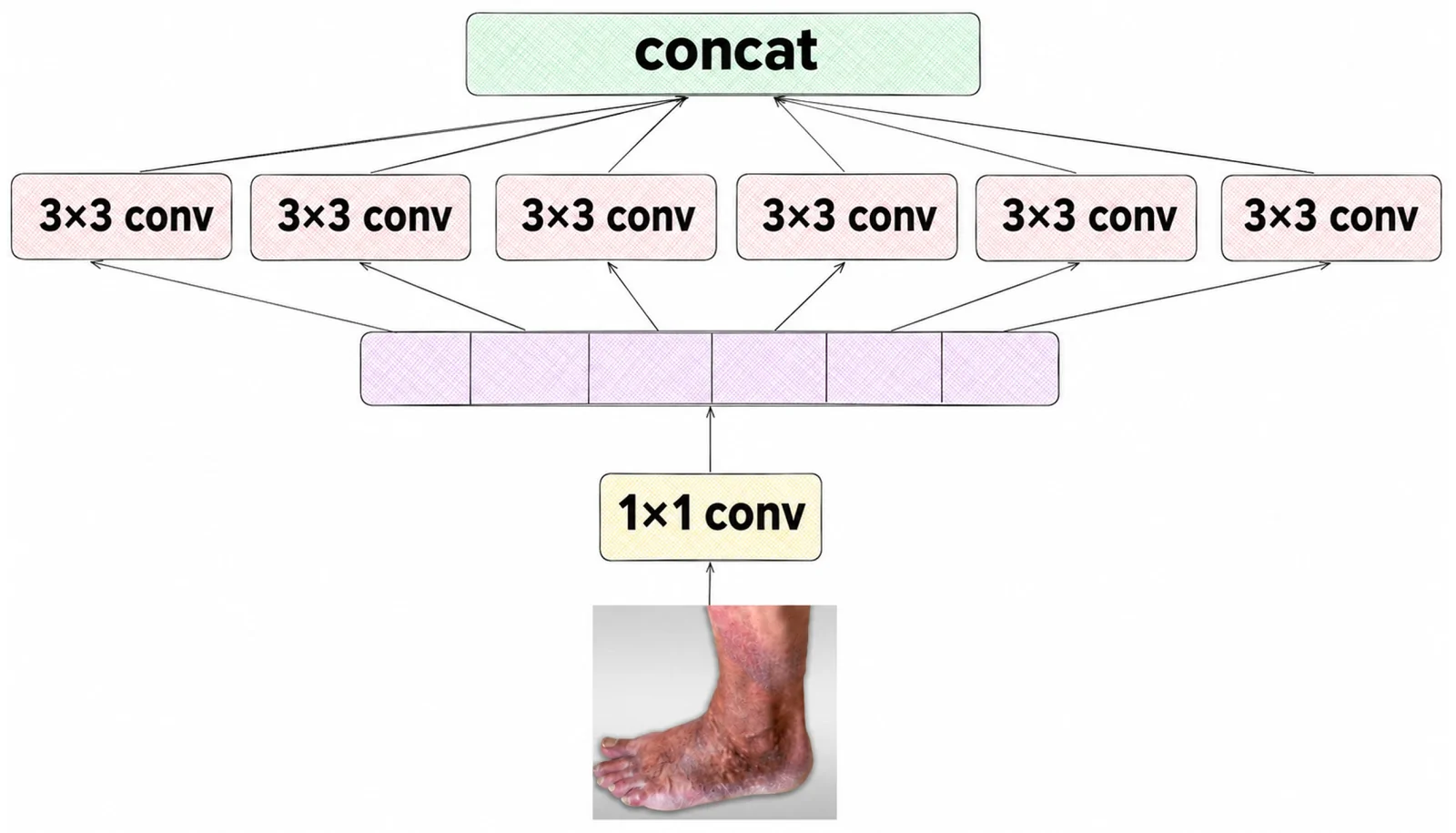
Architecture of an Xception block.

Xception, proposed by Chollet [23], evolves Inception by fully separating spatial and channel-wise convolutions via depthwise separable convolutions in Xception blocks, based on the extreme inception hypothesis that channel mixing works better independently from spatial filtering. A depthwise separable convolution applies a spatial convolution independently to each input channel, followed by a 1 × 1 pointwise convolution that mixes channels [23]. These separable convolutions replace Inception’s parallel branches, with the middle flow module repeated 8 times between the entry and exit flows. Efficiency stems from depthwise separable convolutions, which require far fewer multiply-adds than standard convolutions. Note that Xception itself has approximately the same number of parameters as Inception-V3, and the reported gain comes from more efficient use of these parameters. The architecture of the Xception block is shown in Figure 9.

**Figure 10:**
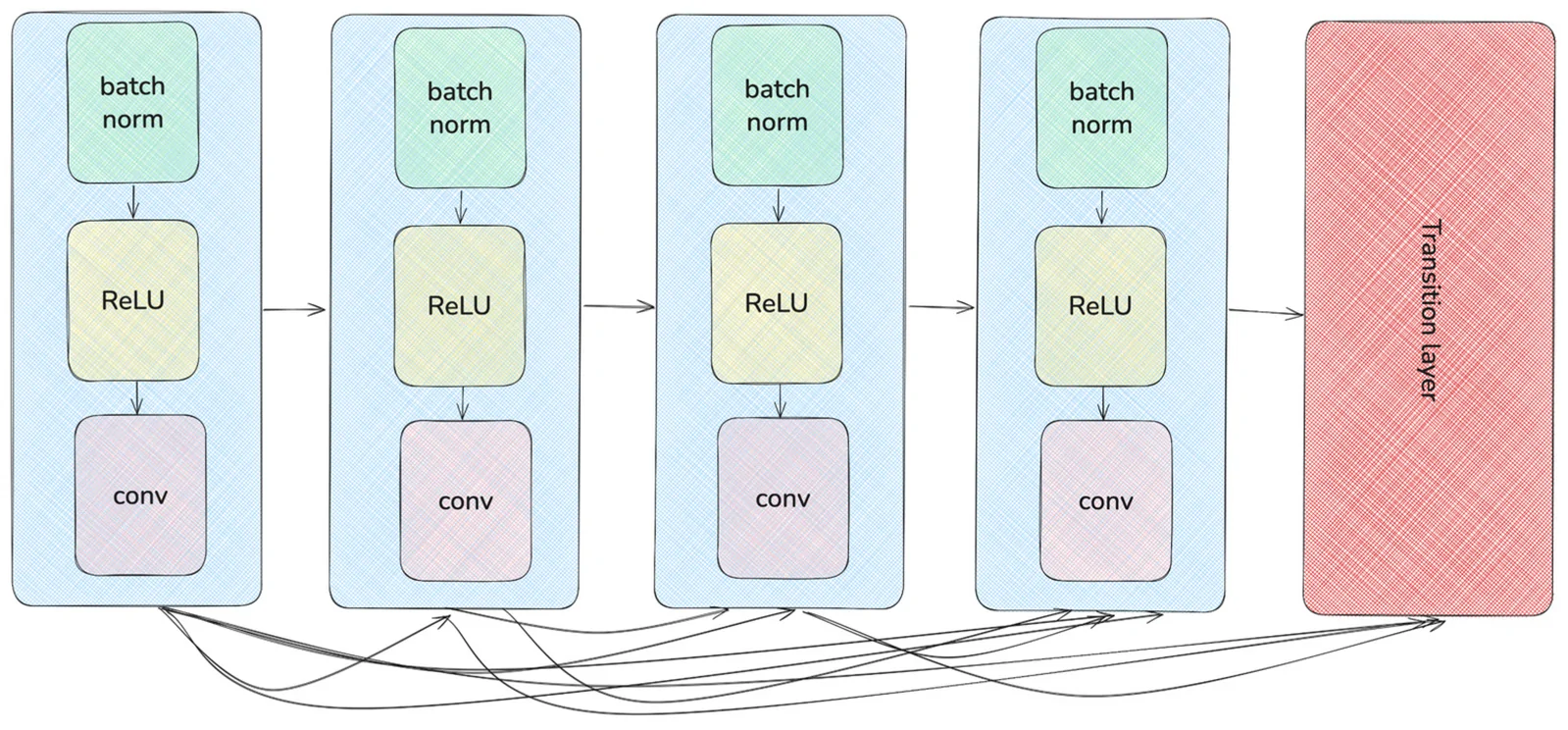
Architecture of a dense block.

DenseNet connects each layer to all subsequent layers within a dense block by concatenating feature maps, rather than adding them as in a residual connection [20]. This dense connectivity promotes feature reuse. A dense block is shown in Figure 10.

**Figure 11:**
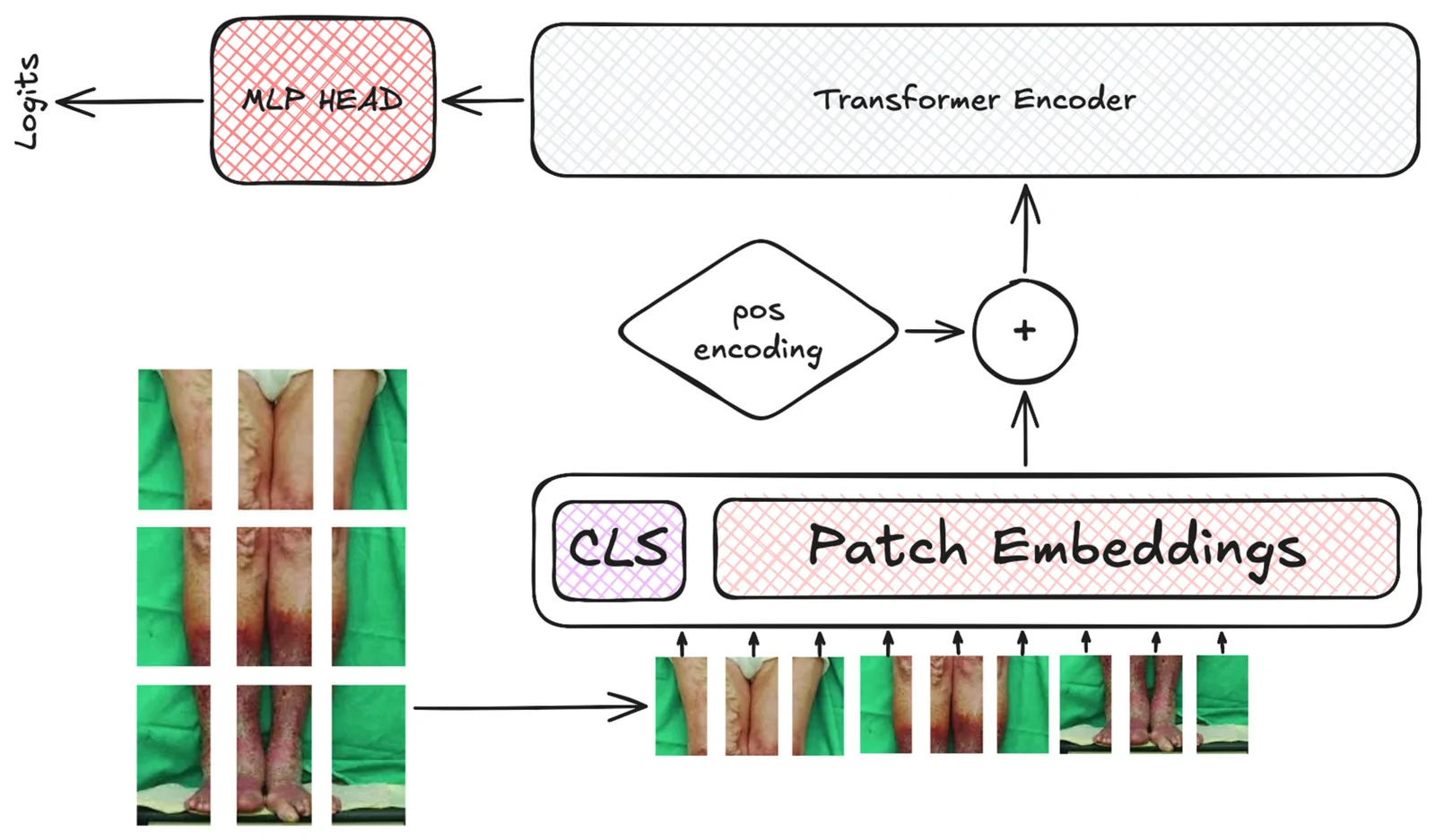
Example of a vision-transformer architecture.

Vision transformer (ViT), data-efficient image transformers (DeiT) and bidirectional encoder representation from image transformers (BEiT) are transformer-based approaches to image recognition [17,18,19]. ViT represents an image as a sequence of embedded patches processed by self-attention layers. DeiT introduces data-efficient training and attention-based distillation, whereas BEiT uses masked image modeling for pretraining. Figure 11 shows a generic vision-transformer architecture.

**Figure 12:**
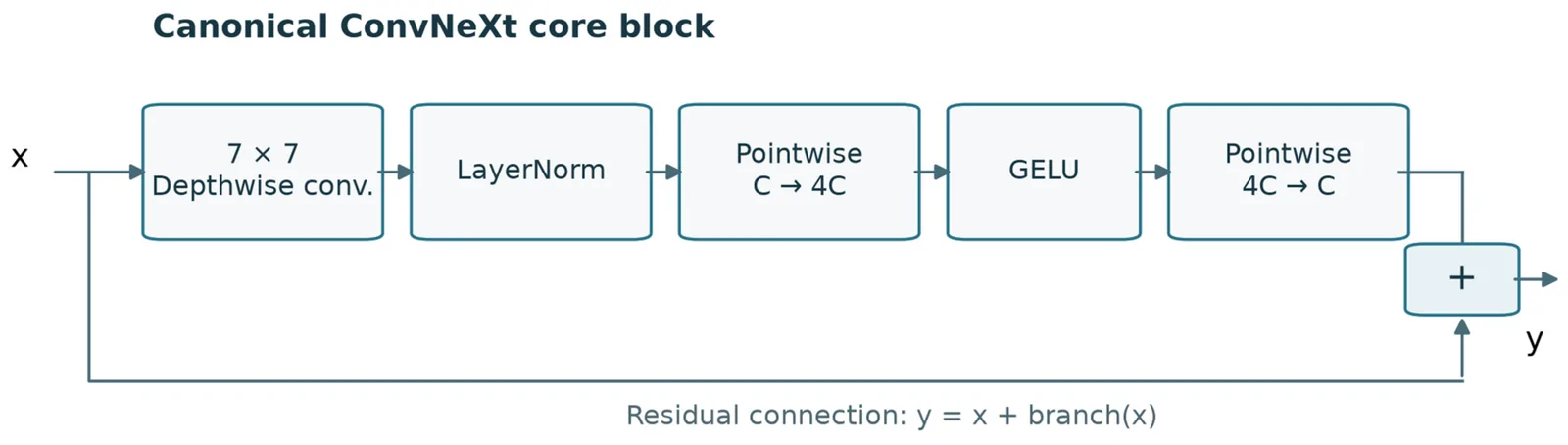
Schematic ConvNeXt residual block. The feature branch contains a depthwise convolution, layer normalization, pointwise channel expansion, GELU activation and pointwise projection. The branch output is added to the residual connection. The schematic shows the canonical core operations [24]. Optional LayerScale and stochastic-depth settings are not verified experimental implementation flags in this diagram.

ConvNeXt, introduced by Liu et al. [24], modernizes CNNs to rival vision transformers like Swin transformer on image classification and vision tasks, using pure convolutions with transformer-inspired design choices and no self-attention. It adopts a staged macro-architecture with four downsampling stages, each stacking ConvNeXt blocks in a transformer-like compute distribution to build global representations as resolution drops and channels grow. The canonical core ConvNeXt block applies a large-kernel 7 × 7 depthwise convolution, channel-wise LayerNorm, pointwise channel expansion, GELU and pointwise projection, followed by residual addition. LayerScale and stochastic depth are optional implementation components. Figure 12 shows the core operations. A patchify stem starts with a 4 × 4 stride-4 convolution and LayerNorm, mimicking transformer patch embeddings to coarsen input from 224 × 224 × 3. Stages alternate blocks and downsampling, ending in global average pooling and a linear classifier; it matches Swin performance at similar FLOPs with better throughput for detection and segmentation. A ConvNeXt block is shown in Figure 12.

### 2.3. Augmentation Methods

*Shift, Scale, and Rotate (Affine Transformations)*. This transformation randomly applies a combination of affine operations: translation, scaling and rotation.

Those methods help simulate variations in object position, orientation, and scale that might naturally occur in real-world conditions.

*Random Crop.* This operation extracts a random spatial region from an image before architecture-specific resizing. Random cropping can reduce reliance on the absolute positions of image features.

*Horizontal Flip.* Images were horizontally flipped to generate their mirrored counterparts, thereby increasing variation in object orientation. Such flipping is especially useful when the target objects have no inherent directional bias.

*RGB Shift.* To simulate variations in lighting and sensor characteristics, random shifts were applied independently to each color channel (red, green and blue). This technique can improve robustness to color and illumination changes in different environments.

*Random Brightness and Contrast Adjustment.* This transformation randomly varies image brightness and contrast to simulate changes in acquisition conditions. This adjustment can reduce overfitting to specific lighting conditions in the training set.

Synthetic images were generated offline using geometric and photometric transformations implemented with Albumentations. The retained augmentation code did not explicitly set a random seed or record the installed library version. No GANs, diffusion models, or other image generators were trained. Each resulting image is derived from a source image and inherits its class label. Table 3 lists the transformation ranges and application probabilities: shifts of up to 5% along each axis, scaling factors from 0.5 to 1.5, rotations between −30° and 30°, cropping covering 80–100% of the image area, horizontal flipping, RGB value shifts in the range of −15 to 15, and brightness and contrast adjustments within the range of −0.2 to 0.2. These transformations were intended to preserve the source-image labels. Label preservation, overfitting and potential leakage require separate assessment and cannot be ruled out solely by the dataset-construction procedure.

### 2.4. Training Procedure

Input images were resized to the architecture-specific resolutions listed in Table 4. Absolute performance comparisons should therefore be interpreted as comparisons of the evaluated pretrained model and training configurations, not as effects of architecture alone. In the verified skin-lesion implementation, the same evaluation loader was used during training to select the checkpoint and afterwards to calculate its reported metrics. The classical CNN checkpoints were selected by the highest mean batch accuracy, whereas ViT, DeiT, BEiT and ConvNeXt checkpoints were selected by the lowest mean batch loss. No separate untouched test evaluation was performed by these loops. The resulting estimates, including maxima across enrichment levels, are therefore selection-dependent descriptive summaries rather than independent test estimates.

Training was performed on a workstation with an AMD Ryzen 5 5600 CPU (AMD, Taiwan Semiconductor Manufacturing Company, Taiwan), an NVIDIA RTX 2070 Super GPU (NVIDIA Corporation, Santa Clara, CA, USA; 8 GB VRAM) and 16 GB RAM, using PyTorch 1.13.0, CUDA 12.0.

Table 4 lists the reported training hyperparameters. Batch sizes spanned the range from 2 to 64, primarily constrained by available GPU memory. These constraints imply that differences in optimization dynamics, gradient noise, normalization, and variability across runs may stem from multiple sources beyond the architecture itself.

Factors such as pretrained weights, input resolution, parameter count, and model-specific settings further complicate isolating architecture-family contributions. For this reason, within-model comparisons offer a more straightforward basis for interpretation than cross-family comparisons.

The comparison presented here is best viewed as a comparative exploration rather than a fully controlled benchmark under matched compute or tuning budgets.

### 2.5. Metrics

Model quality was evaluated with accuracy, macro-averaged precision, macro-averaged recall and macro-averaged F1. TP, FP, TN and FN denote true/false positives and negatives computed per class in a one vs. rest manner, and all values are reported as the mean over 20 runs with the run-to-run standard deviation (SD). In Table 5 and Table 6, ± denotes the run-to-run *SD*, and *SE* = *SD*/√20 describes uncertainty in the mean over independent runs conditional on the fixed data. For 20 runs, *SE* = 0.22361 × *SD*; a pointwise t-based 95% confidence interval for an individual configuration mean is *mean* ± 0.46801 × *SD*. These intervals do not correct for selection among enrichment levels and are distinct from confidence intervals for paired effects. Statistical significance does not establish that an accuracy difference is clinically or practically meaningful.

*Accuracy* is the proportion of correctly classified observations among all evaluated observations:

(1) $$\mathrm{Accuracy}=\frac{\mathrm{Correct classification}}{\mathrm{All classification}}$$

*Accuracy* is reported on the fixed, class-balanced test set and is interpreted together with class-wise precision, recall and F1. For K classes, macro-precision, macro-recall and macro-F1 are the unweighted means of their corresponding class-wise scores; a macro-average is not a per-class metric. Balanced accuracy is macro-recall in this multiclass setting. If every test class has exactly the same number of images, accuracy and balanced accuracy coincide; small differences may arise from integer rounding of class counts. The present report does not provide a complete assessment of class-wise specificity, confusion matrices, macro-AUPRC or probability calibration.

One vs. rest precision–recall curves and AUPRC require continuous class scores, whereas log loss, the multiclass Brier score and probability calibration require class-probability estimates. No numerical probability-based assessment or matched comparison with class weighting and oversampling is reported in the present study.

For a given class, *precision* is the proportion of predictions assigned to that class that are correct:

(2) $$\mathrm{Precision}=\;\frac{\mathrm{True Positive}}{\mathrm{True Positive}+\mathrm{False Positive}}$$

For a given class, *Recall* (sensitivity or true positive rate) is the proportion of observations belonging to that class that are correctly identified:

(3) $$\mathrm{Recall}=\;\frac{\mathrm{True Positive}}{\mathrm{True Positive}+\mathrm{False Negative}}$$

For each class, the F1 score is the harmonic mean of its precision and recall. Macro-F1 is the unweighted arithmetic mean of the class-wise F1 scores; it is not the harmonic mean of macro-precision and macro-recall.

(4)  F1 =2 ⋅ Precision⋅Recall Precision+ Recall

Statistical analysis. Each configuration was trained 20 times. We report the mean and run-to-run standard deviation (*SD*); the standard error is *SE = SD*/√20. These quantities describe training randomness conditional on the fixed evaluation data, not uncertainty across independent patient cohorts. The original exploratory analysis used Welch’s two-sample *t*-test and Holm correction across twelve selected model-level contrasts within each dataset [25]. This correction does not account for selecting the highest test-set mean among eleven levels, so its significance counts are not confirmatory evidence of optimal augmentation.

A selection-aware analysis must include all ten nonzero-level versus baseline contrasts for every architecture, yielding 120 contrasts per dataset for the primary metric, or define an explicitly justified combined family across datasets. When runs can be matched by initialization and source-group split, effects should be estimated from within-pair differences, *d_i_* = *M_i_*(*r*) *− M_i_*(0), rather than an independent-sample approximation. A pointwise 95% confidence interval is $\bar{d\;}\;\pm t_{0.975;19}\mathrm{SE}$. This is not a simultaneous interval. Marginal means and SDs do not determine *SD* because the within-pair covariance is also required. The reported nested F-tests for ABVR are treated as exploratory fit diagnostics, not independent predictive validation.

### 2.6. Augmentation as Biased Variance Reduction: An Exploratory Model

We use “distributional mismatch” rather than “label shift” for the composite effect represented by the model. Classical label shift concerns changes in class priors under invariant class-conditional distributions. Our enrichment protocol may alter class priors and class-conditional image characteristics simultaneously; geometric and photometric perturbations may also introduce artifacts or label noise. These mechanisms are not empirically separated by the fitted coefficient.

The experimental protocol varies a nominal training-set expansion level while changing both real and transformed additions. We use ABVR (augmentation as biased variance reduction) as an idealized risk model and as motivation for a descriptive accuracy-response curve. The idealized derivation assumes a baseline distribution and a single added-sample distribution; it is not an exact probabilistic description of the mixed-addition protocol. Its assumptions and algebraic consequences are distinguished below from empirical observations and independently validated predictions.

For the idealized derivation only, let *P* denote the baseline joint distribution of images and labels, let *T* denote a randomized augmentation operator and let $\tilde{P}$ = *T·P* denote the transformed-sample distribution. Assume *n* baseline real observations and αn transformed observations, with no additional real observations. Hence the effective training distribution for model m is the mixture:

(5) $$Q\left(\alpha \right)=\left(1-\lambda \left(\alpha \right)\right)P+\lambda \left(\alpha \right)\tilde{P},\;\;\lambda \left(\alpha \right)=\frac{\alpha }{1+\alpha }.$$

Under this idealization, α is a transformed-to-baseline ratio and Equation (5) defines λ = α/(1 + α). For the actual experiment, the expansion level *r* and transformed fraction *f_s_* are defined separately in Section 2.1. Its empirical mixture is *Q_r_* = (*N*_0_*P*_0_ + *NᵣPᵣ* + *N_s_P_s_*)/(*N*_0_ + *Nᵣ* + *N_s_*), where *P_s_* is supported on the selected classes. Equating *f_s_* with *r*/(1 + *r*) would require *Nᵣ* = 0.

Within the idealized synthetic-only addition scheme, *λ* lies in [0, 1/2] for *α* in [0, 1], and d*λ*/d*α* = (1 + *α*)^−2^ decreases by a factor of four over this interval. This nonlinear mapping is a property of the idealized mixture and is not a measurement of saturation in the actual mixed-addition experiment. For that experiment, the transformed-image fraction f_s_ also depends on the number of added real images. We denote the population risk of architecture m trained under the idealized mixture by *R_m_(α)*, using the following risk definition:

$$R_{m}\left(\alpha \right)R_{P}=\left({\hat{\theta }}_{m}\left(\alpha \right)\right),\;R_{P}\left(\theta \right)=E_{\left(x,y\right)\sim Pl}\left(f_{\theta }\left(x\right),y\right)\;,$$

where the parameters ${\hat{\theta }}_{m}\left(\alpha \right)$ are obtained by training on the mixture *Q*(*α*); *m* indexes the architecture.

The derivation uses the following idealizing assumptions. (A1) The population surrogate risk is sufficiently smooth in a local neighborhood of a stationary point, with a positive-definite Hessian on the parameter subspace considered. (A2) The distributional perturbation is small enough for a local second-order expansion; the cubic remainder additionally requires suitable higher-order regularity. (A3) Variability across the 20 runs describes training randomness conditional on the fixed data; independence between different enrichment levels is not implied when runs share randomization or source-group partitions. (A4) Holding the test set fixed removes changes in test composition from the comparisons, but does not remove optimization confounding or establish patient-level generalization. The finite-sample variance scaling in Equation (8) is a separate working approximation.

Under (A1)–(A2) the minimizer of the mixture risk admits the influence-function expansion [25]:

(6) $$\theta_{m}^{*}\left(\lambda \right)=\theta_{P,m}^{*}-\lambda H_{m}^{-1}g_{m}+O\left(\lambda^{2}\right),$$

and because ∇*_θ_R_P_* vanishes at $\theta_{P,m}^{*}$, the induced excess population risk is second-order in *λ*:

(7) RPθm∗λ−RPθP,m∗=12λ2Gm+Oλ3,  Gm=gm⊤Hm−1gm≥0.

The scalar $G_{m}=g_{m}^{T}\;H_{m}^{-1}g_{m}$ is a local risk-gradient misalignment functional. Under a positive-definite *H_m_, G_m_* = 0 implies gm = 0; it does not by itself prove label preservation for every image or equality of the underlying distributions. The quadratic term follows from the local perturbation expansion in Equations (6) and (7). The variance contribution *V_m_*/[*n*(1 + *α*)] additionally approximates information growth by sample-count growth. Transformed derivatives share information with their source images, so the number of stored files is not necessarily the effective number of independent observations. Equation (8) combines these assumptions and should therefore be treated as an approximation:

(8) $$E\;R_{m}\left(\alpha \right)=R_{m}^{*}+\frac{V_{m}}{n\left(1+\alpha \right)}+\frac{G_{m}}{2}{\left(\frac{\alpha }{1+\alpha }\right)}^{2}.$$

Equation (8) combines an assumed decreasing variance contribution with a quadratic local distributional-mismatch penalty. It motivates the following conditional consequences of the idealized model. The present design does not directly measure all quantities involved and therefore cannot test every proposed mechanism independently.

Conditional implication for the optimum. For *b_m_ = V_m_/n ≥* 0 and *G_m_* > 0, minimization over 0 ≤ *λ* ≤ 1/2 gives:

λₘ* = *min*(*bₘ*/*Gₘ*,1/2), αₘ* = λₘ*/(1 − λₘ*) (9)

For *G_m_* = 0 and *b_m_* > 0, the optimum is λ_m_* = 1/2 and α_m_* = 1. If *b_m_ = G_m_* = 0, all investigated levels have equal modeled risk.

An interior optimum depends on the architecture- and dataset-specific ratio *V_m_/(nG_m_)*. Different ratios can nevertheless produce the same boundary optimum after restriction to the investigated interval. Thus, ABVR permits architecture-dependent optima but does not prove that a common optimum is impossible. Likewise, changing the baseline sample size can affect the optimum only under the additional assumption that the other model quantities remain fixed.

Conditional bound on the idealized risk gain. The constrained optimum gives:

(10) $$\Delta R^{*}\;=\left\{\begin{array}{c} \begin{array}{cc} {b_{m}}^{2}/\left(2G_{m}\right) & \begin{array}{cc} \mathrm{for} & G_{m}\geq 2b_{m},\;G_{m}>0 \end{array} \end{array}\\ \begin{array}{ccc} b_{m}/2-G_{m}/8 & \mathrm{for} & 0\leq G_{m}<2b_{m} \end{array}\\ \begin{array}{ccc} 0 & \mathrm{for} & b_{m}=G_{m}=0 \end{array} \end{array}\right.\;$$

For *b_m_ = V_m_/n*, the modeled gain is Δ*R(λ) = bₘλ − Gₘλ*^2^/2. At the constrained optimum:

ΔR∗=bm22Gm≤bm4 when Gm≥2bm and Gm>0bm2−Gm8≤bm2 when 0≤Gm<2bm0whenbm=Gm=0

The quarter-component bound therefore applies to an interior optimum or the transition point, not to every boundary optimum. The general bound on the investigated interval is $\Delta R^{*}\leq \frac{b_{m}}{2}$. These are bounds on an idealized expected-risk contribution, not on observed accuracy gains or the empirical run-to-run variance in accuracy. Power calculations are separate from this bound and depend on effect size, the variability of paired differences or independent observations, and the multiplicity adjustment.

Heuristic implication for variability. The covariance of the per-sample gradient under the mixture is C(*λ*) = (1 − *λ*)*C_P_* + *λC_T_*_⋅*P*_ + *λ*(1 − *λ*)g*g*^⊤^, the last term arising because the two components have different means. Augmentation therefore injects variance as well as removing it, consistent with the broader moment-based perspective of [26], which does not derive the present across-run accuracy-variance approximation. Propagating this through a sandwich covariance approximation gives, to leading order [27]:

(11) $$\frac{s_{m}{\left(\alpha \right)}^{2}}{s_{m}{\left(0\right)}^{2}}\approx \frac{1+\gamma_{m}\lambda \left(1-\lambda \right)}{1+\alpha },\;\;\gamma_{m}≲\frac{\kappa \left(H_{m}\right)\;{\left\|g_{m}\right\|}^{2}}{\lambda_{\min}\left(C_{P,m}\right)},$$

Here *κ*(*H_m_*) denotes the Hessian condition number. Equation (11) is a heuristic approximation requiring assumptions about component gradient covariances and the propagation of parameter variability to the reported metric. Neither conditioning nor the gradient mismatch was measured in this study. The equation therefore does not establish lower dispersion for transformers, ConvNeXt or models with normalization. Moreover, maximizing *λ(1 − λ)* alone does not maximize the entire expression, because the denominator also depends on *α*. The location of maximal run-to-run dispersion must be assessed from the full response, not inferred from the numerator alone.

Class-wise extension (not an independently tested prediction). Under additional assumptions that permit separate class-wise risks and hold the other model terms fixed, the idealized model suggests a sample-size dependence of the preferred transformed fraction. This does not establish that augmenting an originally underrepresented class improves its recall in a jointly trained multiclass classifier. For a perfectly balanced K-class test set, an isolated recall change ΔRecall_c_, with all other class recalls unchanged, changes accuracy by Δ*Acc* = Δ*Recall_c_*/*K*. Thus, a + 0.07 change in one class contributes +0.01 to accuracy when *K* = 7. The present balanced-pool experiment describes class-wise responses, but does not test a rare-class-recall improvement without loss of overall accuracy.

To summarize the measured accuracy curves, we fit the following ABVR-inspired empirical response function over the nominal expansion level *r*. It has the functional form motivated by Equation (8):

(12) $$A_{m}\left(r\right)=a_{m}-\frac{v_{m}}{1+r}-h_{m}{\left(\frac{r}{1+r}\right)}^{2},\;\;a_{m},v_{m},h_{m}\geq 0,$$

The response function is fitted separately to each model and dataset by weighted least squares over the 11 levels, with weights 1/*SE*^2^ and *SE* = *SD*/√20 (11 points, 3 fitted parameters and nominally 8 residual degrees of freedom). In this empirical curve, *v_m_* and *h_m_* are effective response coefficients; *h_m_* is a curvature or mismatch parameter, not a direct measurement of label shift. The fitted maximizer is an in-sample description.

Under the imposed non-negativity constraints on the fitted coefficients, we propose the following definitions for the case $\hat{h_{m}}>0$:

$$\hat{\lambda_{m}^{*}}=\min\left\{\frac{\hat{v_{m}}}{2\hat{h_{m}}},\frac{1}{2}\right\},\;\hat{r_{m}^{*}}=\frac{\hat{\lambda_{m}^{*}}}{1-\hat{\lambda_{m}^{*}}}$$

Here, $\lambda_{m}=r/(1+r)$ serves as an auxiliary coordinate that captures the total expansion; it should not be interpreted as the exact fraction of transformed images when both real and transformed images are included in the training set.

When $\hat{h_{m}}=0$ and $\hat{v_{m}}>0$, the fitted maximum occurs at *r =* 1. If both coefficients are zero, the fitted curve remains constant. It is worth noting that the nominal nested F-test and the Holm adjustment are used here as exploratory diagnostic tools: the constrained nature of the coefficients and potential dependencies among errors across levels mean these tests should be interpreted with care. Furthermore, the fitted curves are not intended to provide out-of-sample predictions of optimal expansion levels.

The real-only baseline and a matched real-image expansion control address different research questions. Comparisons against the *r* = 0 baseline help illustrate how the implemented enrichment protocol affects performance relative to the original real-image training set. However, since the protocol jointly introduces transformed images, additional real images, and changes to both the training-set size and the optimization exposure, such comparisons cannot cleanly isolate the specific contribution of the transformed images from the other concurrent modifications.

A matched real-image expansion control could offer an additional perspective by enabling a comparison between transformed and real additions under conditions of equal per-class sample counts and comparable optimization budgets. Such matching would not ensure equivalence in all relevant factors: source composition, effective sample size, and gradient covariance might still differ. As a result, the real-image expansion condition would not inherently correspond to zero distributional mismatch, and any observed differences between conditions could not be straightforwardly attributed to a pure quadratic bias term alone.

Finally, the current summary of the fitted results does not incorporate estimates of coefficient uncertainty or independent predictive validation. Consequently, the empirical response curve is best viewed as a descriptive summary of observed performance changes relative to the real-only baseline and across different expansion levels. It is not presented as a validated estimator of an optimal expansion level or as a definitive characterization of the underlying mechanisms driving those changes.

## 3. Results

**Figure 13:**
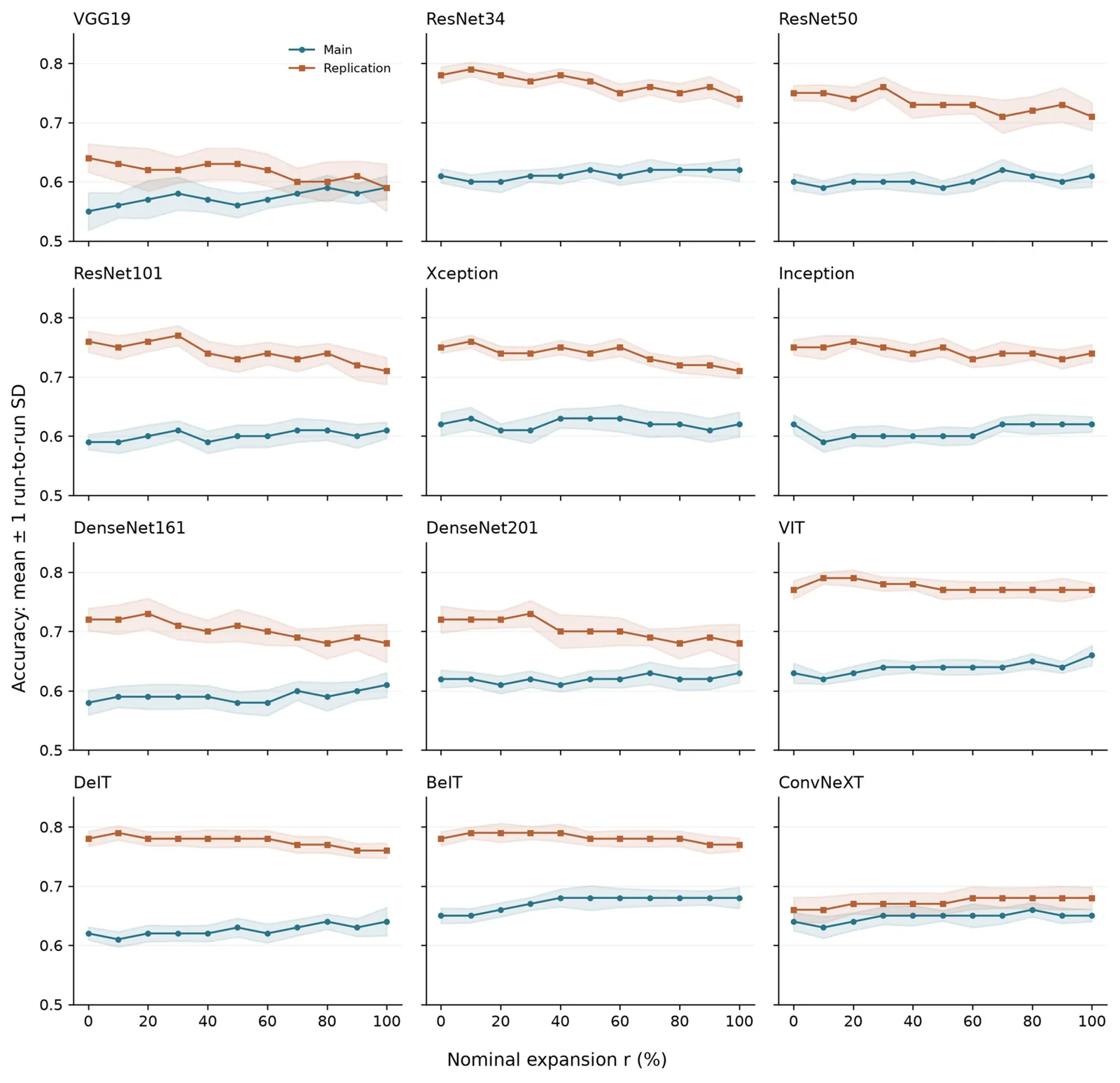
Accuracy responses for twelve configurations on both datasets. Points are the means reported in Table 5 and Table 6; ribbons denote ±1 run-to-run SD from Table 5 and Table 6 (20 runs per point), not confidence intervals. The *x*-axis is nominal expansion r (%). Blue line – the main database; red line – the replication database.

Table 5 and Table 6 report mean accuracy and SD over 20 runs. Figure 13 combines the two dataset responses for all twelve configurations. Class-wise precision and recall panels are provided once, paired by architecture, in Supplementary Section S1.

Table 5 and Table 6 show architecture- and dataset-dependent responses to the composite enrichment protocol. Differences between adjacent levels should be considered alongside both the run-to-run SD and uncertainty in the estimated effects. Under the original exploratory Welch analysis with Holm correction across the selected model-level contrasts, the reported significance counts were 10 of 12 on the main dataset and 4 of 12 on the replication dataset. These counts refer to that original procedure. They do not account for selection over all candidate levels and should not be interpreted as confirmation of an optimal ratio. For a model whose highest mean occurs at 0%, no positive improvement over baseline is implied.

On the main dataset, BEiT increased from 0.65 at 0% to 0.68 from 40% onward; ViT reached 0.66 at 100%, DeiT reached 0.64 at 80%, and ConvNeXt reached 0.66 at 80%. On the replication dataset, several curves flattened or decreased at higher levels. The observed fluctuations and declines do not by themselves identify distribution noise, local feature hierarchies, or another architecture-specific causal mechanism.

The evaluated transformer configurations had lower average run-to-run SD of accuracy than the classical CNN configurations: 0.0138 versus 0.0171 on the main dataset and 0.0133 versus 0.0195 on the replication dataset. The originally reported one-sided Mann–Whitney *p*-values were 0.067 and 0.042, respectively. Thus, only the latter crossed the nominal 0.05 threshold. With three transformer architectures and two datasets, the evidence supports an exploratory tendency rather than a general property of transformers. If the two dataset-level tests are treated as one family, a further Holm adjustment of the reported *p*-values gives approximately 0.084 for each. ConvNeXt had a mean SD of 0.0144 on the main dataset and 0.0189 on the replication dataset, illustrating dataset dependence.

**Figure 14:**
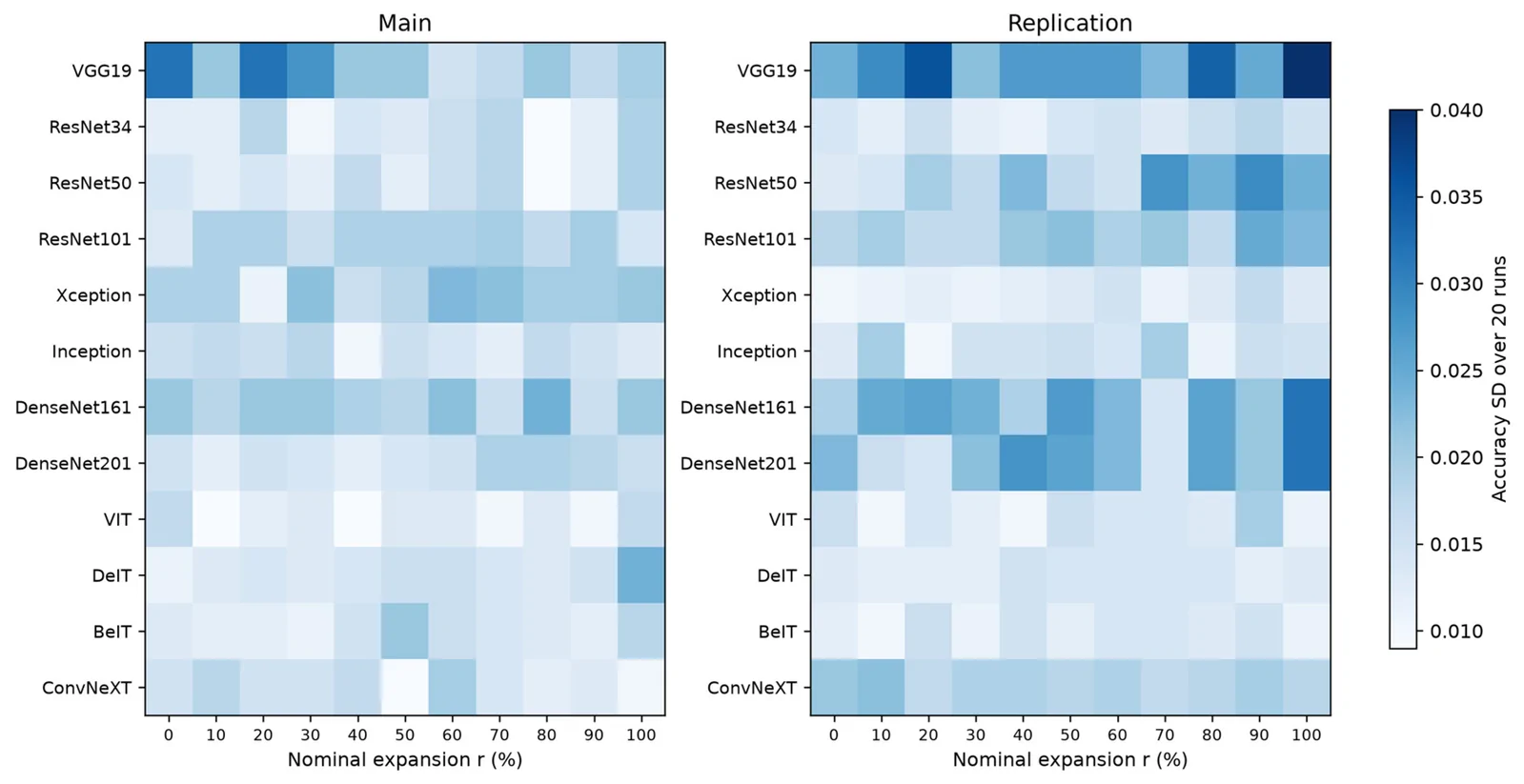
Run-to-run accuracy SD for each configuration and nominal expansion level. Values are reproduced from Table 5 and Table 6 without baseline normalization. Both panels use the same color scale.

Figure 14 combines the run-to-run accuracy SDs for both datasets from Table 5 and Table 6. These are raw SDs, not SD ratios relative to baseline. The original additional dispersion panels are retained in Supplementary Section S2.

Supplementary Sections S3 and S4 contain the original accuracy box plots. They display run-to-run variation on fixed evaluation data, not uncertainty across independent patient cohorts. The original raster panels are retained; quartiles and individual-run values have not been reconstructed from aggregate tables.

## 4. Discussion

### 4.1. Architecture-Specific Responses to Synthetic Data

The highest individual-run metric is not a reliable summary of a configuration. Run-to-run statistics incorporate the combined effects of the reported training randomization and internal data splitting; their separate contributions were not measured.

Different architectures exhibited markedly different sensitivities to the inclusion of synthetic images, indicating that augmentation strategies cannot be assumed to transfer uniformly across model families.

The observed run-to-run variability did not show a clear monotonic relationship with network depth. Better optimization conditioning is one possible explanation for some of the differences, but Hessian conditioning and gradient-noise statistics were not measured. Architectural features such as normalization therefore provide hypotheses for further testing, not demonstrated causes of the observed variability. Configuration differences in batch size, resolution and pretraining offer additional explanations that are not separated by this design.

Aggregate performance metrics may not fully reflect class-specific effects of enrichment and should not be used alone to establish clinical usefulness. Related work on the SMOTE-AE-CNN-Att framework combines synthetic minority oversampling, autoencoder-based feature learning, CNNs and attention for credit-card fraud detection [28]. This provides a methodological comparison for imbalance-aware evaluation, but differs from transformation-based medical-image augmentation. The systematic review of explainable AI for cancer detection by Toumaj et al. [29] provides complementary context on transparency and trustworthy medical AI. Neither study independently validates the augmentation levels, architecture rankings or ABVR mechanism examined here.

At the 10% level, accuracy improved for 3 of 12 models on the main dataset (VGG19, Xception and DenseNet161: +0.01) and for 5 of 12 models on the replication dataset (ResNet34, Xception, ViT, DeiT and BEiT: +0.01 to +0.02), while the remaining models were unchanged or deteriorated. Inception on the main dataset decreased from 0.62 to 0.59. Therefore, 10% is a low-expansion candidate within the tested grid, not a generally safe proportion or a non-inferiority threshold. Its usefulness should be assessed on internal validation data for the specific configuration.

On the main dataset, BEiT increased from 0.65 at baseline to 0.68 at the 40% nominal expansion level and remained at that rounded mean. DeiT reached 0.64 at 80% on the main dataset and 0.79 at 10% on the replication dataset. These are descriptive maxima and plateaux on the evaluated grid, not independently established saturation thresholds.

The evaluated transformer configurations showed relatively low average run-to-run variability under the present settings, whereas several CNN configurations were more variable. However, low run-to-run variability and low sensitivity of mean performance to enrichment are different properties. Neither is established as an architecture-family-wide characteristic by this experiment, and both may be affected by batch size, input resolution, pretrained weights and other training choices.

VGG19 showed comparatively high mean run-to-run variability under the evaluated configuration. Its large fully connected head and normalization choices may contribute, but these mechanisms were not tested independently and no causal attribution is made.

On the replication dataset, absolute accuracy was higher (0.59–0.79 versus 0.55–0.68 on the main dataset), yet 10 of 12 models lost 0.01–0.05 accuracy between the 0% and 100% levels. This pattern indicates dataset-dependent responses to enrichment; it does not establish that task difficulty caused changes in variability.

ConvNeXt had mean accuracy SDs of 0.0144 on the main dataset and 0.0189 on the replication dataset. Its replication-dataset accuracy ranged from 0.66 to 0.68, compared with 0.76–0.79 for the evaluated transformer configurations. These observations illustrate dataset dependence rather than a general effect of transformer-inspired design.

### 4.2. Quantitative Interpretation Through the ABVR Model

**Figure 15:**
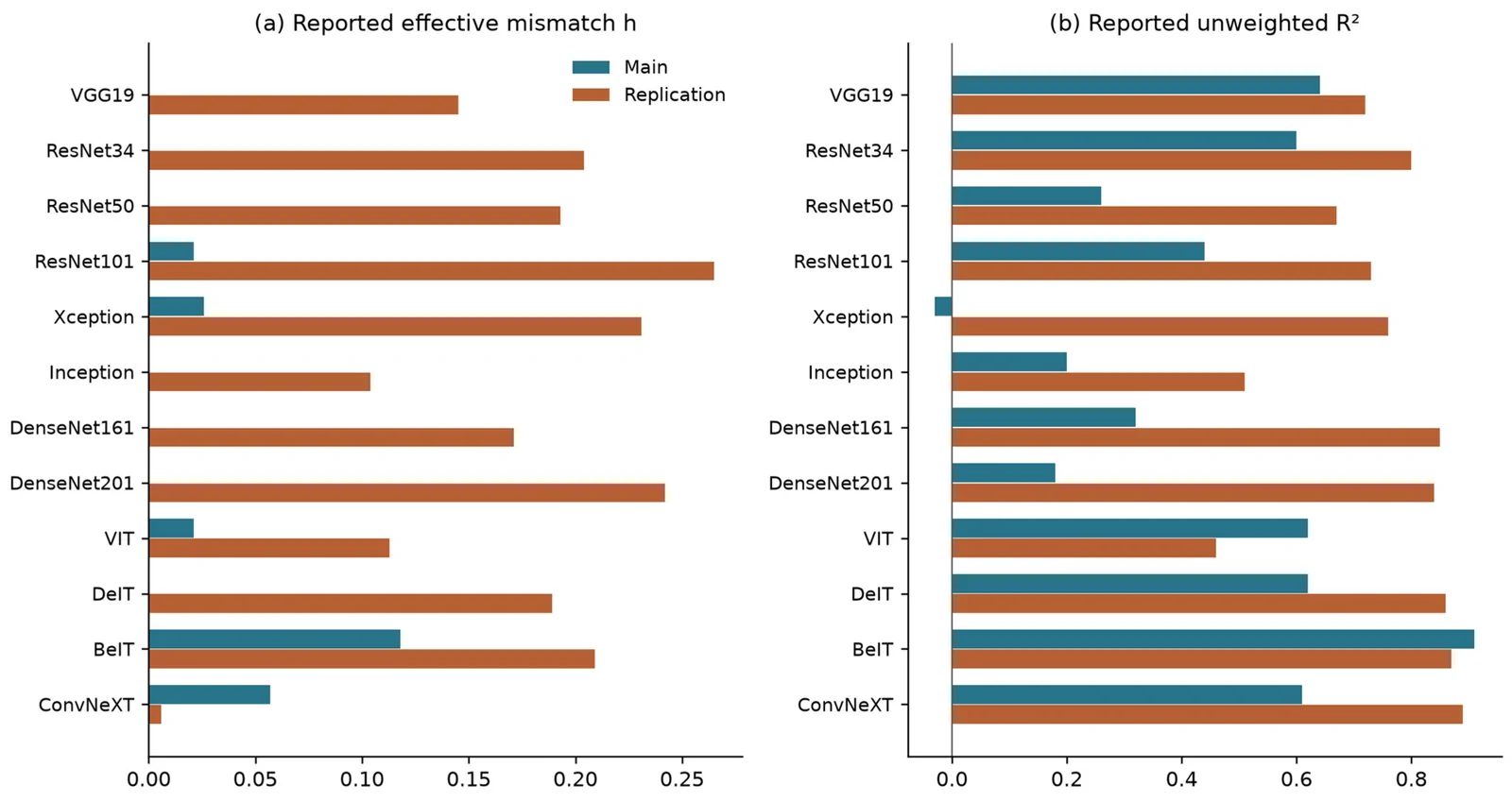
Reported ABVR-inspired fit summaries from Table 7: (**a**) effective mismatch coefficient h; (**b**) unweighted R^2^. These are submitted coefficients and fit statistics, not a new fit or independent validation. No coefficient uncertainty is implied by the bars.

The three-parameter ABVR-inspired curve was fitted to the eleven measured levels of each series in Table 5 and Table 6. Table 7 reports the fitted parameters and Figure 15 summarizes the reported mismatch coefficients and unweighted fit R^2^ values. For the rounded coefficients in Table 7, the median fitted h is 0 on the main dataset and 0.191 on the replication dataset. A fold comparison of these medians is therefore undefined, and these descriptive summaries do not measure clinical label noise or establish a causal explanation of the dataset effect. Uncertainty in fitted coefficients, boundary estimates and differences in dataset composition limit this interpretation.

Differences in the clinical information retained by geometric and photometric transformations are plausible explanations for dataset-dependent responses. However, the present experiments did not quantify transformation-induced label changes by independent expert reassessment. The fitted optima, which are mostly 100% on the main dataset and range from 0% to 100% on the replication dataset, are summaries of the same curves used for fitting. Their proximity to particular observed levels is therefore in-sample agreement, not prediction without further tuning or evidence that 10% is beneficial across architectures.

In the original exploratory fitting analysis, the nominal F-test against a constant rejected for all 12 architectures on the replication dataset (10 of 12 after Holm correction), with a reported median R^2^ of 0.78; on the main dataset it rejected for 6 of 12 (1 of 12 after correction), with a median R^2^ of 0.52 calculated from the rounded values in Table 7. These reported statistics describe the original fitting procedure and should not be interpreted as independent validation of ABVR. Non-rejection does not establish a constant true response, but it does not prove that low power is the sole explanation either. Parameter constraints, correlations between levels, and alternative curve shapes must be considered before making confirmatory model-comparison claims.

The correlation between the fitted mismatch coefficient and mean run-to-run SD was weak and non-significant on both datasets (Spearman ρ = 0.22, *p* = 0.49 on the main dataset; ρ = −0.13, *p* = 0.70 on the replication dataset). This does not support the proposed association in the evaluated configurations. It does not identify optimization conditioning as the dominant remaining cause either, because conditioning was not measured. Direct estimates of Hessian and gradient statistics, together with controlled training comparisons, are required to assess this mechanism.

Fit quality varies across architectures. For Xception on the main dataset, the reported unweighted *R*^2^ is −0.03, so the fitted curve is worse than the sample-mean constant under that unweighted criterion. This is possible for a weighted fit and is not by itself evidence of a computational error. For VGG19, accuracy varied from 0.55 to 0.59 with relatively high run-to-run variability. These examples limit the use of ABVR as a precise predictor of individual levels. Residual diagnostics, uncertainty intervals for coefficients and maximizers, comparisons with constant, linear and alternative nonlinear curves, and genuinely held-out prediction are needed for stronger explanatory or predictive claims.

## 5. Conclusions

This study compared twelve pretrained image-classification configurations under eleven levels of a composite training-data enrichment protocol on two datasets. The observed responses depended on both configuration and dataset, and no single level consistently improved all evaluated models. These findings support configuration-specific assessment of enrichment settings, not a universally optimal or safe proportion. Test-set maxima are descriptive, and the original selected-contrast significance counts should not be treated as confirmatory evidence after selection over levels. Practical interpretation requires effect sizes, appropriate uncertainty estimates and an explicitly justified threshold of meaningful benefit.

Run-to-run variability should be reported separately from uncertainty in mean performance, and architecture comparisons should be interpreted in light of batch size, resolution, pretraining and training-budget differences. ABVR provides an exploratory description of the observed curves, not independently validated evidence of a biological or optimization mechanism. Its idealized risk bounds do not bound empirical accuracy gains through the observed run-to-run variance. Selection of enrichment settings should use development data, followed by evaluation that is independent of the selection procedure.

The balanced-pool design does not directly test whether augmentation corrects rare-class imbalance. A dedicated comparison with class-weighted loss, ordinary oversampling, online augmentation and all-class augmentation is required, using matched source-group splits, class counts and optimization budgets. The *r* = 0 condition provides a real-only baseline. Outcomes for an additional matched real-image expansion control are not reported, so the contribution of transformed additions cannot be fully separated from accompanying enrichment effects. The dermatological replication is not external patient-level validation of the CEAP model. Source-disjoint evaluation, documented patient-level independence and demographic subgroup analyses would be required before clinical deployment or fairness claims. The present findings are limited to a methodological comparison on the evaluated data.

## Figures and Tables

**Table 1:** Class distribution of images of the main dataset.

Class	C0	C1	C2	C3	C4	C5	C6
Number of images	2494	5495	2861	6850	2386	458	427

**Table 2:** Class distribution of images of the replication dataset.

Class	C0	C1	C2	C3	C4	C5	C6	C7
Number of images	882	1154	1221	204	1222	1221	611	271

**Table 3:** Augmentation transformations.

Transformation	Transformation Description	Transformation Params	Transformation Probability
Shift	This transformation applies random translation.	The shift range for both horizontal and vertical translation was (−0.05, 0.05), corresponding to a potential displacement of up to 5% of the image size along each axis.	0.6
Scale	This transformation applies random scaling.	The scale limit was set to 0.5, i.e., the scaling factor was sampled uniformly from [0.5, 1.5] (up to ±50% of the original size).	0.6
Rotate	This transformation applies random rotation.	The rotation range was defined as (−30°, 30°), allowing images to be randomly rotated within this angular range.	0.6
Random Crop	This operation extracts and resizes a random image crop.	A crop covering 80–100% of the image area is resized to 224 × 224 pixels.	0.5
Horizontal Flip	Images were horizontally flipped to generate their mirrored counterparts, thereby increasing variation in object orientation	Has no parameters	0.5
RGB Shift	This technique improves robustness to color and illumination changes in different environments.	The range of value change for each channel was (−15, 15).	0.6
Random Brightness and Contrast Adjustment	This transformation randomly adjusts image brightness and contrast.	Brightness and contrast adjustments are sampled from [−0.2, 0.2].	0.7

**Table 4:** Model-specific training hyperparameters.

Model	Number of Parameters	Image Size	Batch Size	Num of Hidden Layers	Optimizer	Learning Rate
ResNet34	21.8M	224	64	34	AdamW	1 × 10^−4^
ResNet50	25.6M	224	64	50	AdamW	1 × 10^−4^
ResNet101	44.6M	224	64	101	AdamW	1 × 10^−4^
VGG19	143.7M	224	16	19	Adam	1 × 10^−4^
DenseNet161	28.7M	224	8	161	AdamW	1 × 10^−4^
DenseNet201	20.2M	224	8	201	AdamW	1 × 10^−4^
Inception_v3	24M	299	16	42	AdamW	1 × 10^−4^
Xception	22.9M	224	8	126	AdamW	1 × 10^−4^
VIT	86M	224	32	12	AdamW	5 × 10^−5^
DeIT	86M	224	32	12	AdamW	5 × 10^−5^
BeIT	87M	384	2	12	AdamW	5 × 10^−5^
ConvNeXT	88.6M	224	8	36	AdamW	5 × 10^−5^

**Table 5:** Mean accuracy (mean *± SD* over 20 runs; *n* = 20) on the held-out test part of the main dataset. The sign ± denotes the run-to-run standard deviation; the standard error of the mean is *SD*/√20.

Nominal Expansion, %	VGG19	ResNet34	ResNet50	ResNet101	Xception	Inception	DenseNet161	DenseNet201	VIT	DeIT	BeIT	ConvNeXT
0	0.55 ± 0.032	0.61 ± 0.012	0.6 ± 0.014	0.59 ± 0.013	0.62 ± 0.019	0.62 ± 0.016	0.58 ± 0.021	0.62 ± 0.015	0.63 ± 0.017	0.62 ± 0.011	0.65 ± 0.013	0.64 ± 0.015
10	0.56 ± 0.021	0.6 ± 0.012	0.59 ± 0.012	0.59 ± 0.019	0.63 ± 0.019	0.59 ± 0.017	0.59 ± 0.018	0.62 ± 0.012	0.62 ± 0.009	0.61 ± 0.013	0.65 ± 0.012	0.63 ± 0.018
20	0.57 ± 0.032	0.6 ± 0.018	0.6 ± 0.014	0.6 ± 0.019	0.61± 0.011	0.6 ± 0.016	0.59 ± 0.021	0.61 ± 0.015	0.63 ± 0.012	0.62 ± 0.014	0.66 ± 0.012	0.64 ± 0.015
30	0.58 ± 0.028	0.61 ± 0.01	0.6 ± 0.012	0.61 ± 0.016	0.61 ± 0.022	0.6 ± 0.018	0.59 ± 0.021	0.62 ± 0.014	0.64 ± 0.013	0.62 ± 0.013	0.67 ± 0.011	0.65 ± 0.015
40	0.57 ± 0.021	0.61 ± 0.014	0.6 ± 0.017	0.59 ± 0.019	0.63 ± 0.016	0.6 ± 0.01	0.59 ± 0.019	0.61 ± 0.012	0.64 ± 0.009	0.62 ± 0.014	0.68 ± 0.015	0.65 ± 0.017
50	0.56 ± 0.021	0.62 ± 0.013	0.59 ± 0.012	0.6 ± 0.019	0.63 ± 0.018	0.6 ± 0.016	0.58 ± 0.018	0.62 ± 0.014	0.64 ± 0.013	0.63 ± 0.016	0.68 ± 0.021	0.65 ± 0.009
60	0.57 ± 0.015	0.61 ± 0.016	0.6 ± 0.016	0.6 ± 0.019	0.63 ± 0.023	0.6 ± 0.014	0.58 ± 0.022	0.62 ± 0.015	0.64 ± 0.013	0.62 ± 0.016	0.68 ± 0.016	0.65 ± 0.02
70	0.58 ± 0.017	0.62 ± 0.018	0.62 ± 0.018	0.61 ± 0.02	0.62 ± 0.022	0.62 ± 0.012	0.6 ± 0.016	0.63 ± 0.019	0.64 ± 0.01	0.63 ± 0.014	0.68 ± 0.014	0.65 ± 0.014
80	0.59 ± 0.021	0.62 ± 0.009	0.61 ± 0.009	0.61 ± 0.017	0.62 ± 0.02	0.62 ± 0.017	0.59 ± 0.024	0.62 ± 0.019	0.65 ± 0.013	0.64 ± 0.013	0.68 ± 0.013	0.66 ± 0.012
90	0.58 ± 0.017	0.62 ± 0.012	0.6 ± 0.012	0.6 ± 0.02	0.61 ± 0.02	0.62 ± 0.015	0.6 ± 0.016	0.62 ± 0.018	0.64 ± 0.01	0.63 ± 0.015	0.68 ± 0.012	0.65 ± 0.013
100	0.59 ± 0.02	0.62 ± 0.019	0.61 ± 0.019	0.61 ± 0.014	0.62 ± 0.021	0.62 ± 0.013	0.61 ± 0.021	0.63 ± 0.016	0.66 ± 0.017	0.64 ± 0.024	0.68 ± 0.018	0.65 ± 0.01

**Table 6:** Mean accuracy (mean *± SD* over 20 runs; *n* = 20) on the held-out test part of the second dataset. The sign ± denotes the run-to-run standard deviation; the standard error of the mean is *SD*/√20.

Nominal Expansion, %	VGG19	ResNet34	ResNet50	ResNet101	Xception	Inception	DenseNet161	DenseNet201	VIT	DeIT	BeIT	ConvNeXT
0	0.64 ± 0.024	0.78 ± 0.014	0.75 ± 0.013	0.76 ± 0.018	0.75 ± 0.01	0.75 ± 0.013	0.72 ± 0.019	0.72 ± 0.023	0.77 ± 0.016	0.78 ± 0.013	0.78 ± 0.012	0.66 ± 0.021
10	0.63 ± 0.029	0.79 ± 0.012	0.75 ± 0.014	0.75 ± 0.02	0.76 ± 0.011	0.75 ± 0.02	0.72 ± 0.025	0.72 ± 0.016	0.79 ± 0.01	0.79 ± 0.012	0.79 ± 0.01	0.66 ± 0.022
20	0.62 ± 0.036	0.78 ± 0.016	0.74 ± 0.02	0.76 ± 0.017	0.74 ± 0.012	0.76 ± 0.01	0.73 ± 0.026	0.72 ± 0.014	0.79 ± 0.014	0.78 ± 0.012	0.79 ± 0.016	0.67 ± 0.017
30	0.62 ± 0.022	0.77 ± 0.012	0.76 ± 0.017	0.77 ± 0.017	0.74 ± 0.011	0.75 ± 0.015	0.71 ± 0.024	0.73 ± 0.022	0.78 ± 0.012	0.78 ± 0.012	0.79 ± 0.011	0.67 ± 0.019
40	0.63 ± 0.027	0.78 ± 0.011	0.73 ± 0.023	0.74 ± 0.021	0.75 ± 0.012	0.74 ± 0.015	0.7 ± 0.019	0.7 ± 0.028	0.78 ± 0.01	0.78 ± 0.015	0.79 ± 0.015	0.67 ± 0.019
50	0.63 ± 0.027	0.77 ± 0.014	0.73 ± 0.017	0.73 ± 0.022	0.74 ± 0.013	0.75 ± 0.016	0.71 ± 0.027	0.7 ± 0.026	0.77 ± 0.016	0.78 ± 0.014	0.78 ± 0.012	0.67 ± 0.018
60	0.62 ± 0.027	0.75 ± 0.015	0.73 ± 0.015	0.74 ± 0.019	0.75 ± 0.015	0.73 ± 0.014	0.7 ± 0.023	0.7 ± 0.023	0.77 ± 0.014	0.78 ± 0.014	0.78 ± 0.014	0.68 ± 0.019
70	0.6 ± 0.023	0.76 ± 0.013	0.71 ± 0.028	0.73 ± 0.021	0.73 ± 0.011	0.74 ± 0.02	0.69 ± 0.014	0.69 ± 0.014	0.77 ± 0.014	0.77 ± 0.014	0.78 ± 0.014	0.68 ± 0.017
80	0.6 ± 0.034	0.75 ± 0.016	0.72 ± 0.024	0.74 ± 0.017	0.72 ± 0.013	0.74 ± 0.011	0.68 ± 0.026	0.68 ± 0.026	0.77 ± 0.013	0.77 ± 0.014	0.78 ± 0.013	0.68 ± 0.018
90	0.61 ± 0.025	0.76 ± 0.018	0.73 ± 0.029	0.72 ± 0.025	0.72 ± 0.017	0.73 ± 0.016	0.69 ± 0.021	0.69 ± 0.021	0.77 ± 0.02	0.76 ± 0.012	0.77 ± 0.015	0.68 ± 0.02
100	0.59 ± 0.04	0.74 ± 0.015	0.71 ± 0.024	0.71 ± 0.023	0.71 ± 0.013	0.74 ± 0.015	0.68 ± 0.032	0.68 ± 0.032	0.77 ± 0.011	0.76 ± 0.013	0.77 ± 0.011	0.68 ± 0.018

**Table 7:** Parameters of the ABVR-inspired response curve (12), fitted by weighted least squares to the eleven nominal expansion levels in Table 5 and Table 6 (weights 1/*SE*^2^; nominally 8 residual degrees of freedom). *R*^2^ is calculated using the unweighted residual criterion. The fitted maximizer ${\hat{r}}^{*}$ is expressed as a percentage of the nominal training-set expansion, not as the fraction of transformed images. Parameters and maximizers are in-sample estimates.

Model	Main Dataset				Replication Dataset			
	$\hat{v}$	$\hat{h}$	${\hat{r}}^{*}$, %	*R* ^2^	$\hat{v}$	$\hat{h}$	${\hat{r}}^{*}$, %	*R* ^2^
VGG19	0.0632	0.000	100	0.641	0.000	0.145	0	0.72
ResNet34	0.036	0.000	100	0.60	0.021	0.204	5	0.80
ResNet50	0.028	0.000	100	0.26	0.017	0.193	5	0.67
ResNet101	0.046	0.021	100	0.44	0.051	0.265	11	0.73
Xception	0.019	0.026	56	−0.03	0.043	0.231	10	0.76
Inception	0.035	0.000	100	0.20	0.011	0.104	5	0.51
DenseNet161	0.035	0.000	100	0.32	0.000	0.171	0	0.85
DenseNet201	0.015	0.000	100	0.18	0.033	0.242	7	0.84
ViT	0.065	0.021	100	0.62	0.024	0.113	12	0.46
DeiT	0.040	0.000	100	0.62	0.052	0.189	16	0.86
BeIT	0.133	0.118	100	0.91	0.079	0.209	23	0.87
ConvNeXT	0.064	0.057	100	0.61	0.048	0.006	100	0.89

## Data Availability

The CEAP image dataset is available on Harvard Dataverse at https://doi.org/10.7910/DVN/ZQMHUN [13]. The dermatological source dataset is identified in [14], version 1 (CC0: public domain; accessed on 20 June 2026). The experiment comprised 5280 training runs.

## Supplementary Materials

The following supporting information can be downloaded at https://www.mdpi.com/article/10.3390/jimaging12100502/s1. Sections S1–S4 provide paired class-wise performance plots, run-to-run dispersion panels and accuracy box plots.
