# Supplementary material for: Robustness of AI Models to Synthetic Data in Color Image Classification: A Comparative Analysis
Source: J Imaging. 2026 Oct 10;12(10):502. doi: 10.3390/jimaging12100502 (PMC13706283; doi:10.3390/jimaging12100502)
Supplement: Supplementary file 1 [file jimaging-12-00502-s001.zip › jimaging-4557815-Supplementary Materials.pdf]

Journal of Imaging

Supplementary Materials

Robustness of AI Models to Synthetic Data in Color Image Classification: A Comparative Analysis

Marina Barulina [1,2,*], Sergey Okunkov [1,2] and Ivan Ulitin [1,2]

[1] Institute of Physical and Mathematical Sciences, Perm State University, 614068 Perm, Russia; sergeiokunkov@yandex.ru (S.O.); ivulitin@psu.ru (I.U.)
[2] Institute of Precision Mechanics and Control, Russian Academy of Sciences, 410028 Saratov, Russia
* Correspondence: mab@psu.ru

This supplement reorganizes the submitted graphical results. Paired panels are shown once.

Terminology: historical plot legends may say “synthetic data”, “validation dataset” or α. In the revised manuscript these mean nominal mixed-addition expansion r and the replication dataset, not the transformed-image fraction or an internal validation split.

Contents and evidence status

- S1: 12 Architecture-Matched Pairs of Class-Wise Precision/Recall Panels.
- S2: 10 Paired Legacy Dispersion Panels; One Metric-Label Ambiguity is Explicitly Flagged.
- S3: 11 Matched-Level Pairs of Accuracy Box Plots.
- S4: 2 Architecture-Matched Pairs of Illustrative Box Plots.

 https://doi.org/10.3390/jimaging12100502

S1. 12 Architecture-Matched Pairs of Class-Wise Precision/Recall Panels.

(a) Main dataset

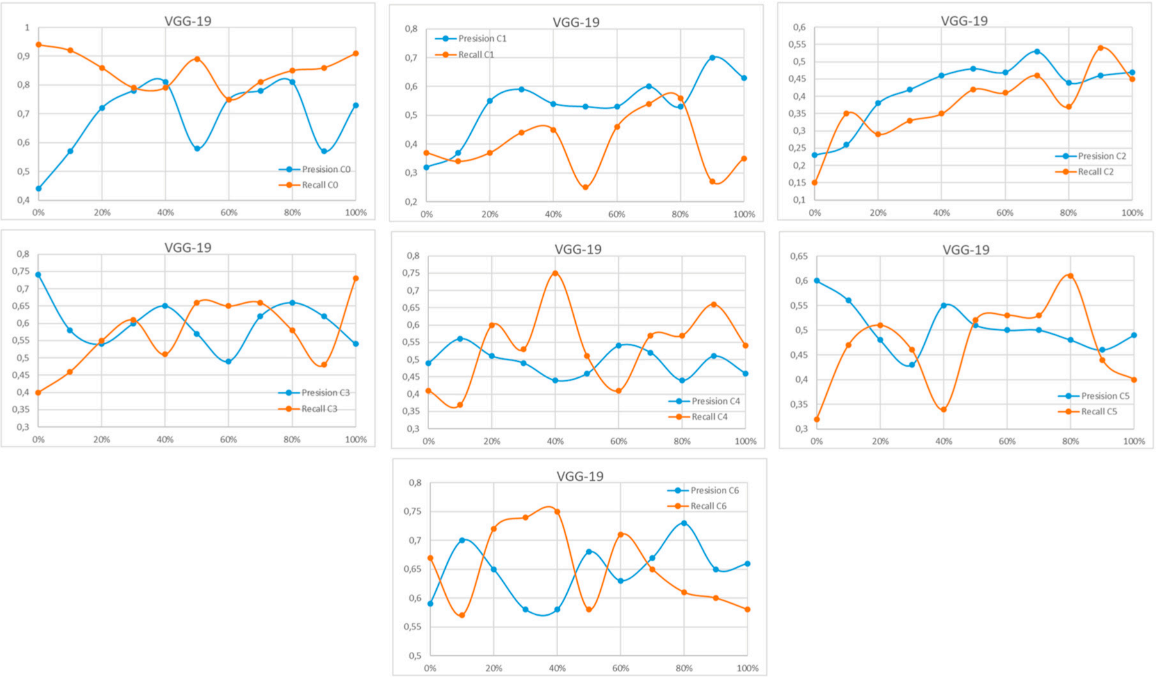

(b) Replication dataset

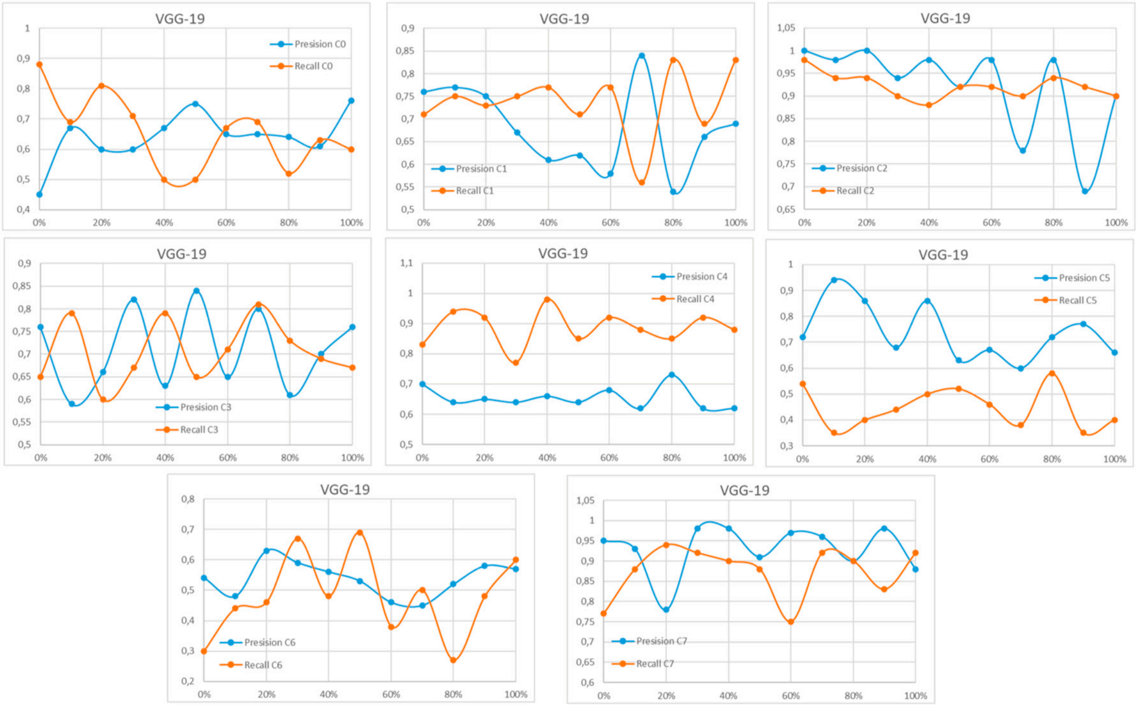

Figure S1. VGG19: class-wise performance.

(a) Main dataset

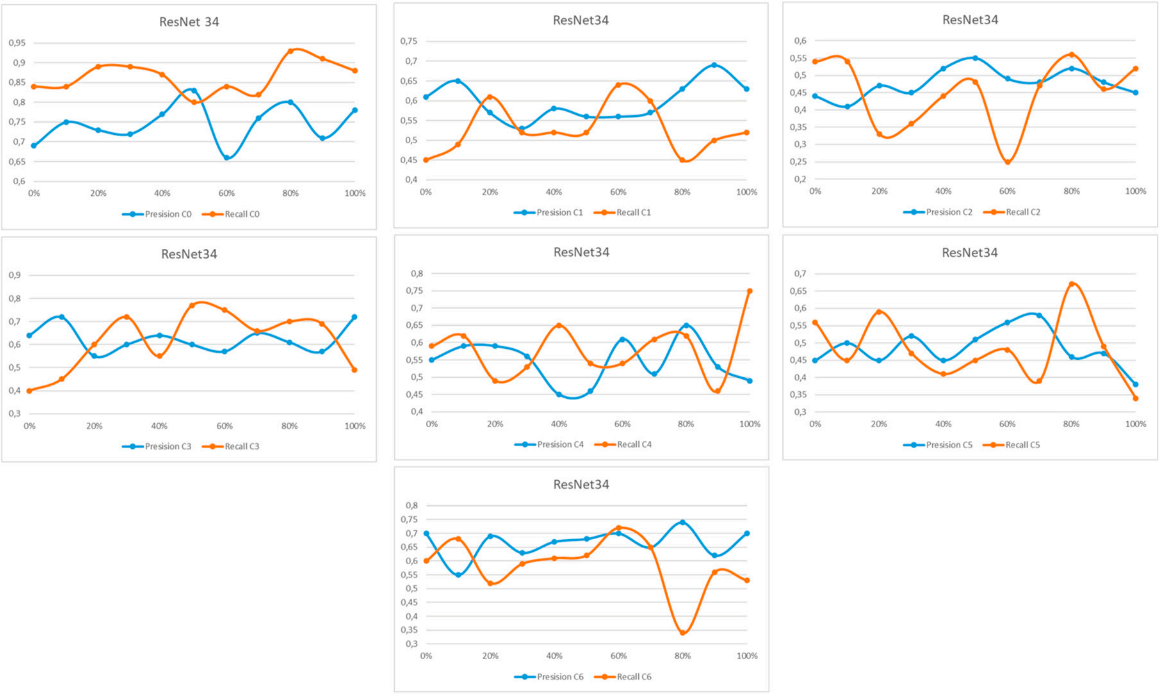

(b) Replication dataset

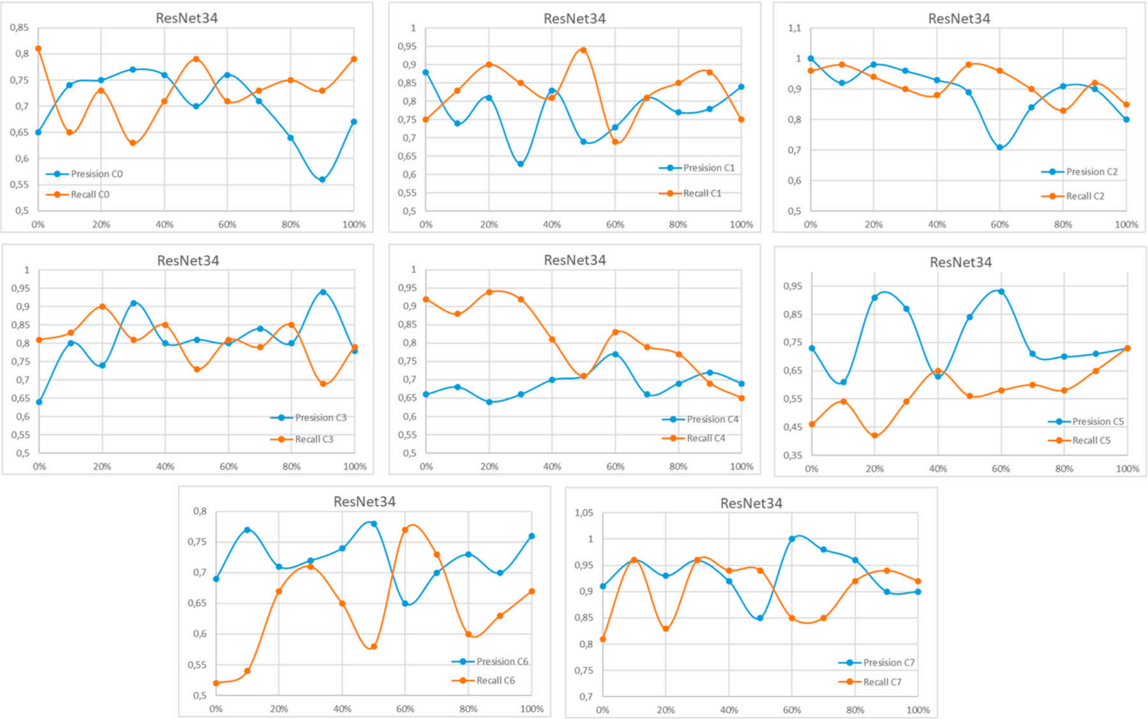

Figure S2. ResNet34: class-wise performance.

(a) Main dataset

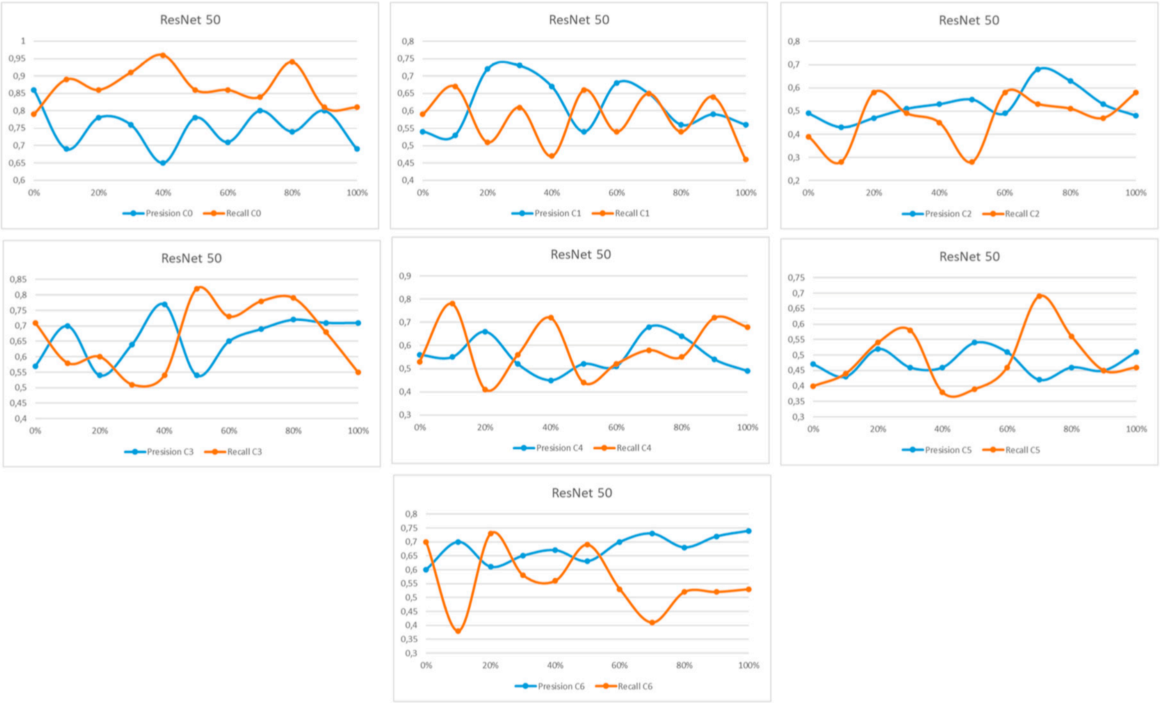

(b) Replication dataset

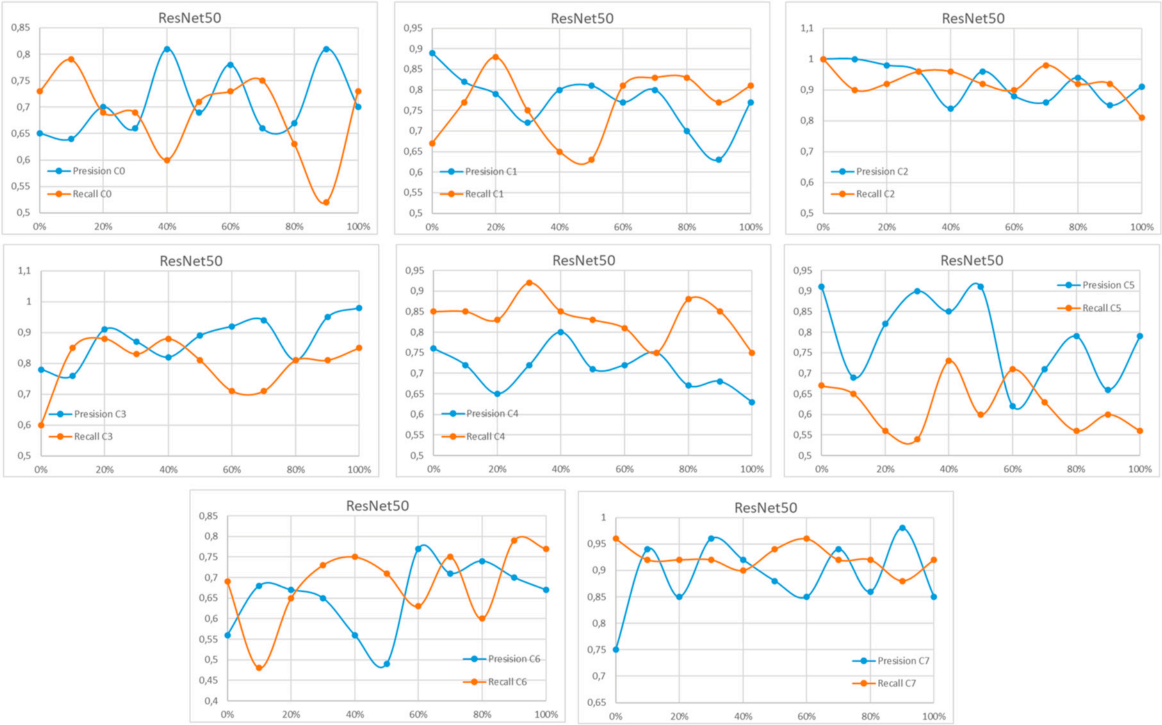

Figure S3. ResNet50: class-wise performance.

(a) Main dataset

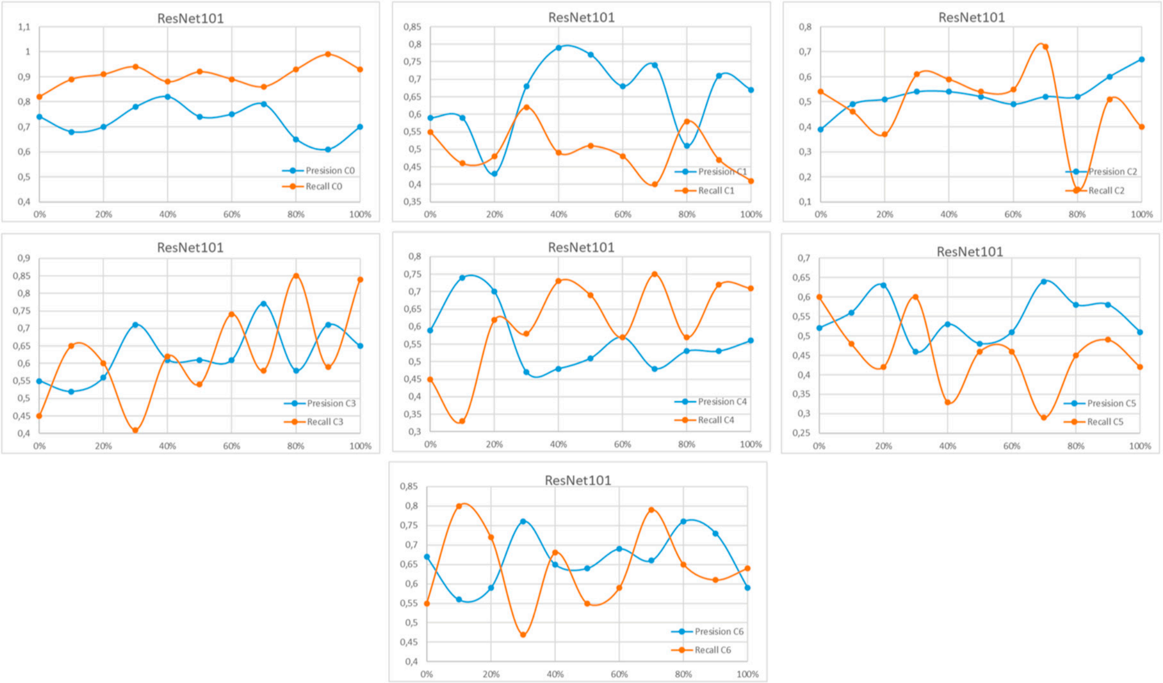

(b) Replication dataset

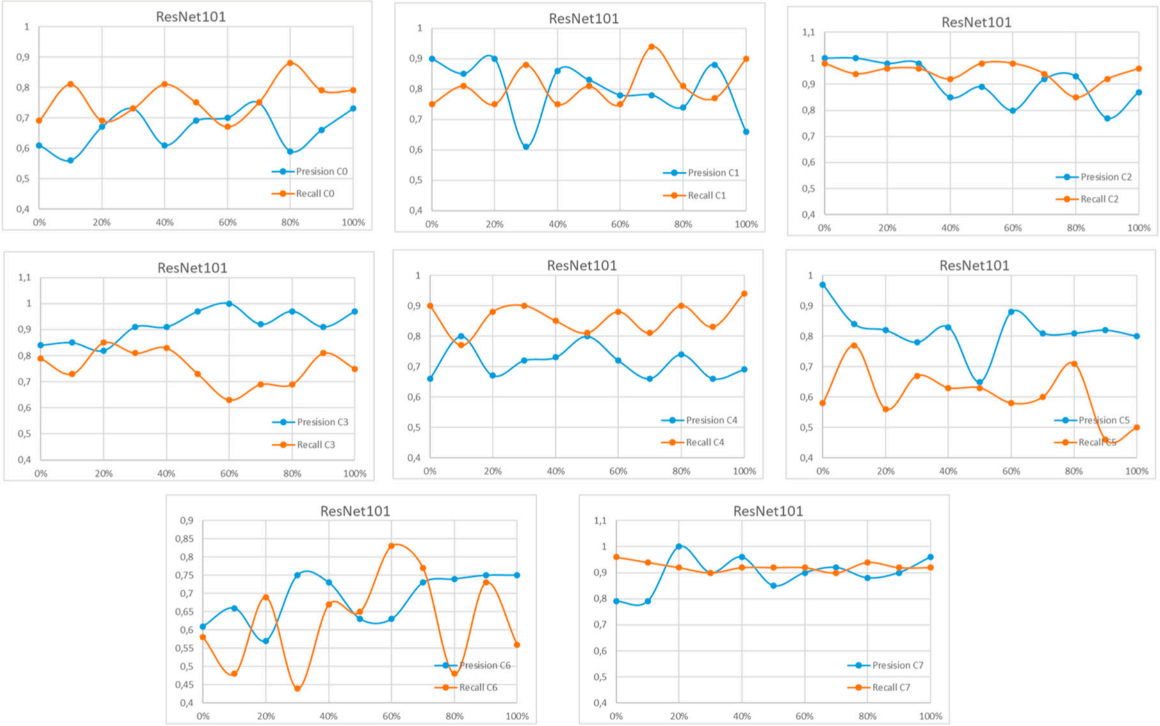

Figure S4. ResNet101: class-wise performance.

(a) Main dataset

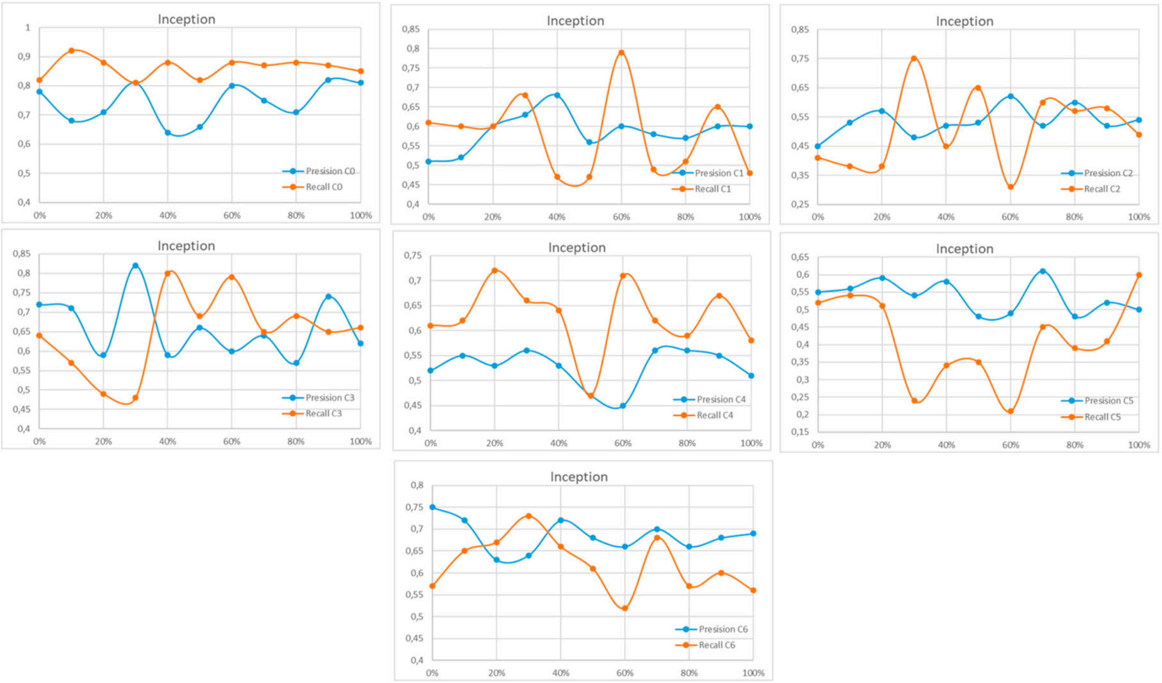

(b) Replication dataset

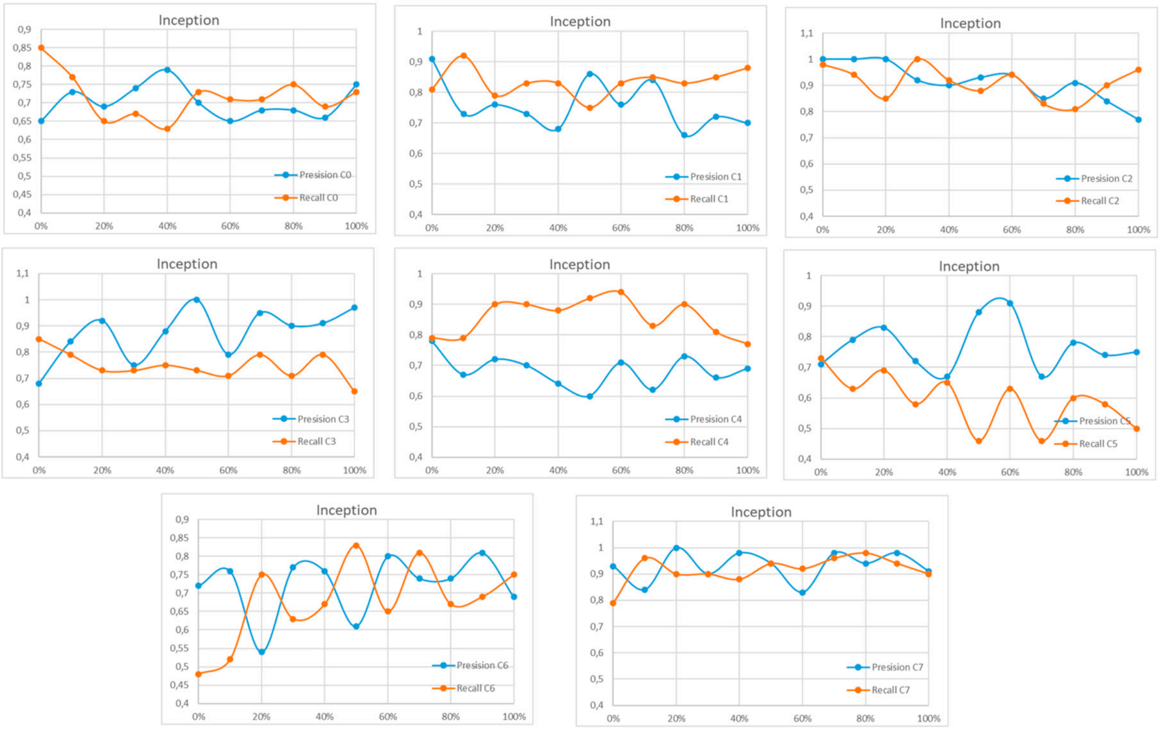

Figure S5. Inception: class-wise performance

https://doi.org/10.3390/jimaging12100502

(a) Main dataset

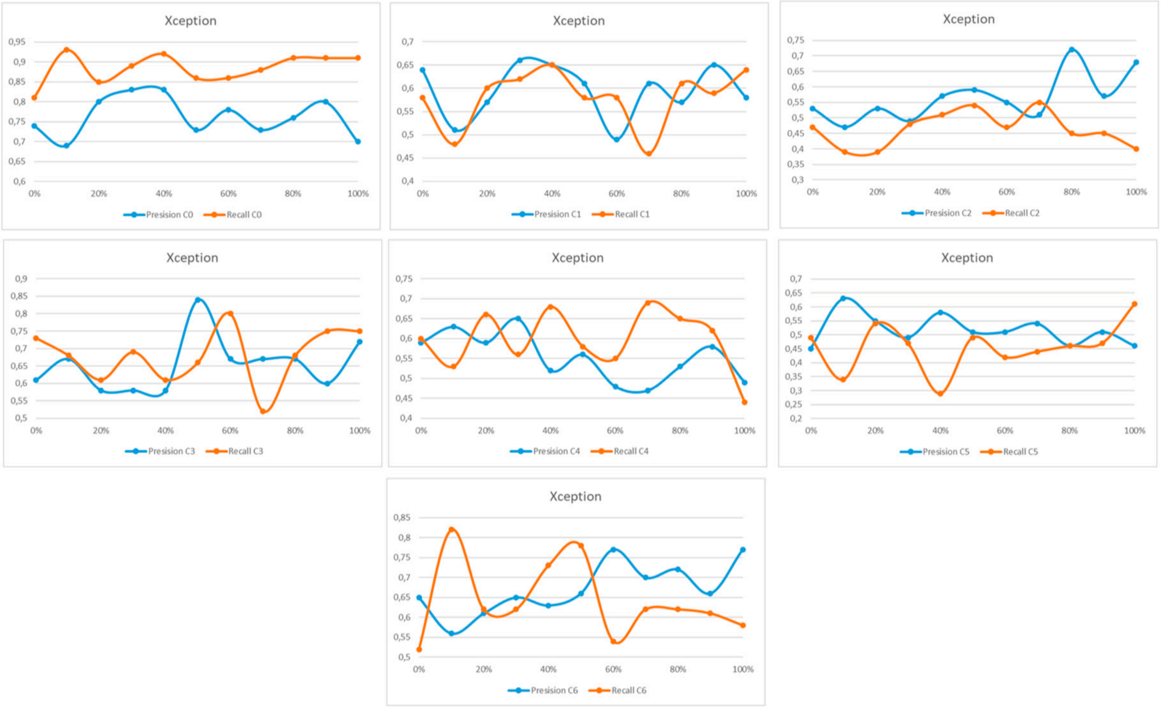

(b) Replication dataset

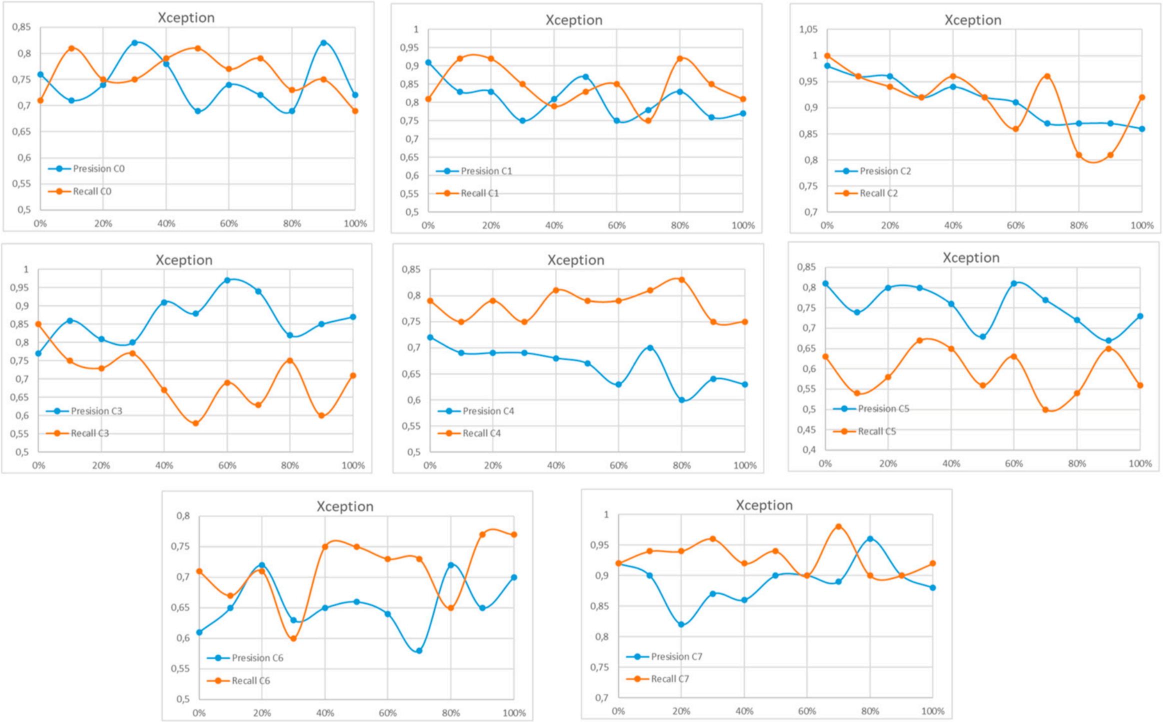

Figure S6. Xception: class-wise performance

(a) Main dataset

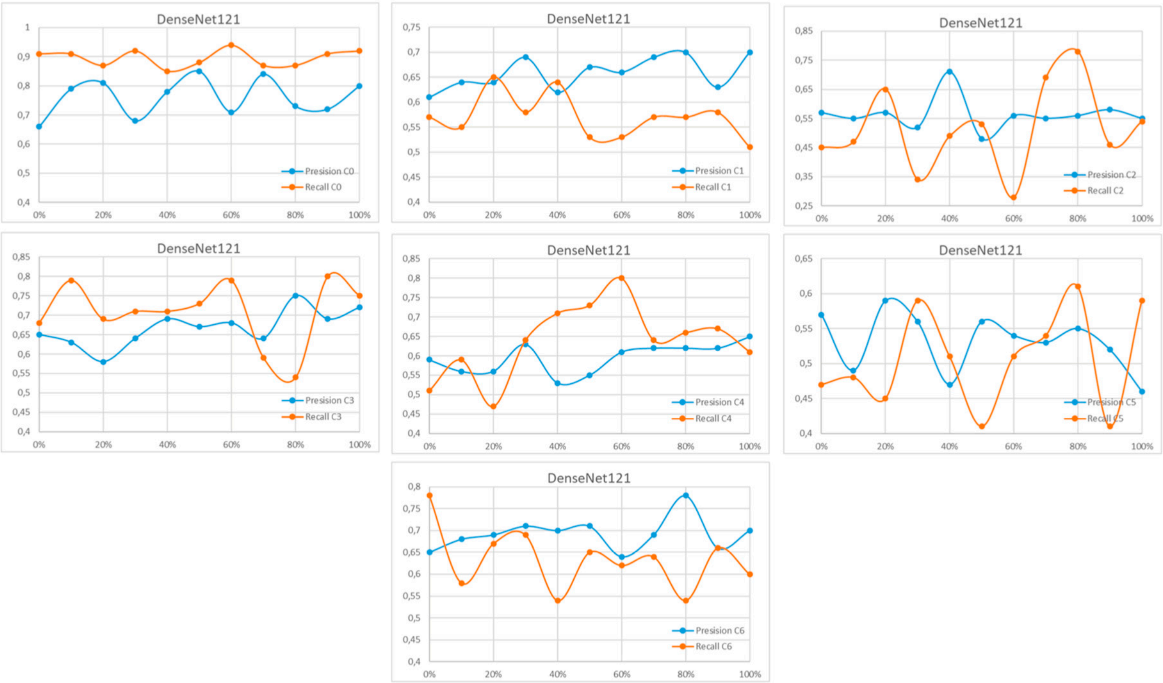

(b) Replication dataset

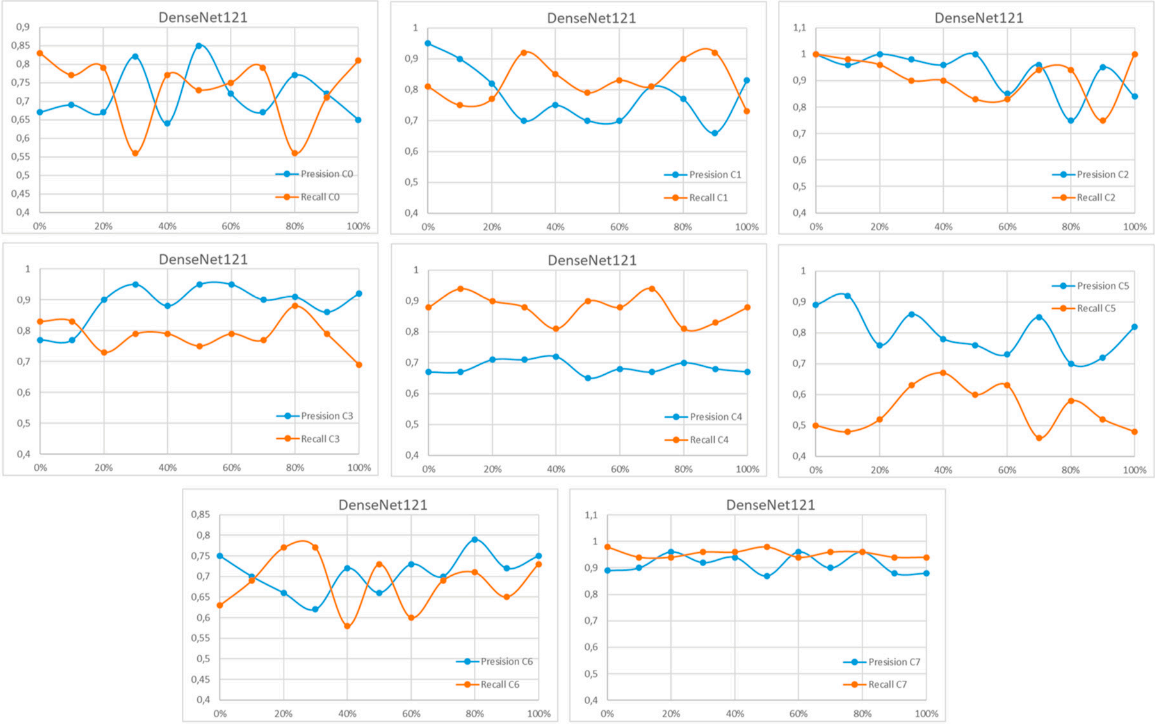

Figure S7. DenseNet161: class-wise performance

https://doi.org/10.3390/jimaging12100502

(a) Main dataset

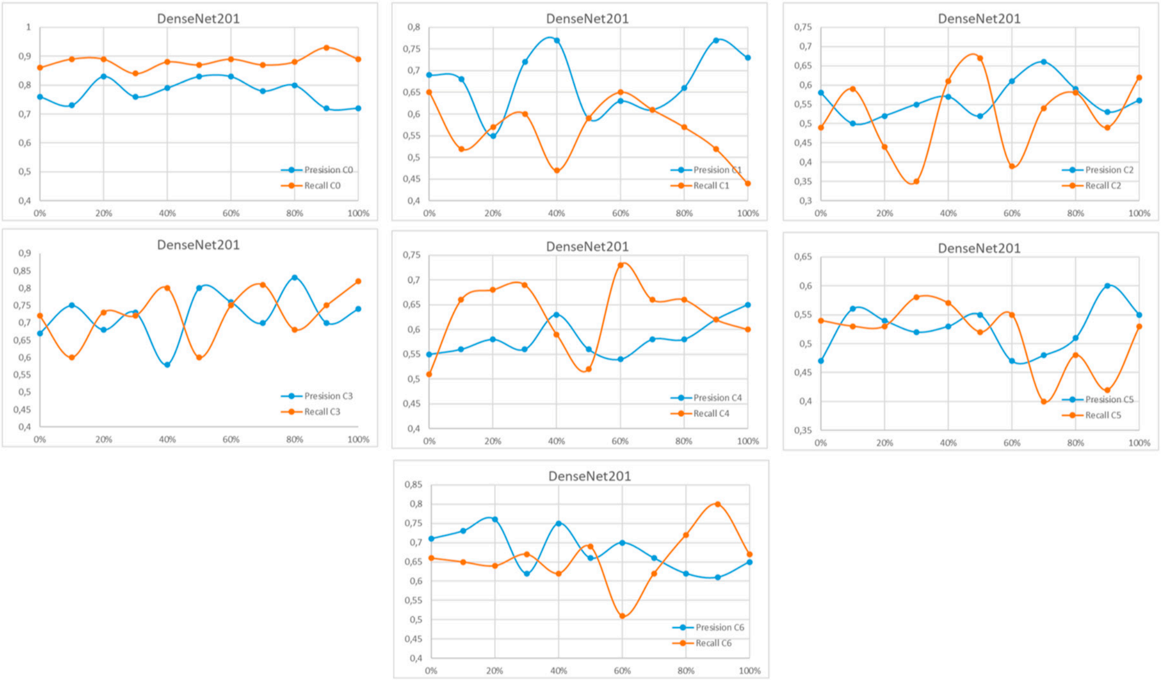

(b) Replication dataset

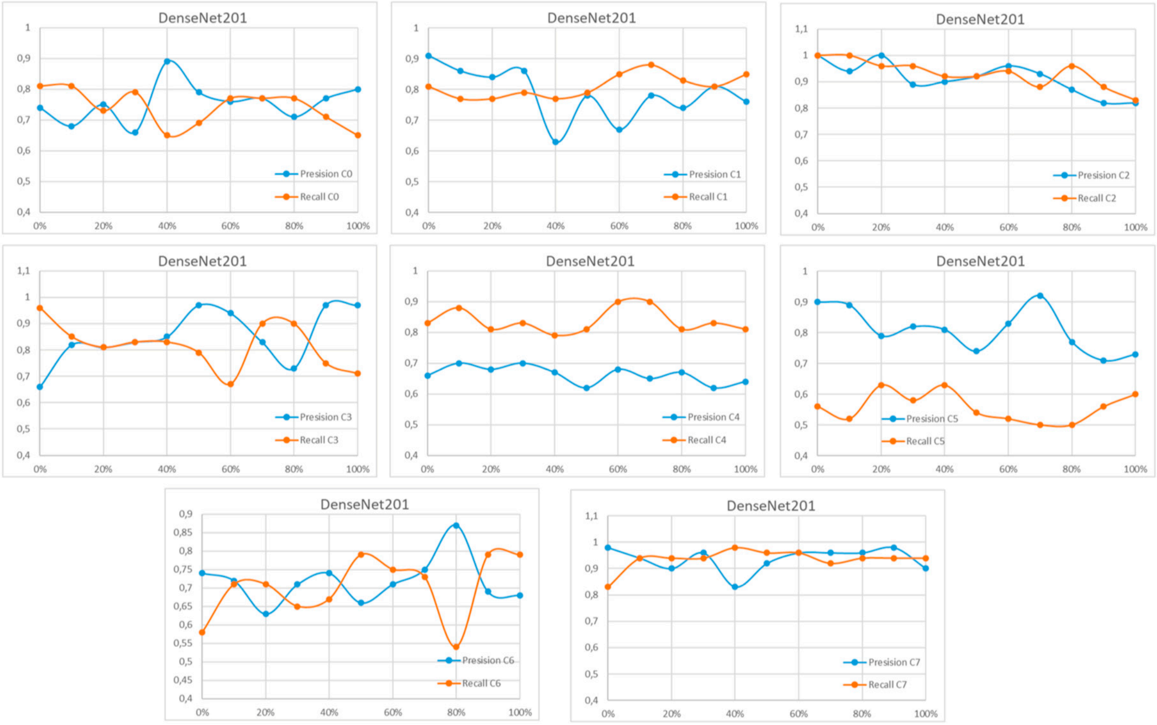

Figure S8. DenseNet201: class-wise performance

https://doi.org/10.3390/jimaging12100502

(a) Main dataset

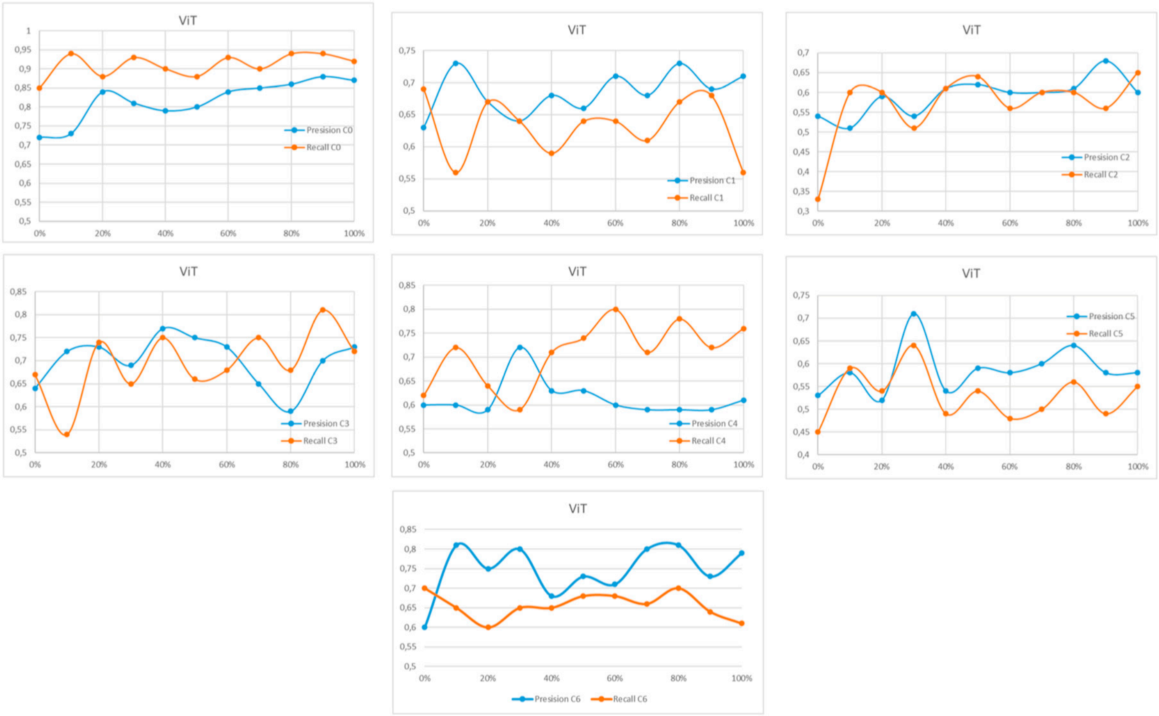

(b) Replication dataset

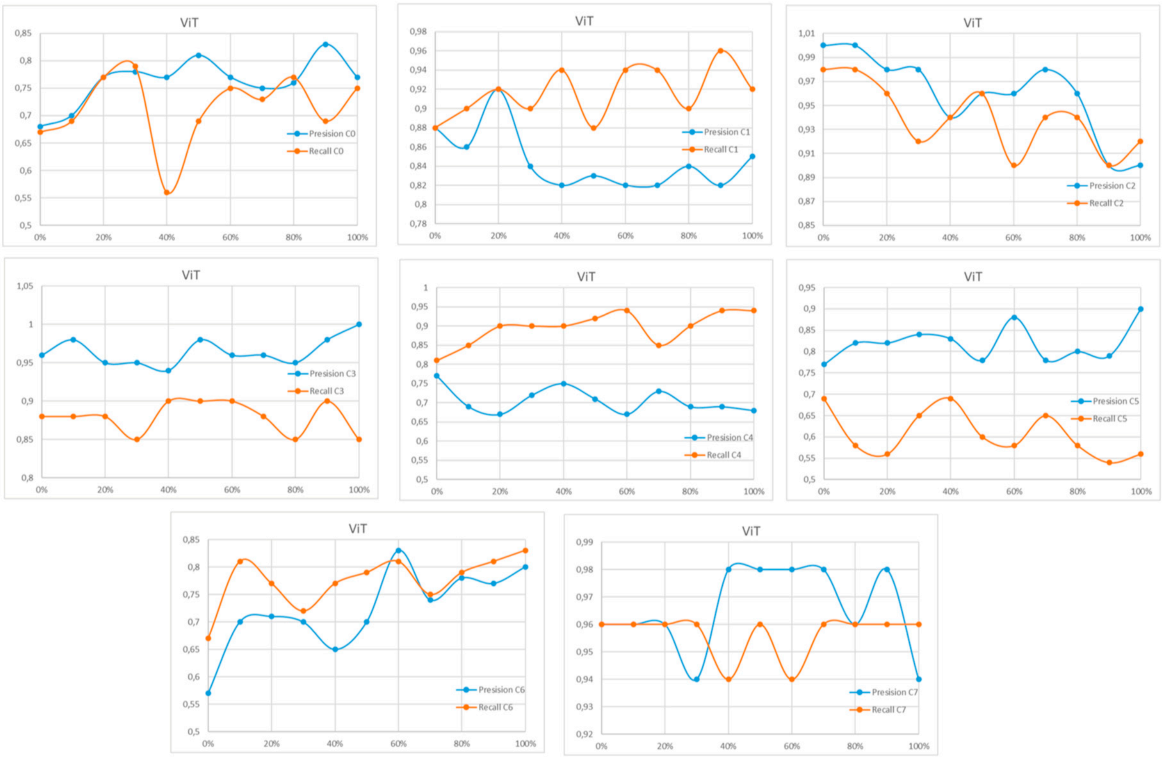

Figure S9. ViT: class-wise performance

(a) Main dataset

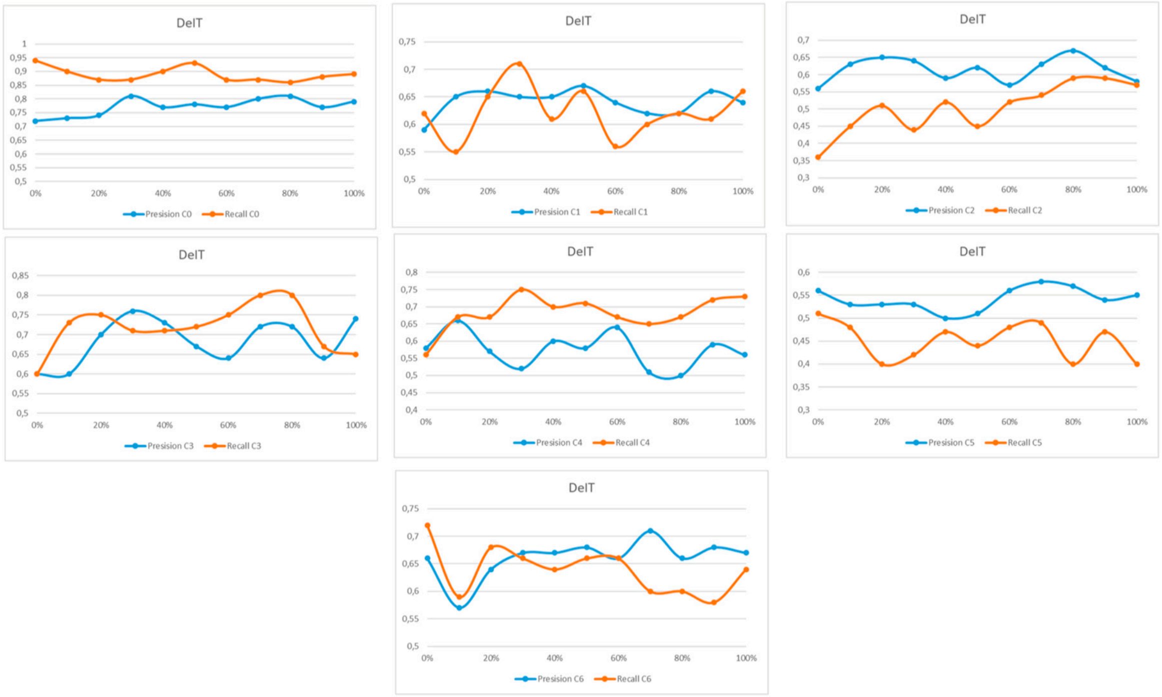

(b) Replication dataset

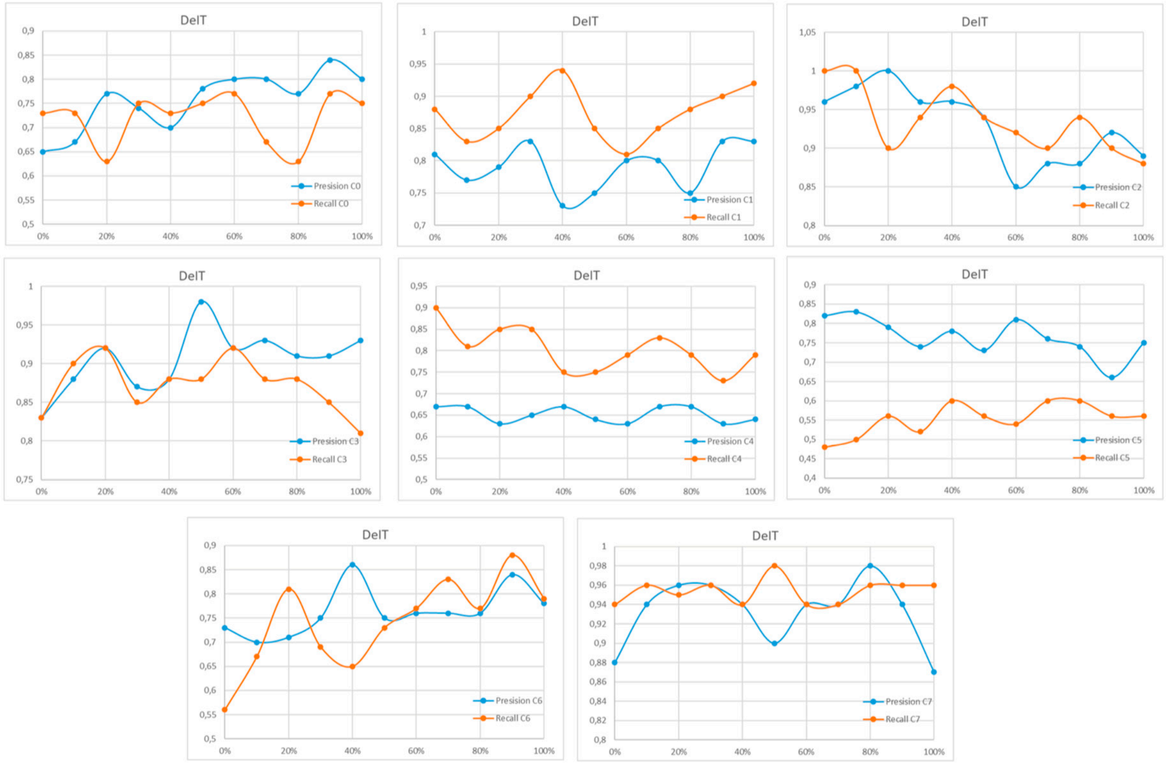

Figure S10. DeiT: class-wise performance

(a) Main dataset

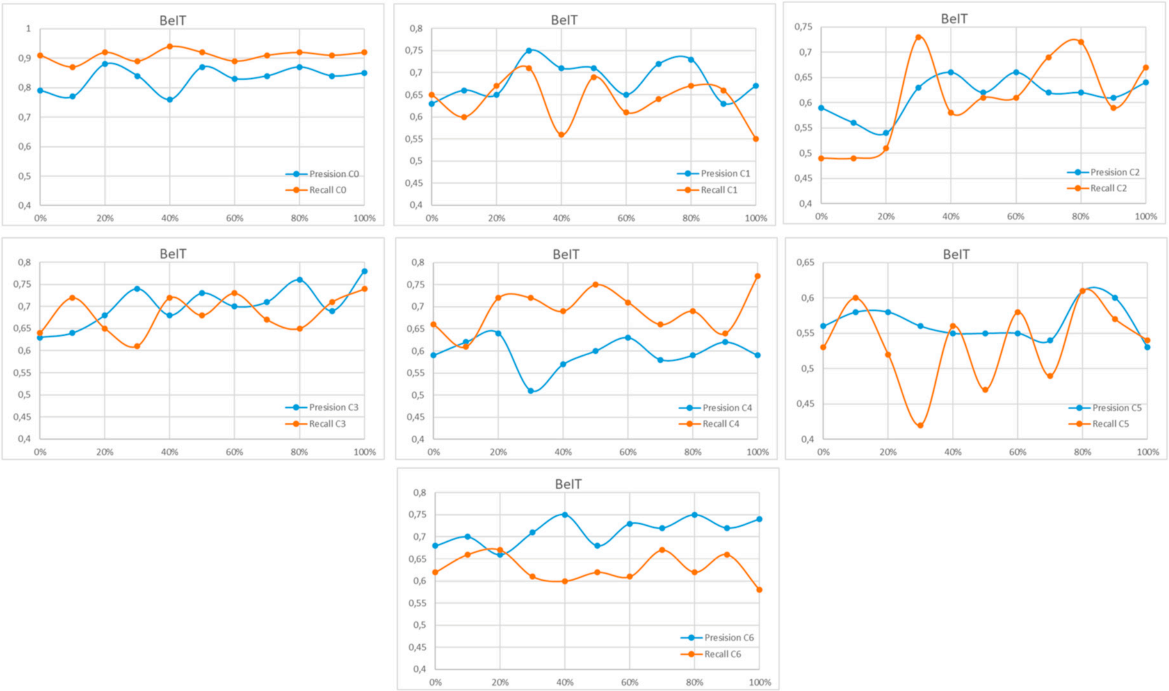

(b) Replication dataset

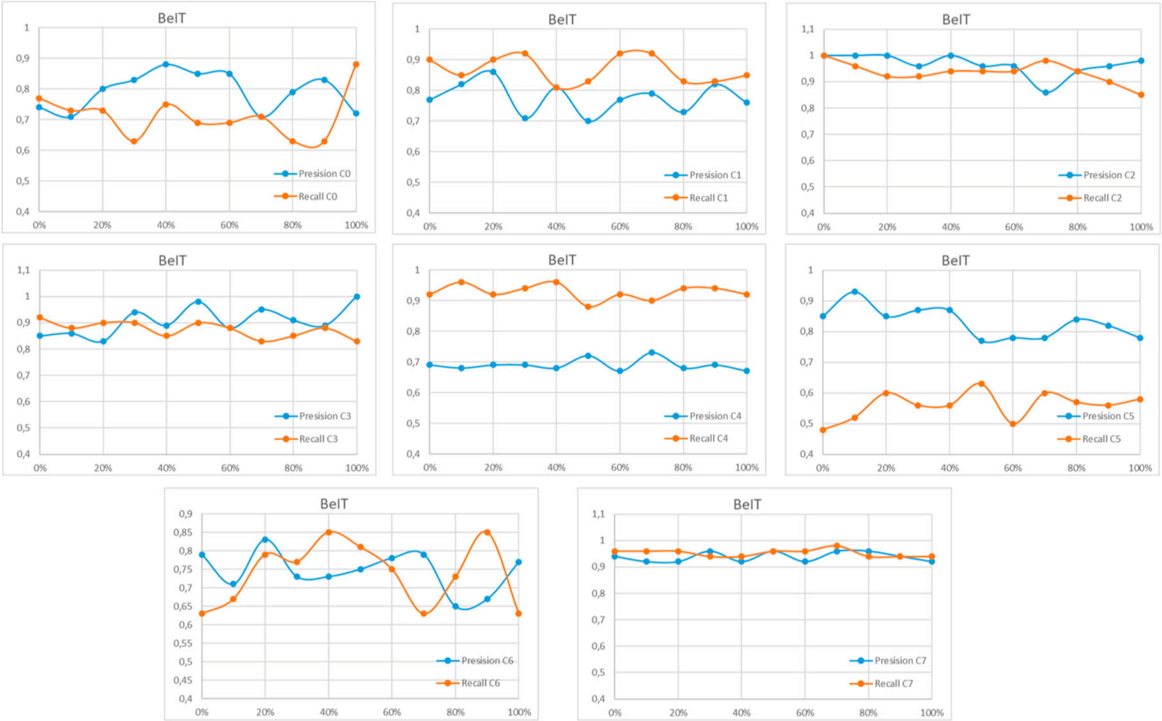

Figure S11. BeIT: class-wise performance

https://doi.org/10.3390/jimaging12100502

(a) Main dataset

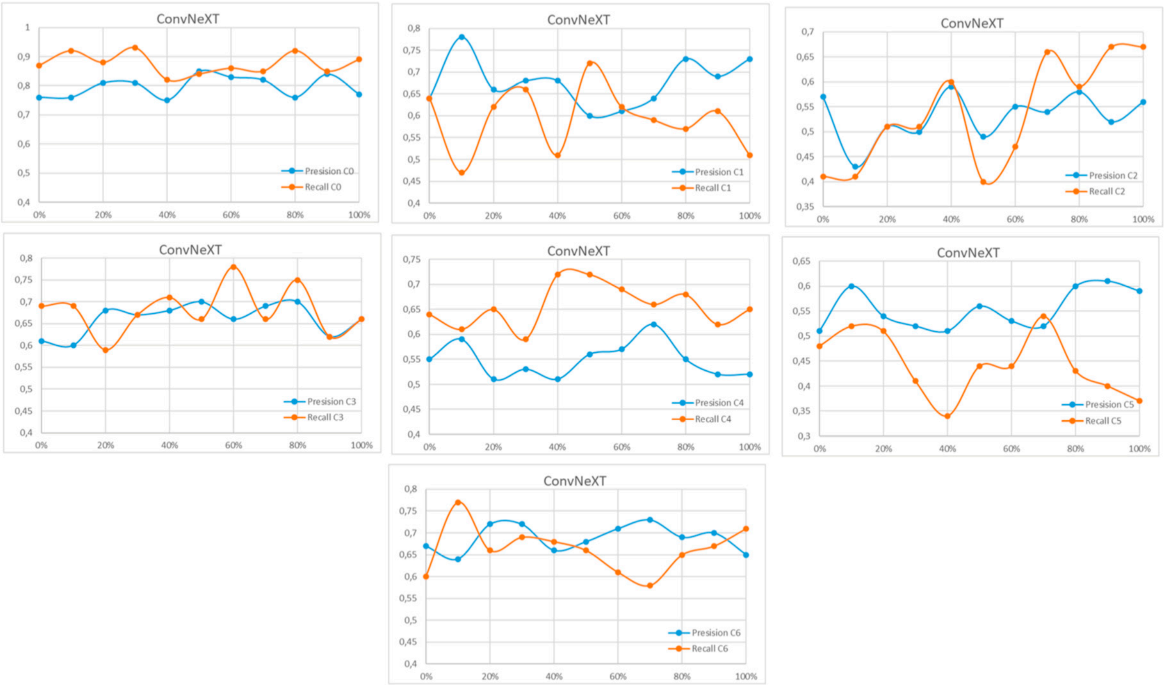

(b) Replication dataset

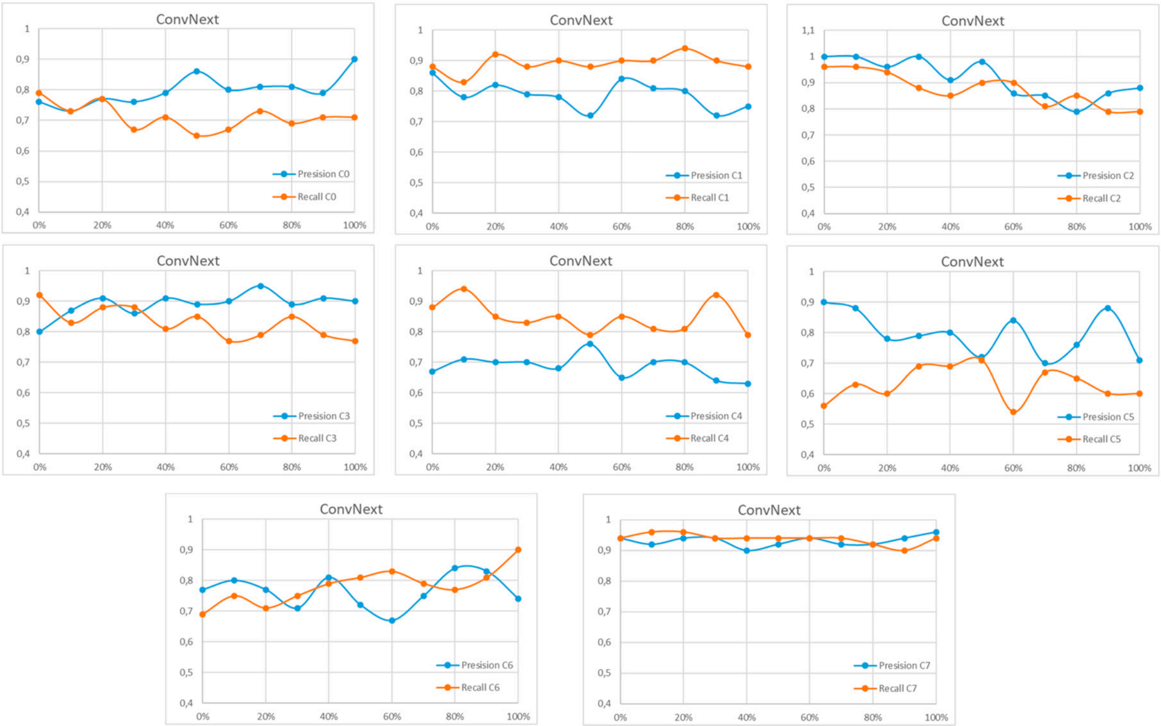

Figure S12. ConvNeXt: class-wise performance

S2: 10 Paired Legacy Dispersion Panels; One Metric-Label Mmbiguity is Explicitly Flagged.

Original submitted dispersion panels; paired by corresponding panel role, not by disease equivalence.

(a) Main dataset

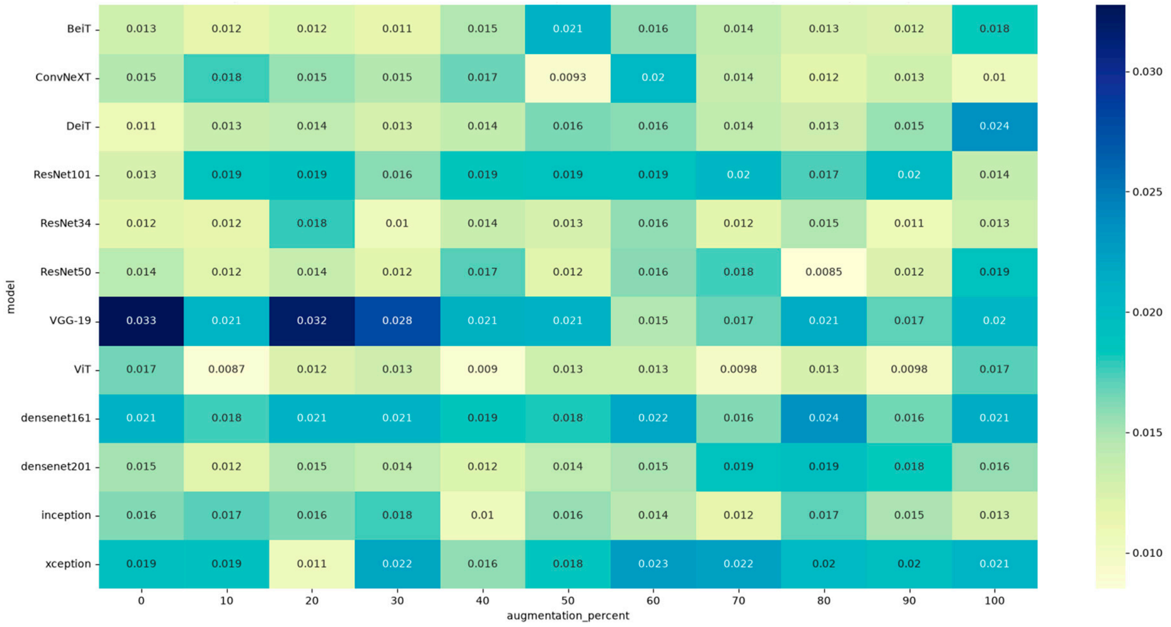

(b) Replication dataset

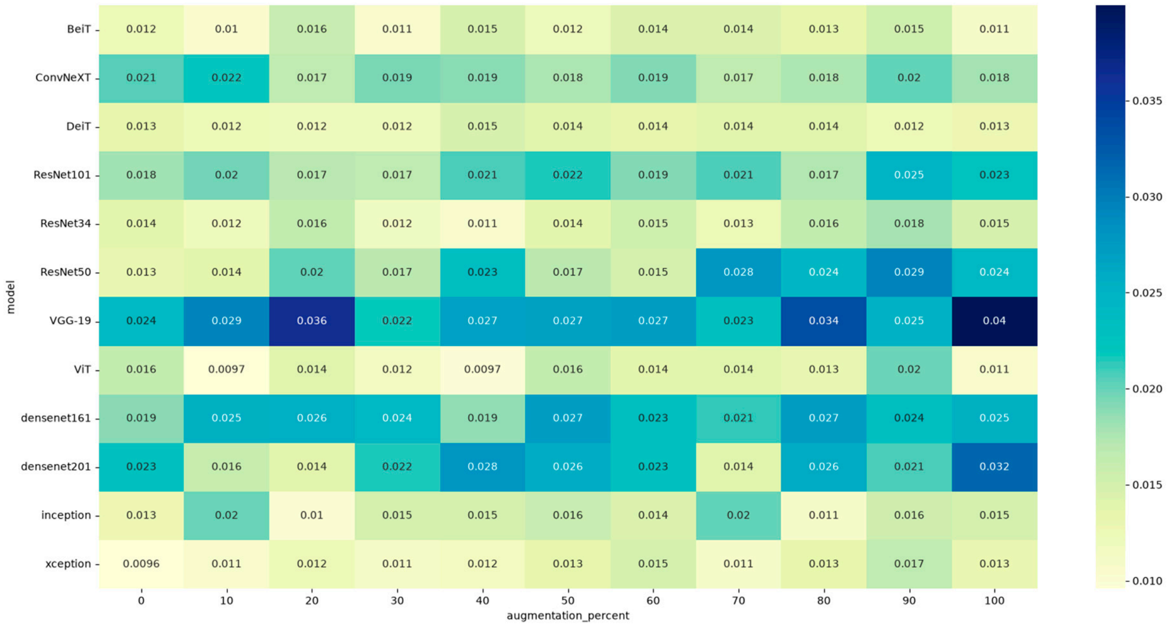

Figure S13. Accuracy SD

https://doi.org/10.3390/jimaging12100502

(a) Main dataset

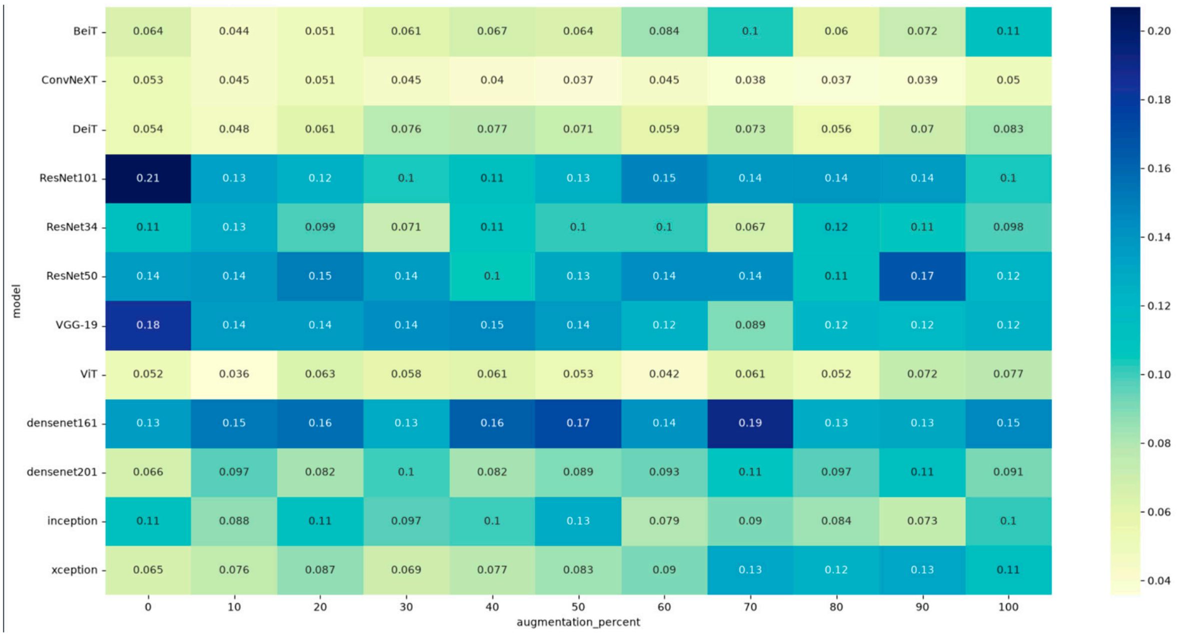

(b) Replication dataset

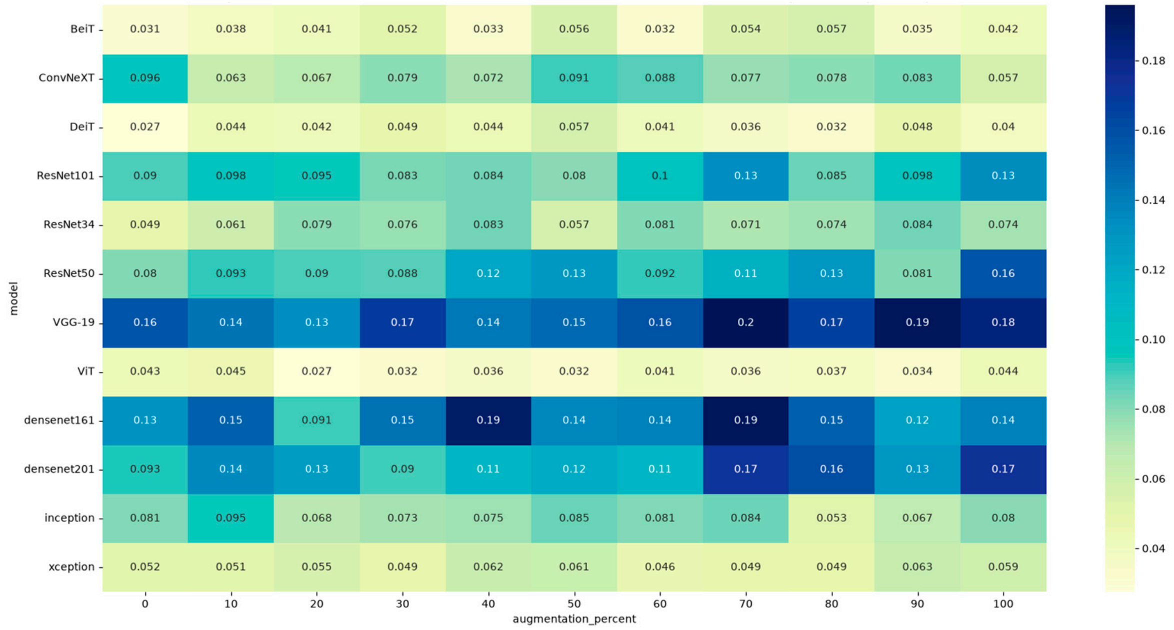

Figure S14. Originally underrepresented class recall: C5 / C3

Editorial caveat: the submitted caption for replication panel B13 repeats "recall". Its metric cannot be identified reliably from the supplied image alone; precision is not silently inferred.

(a) Main dataset

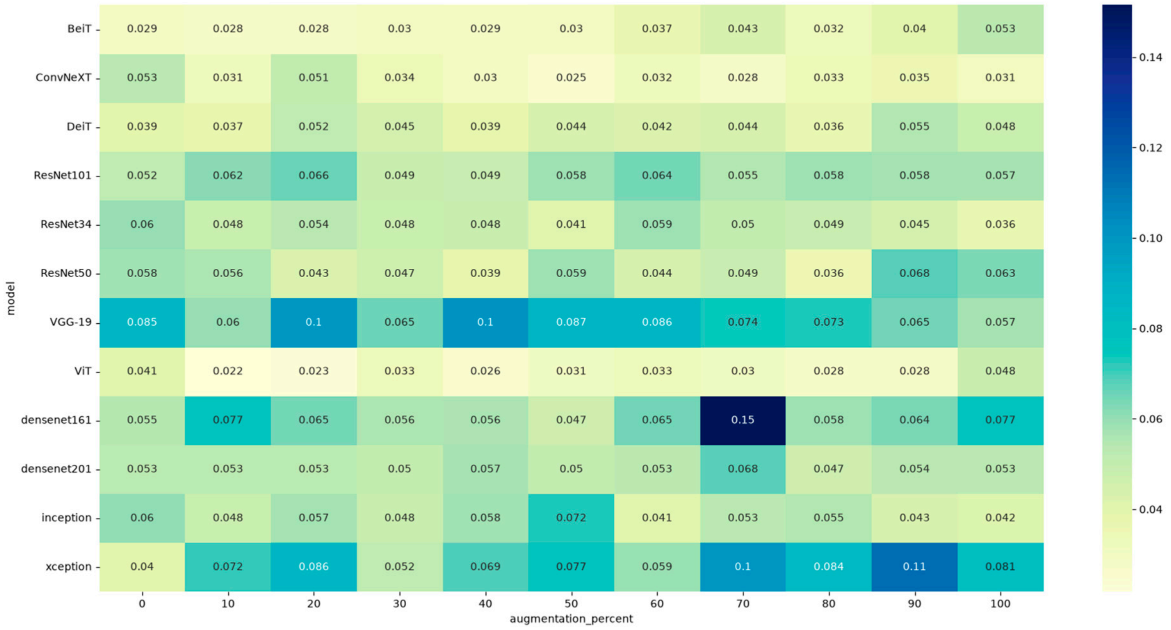

(b) Replication dataset

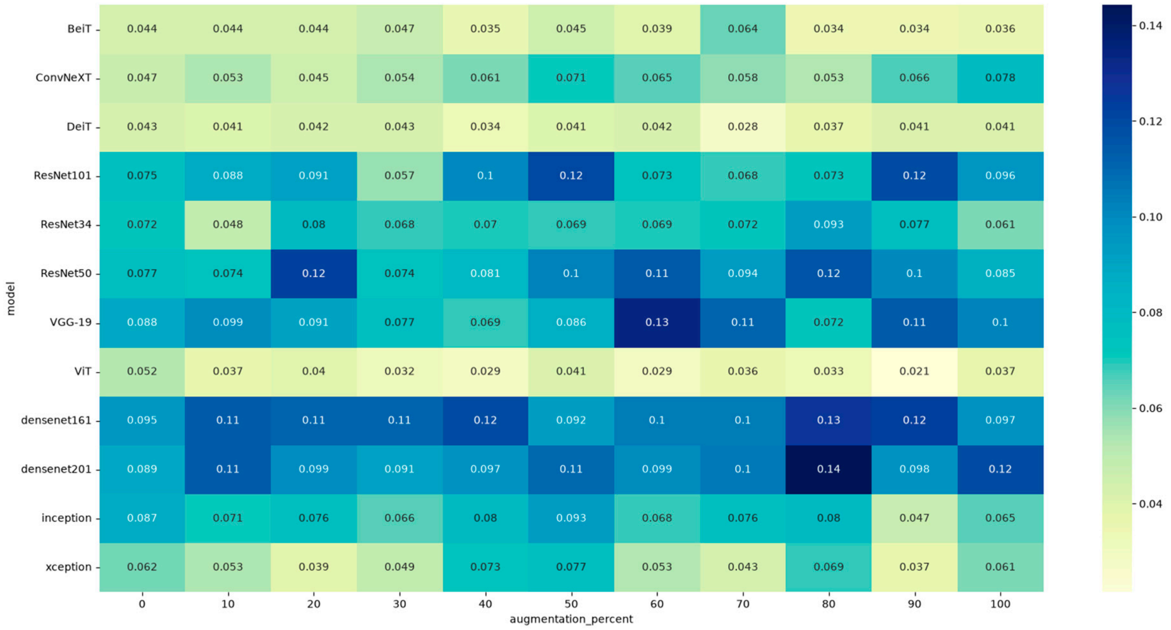

Figure S15. Legacy class panel: main C5 precision / replication C3 label unresolved

(a) Main dataset

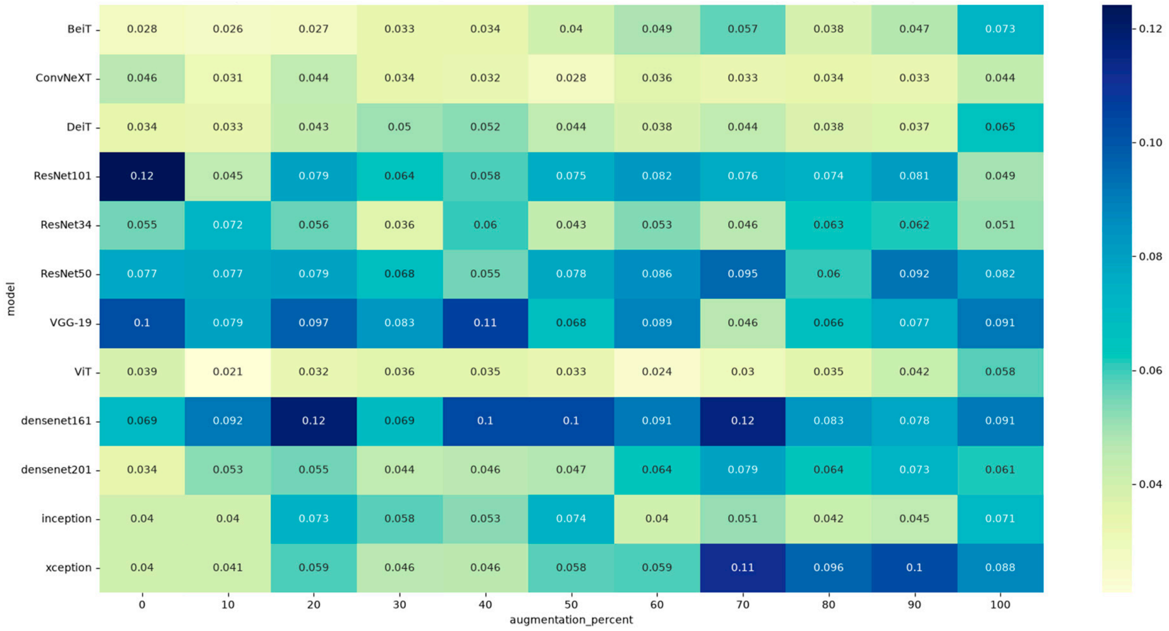

(b) Replication dataset

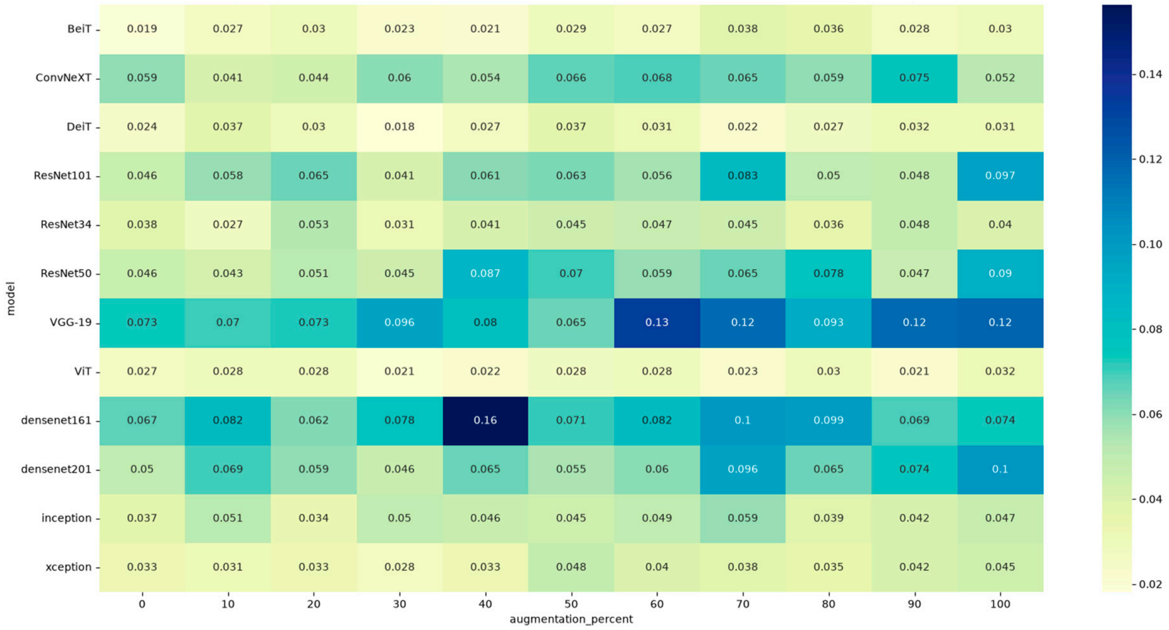

Figure S16. Originally underrepresented class F1: C5 / C3

https://doi.org/10.3390/jimaging12100502

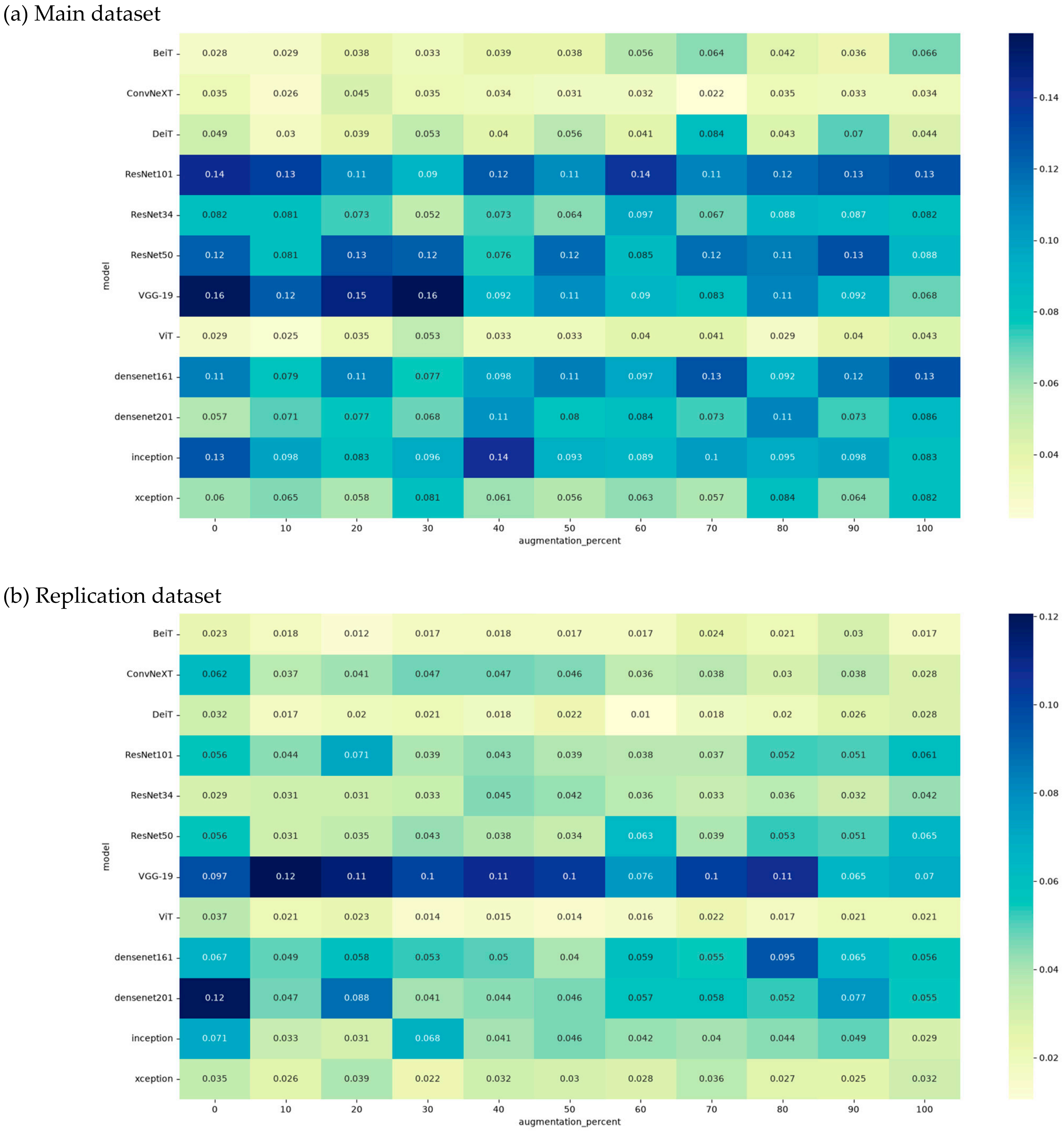

Figure S17. Originally underrepresented class recall: C6 / C7

(a) Main dataset

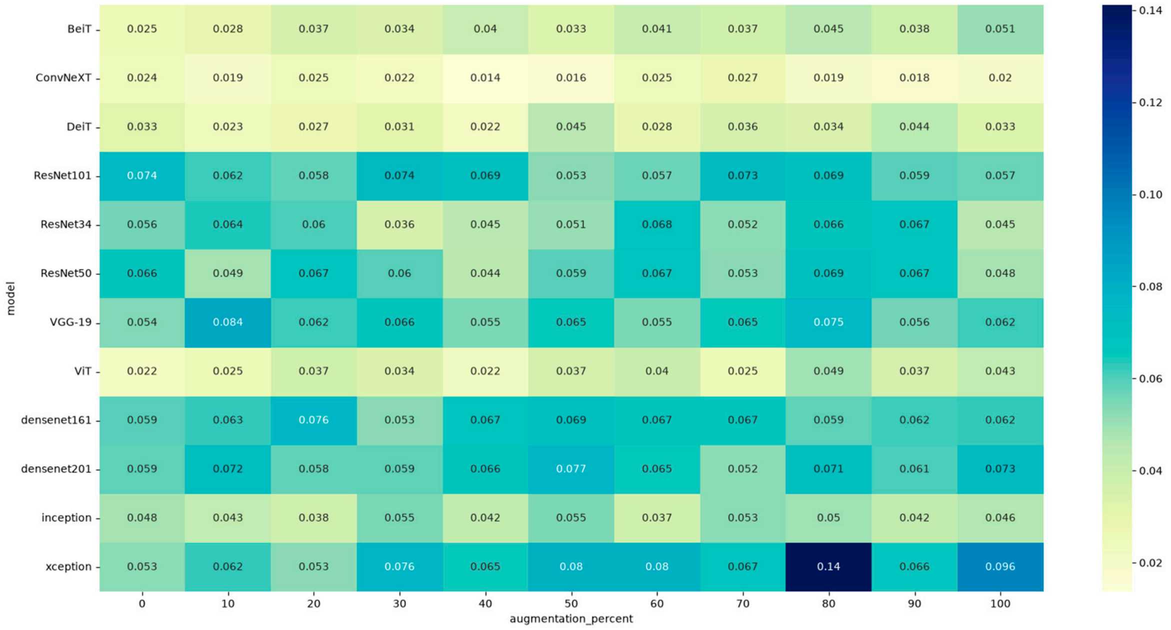

(b) Replication dataset

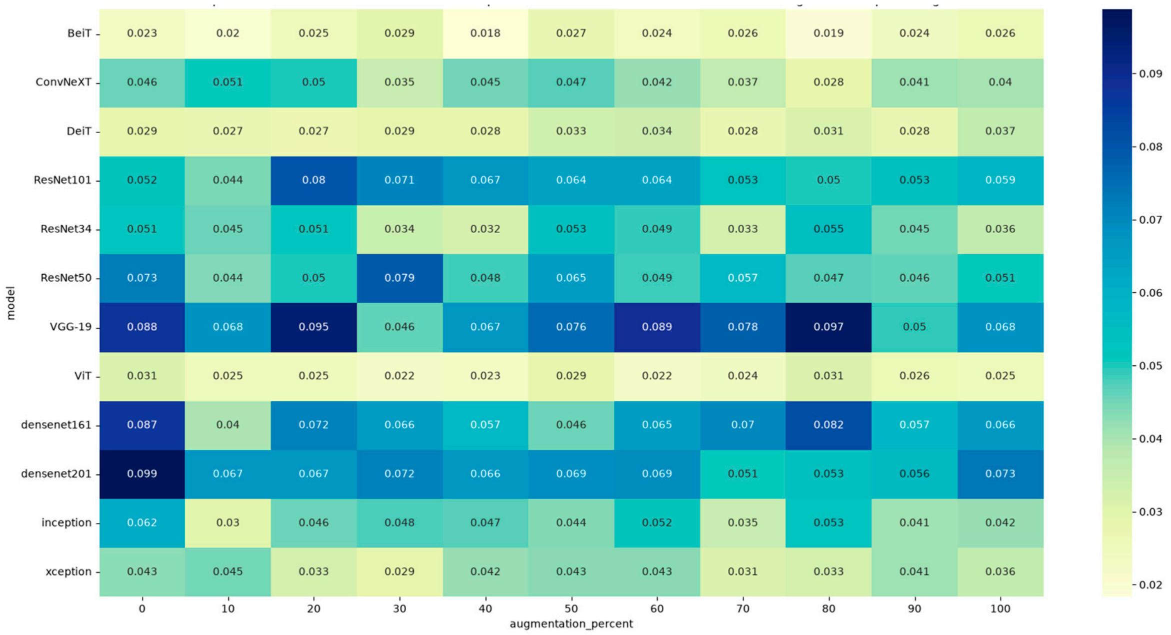

Figure S18. Originally underrepresented class precision: C6 / C7

(a) Main dataset

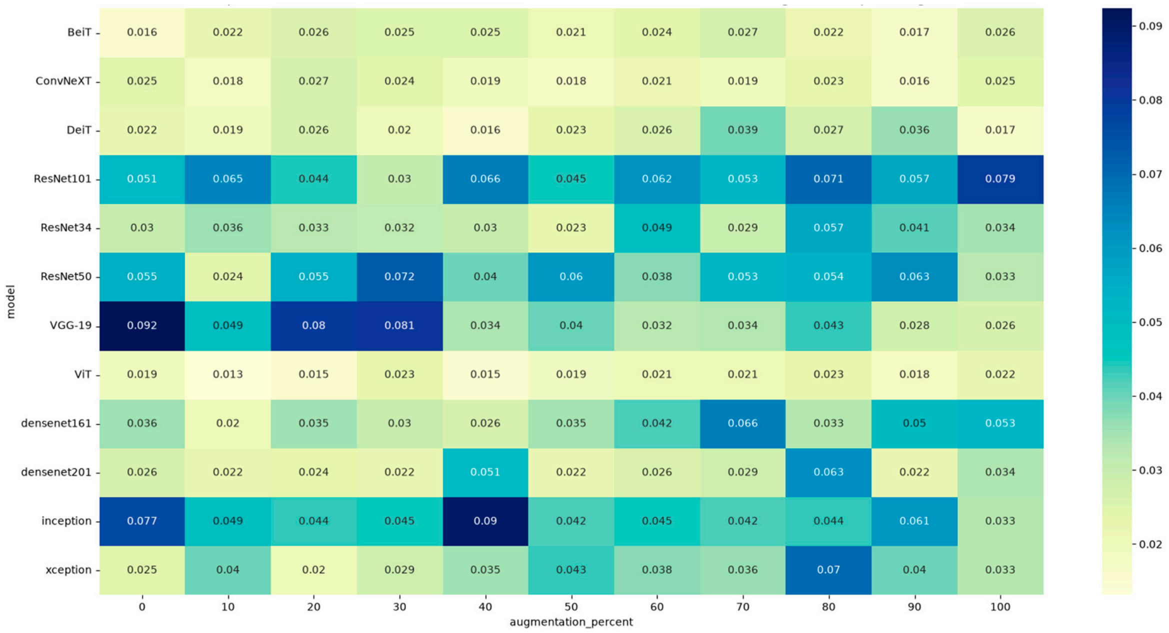

(b) Replication dataset

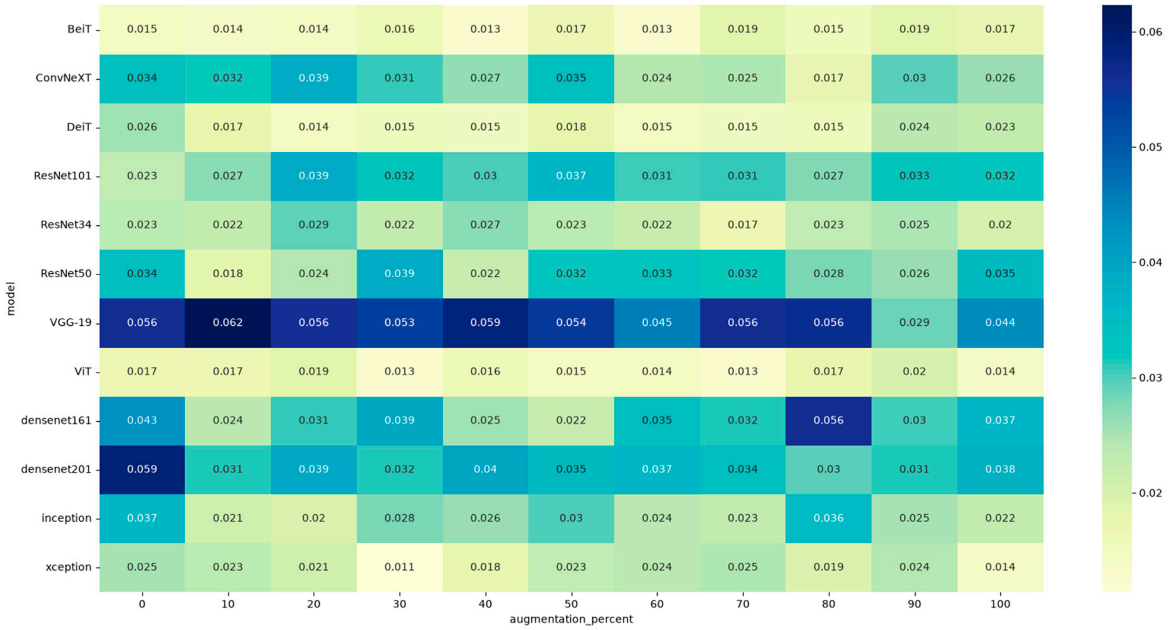

Figure S19. Originally underrepresented class F1: C6 / C7

(a) Main dataset

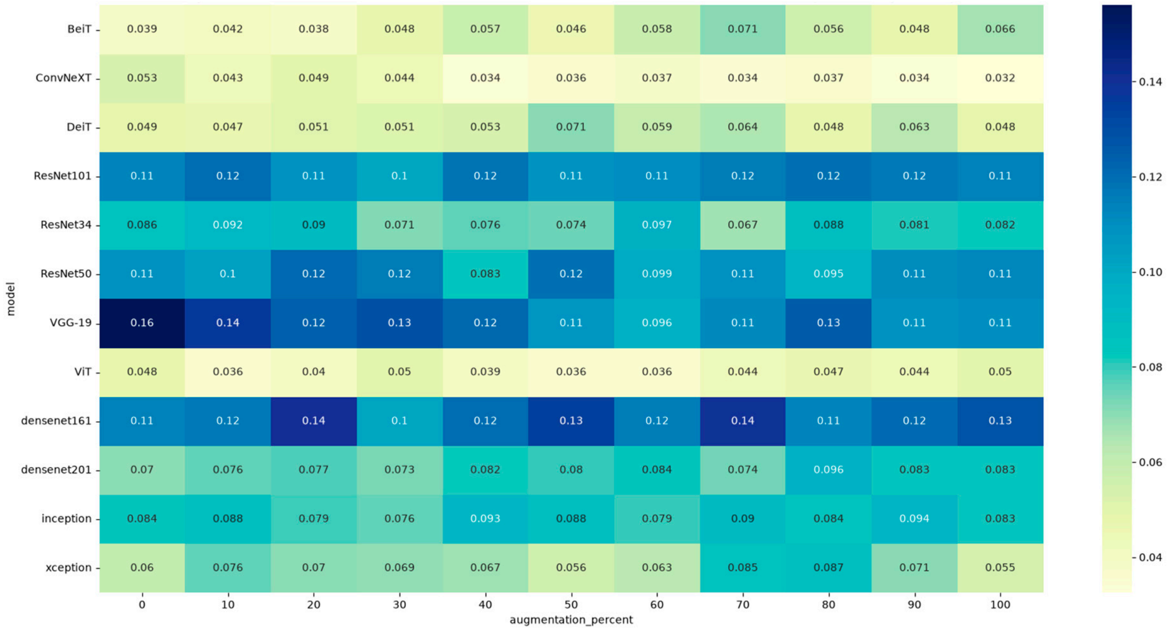

(b) Replication dataset

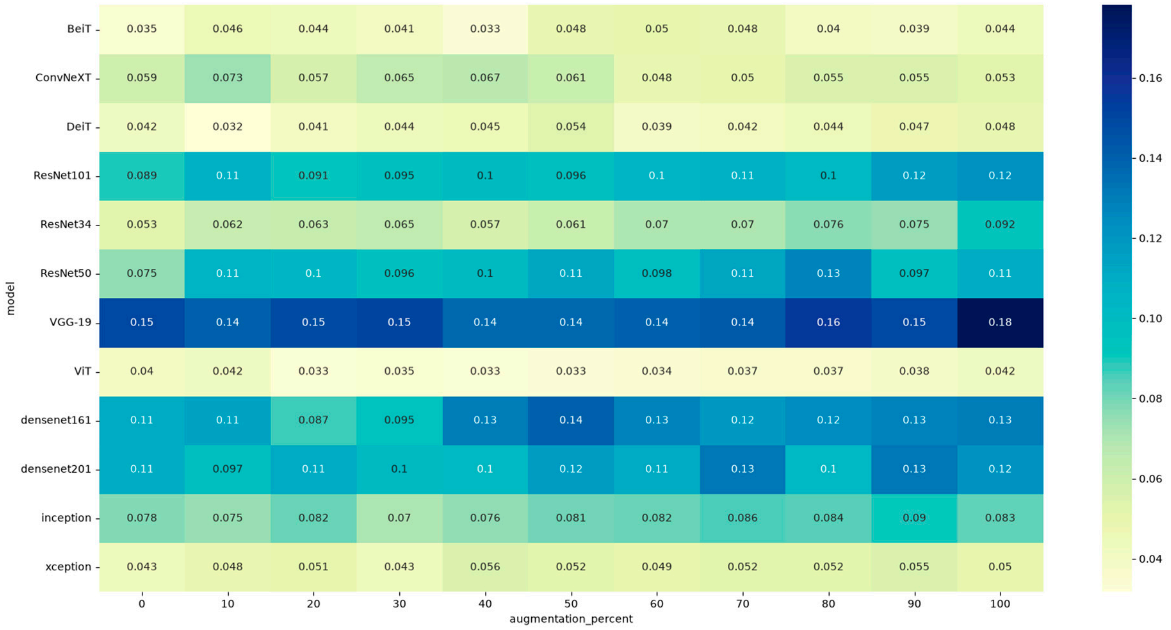

Figure S20. Median class-wise recall dispersion

https://doi.org/10.3390/jimaging12100502

(a) Main dataset

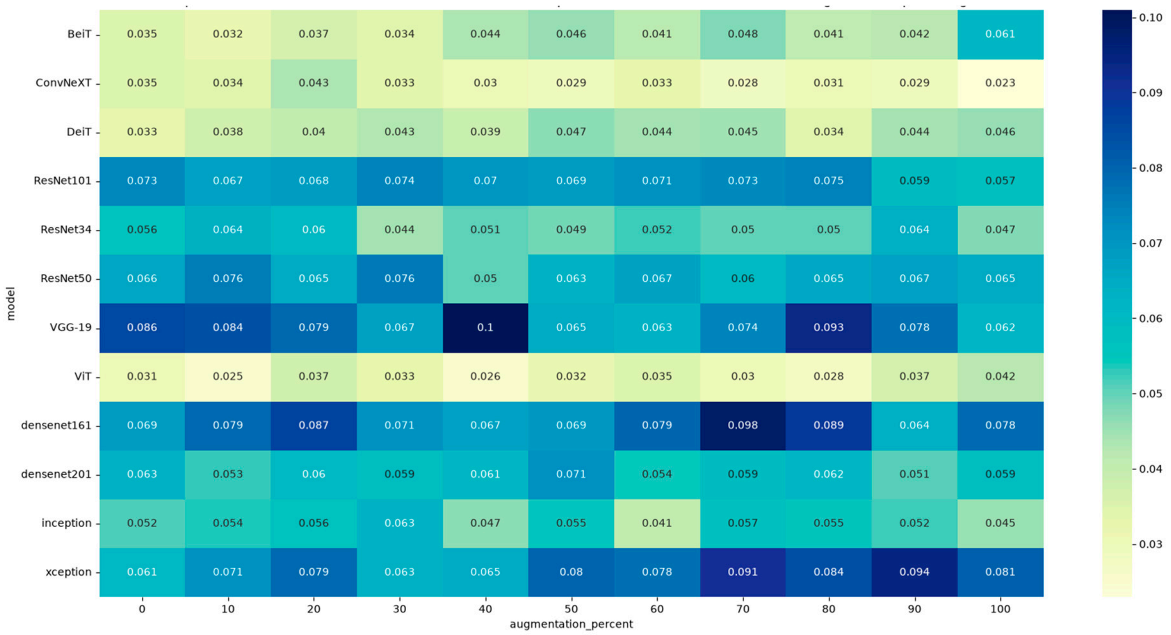

(b) Replication dataset

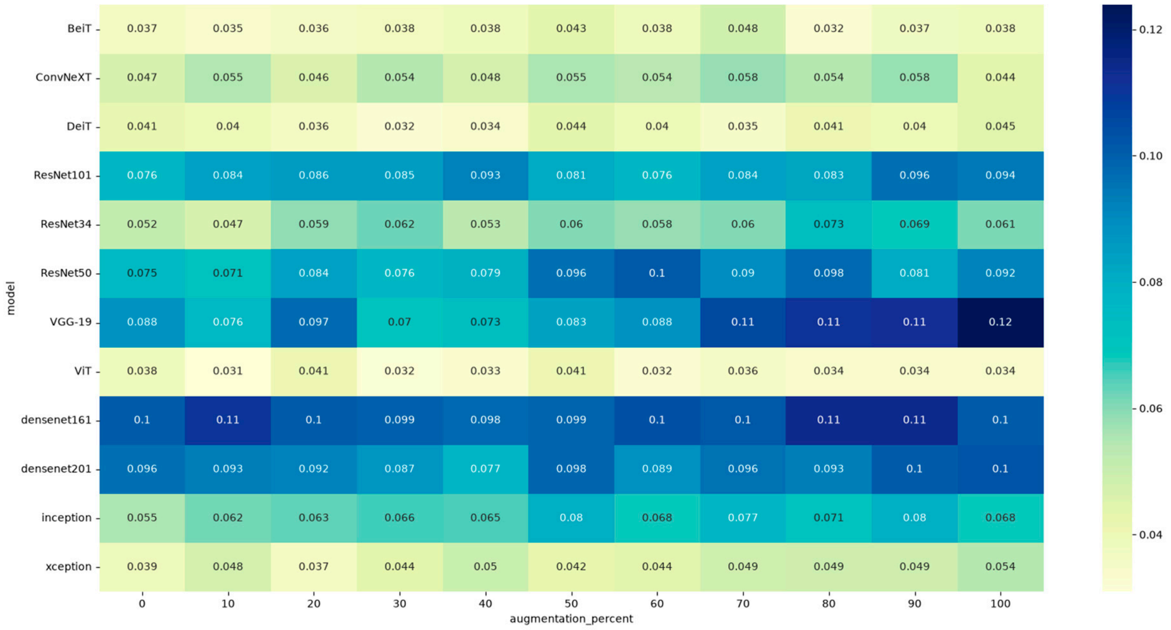

Figure S21. Median class-wise precision dispersion

(a) Main dataset

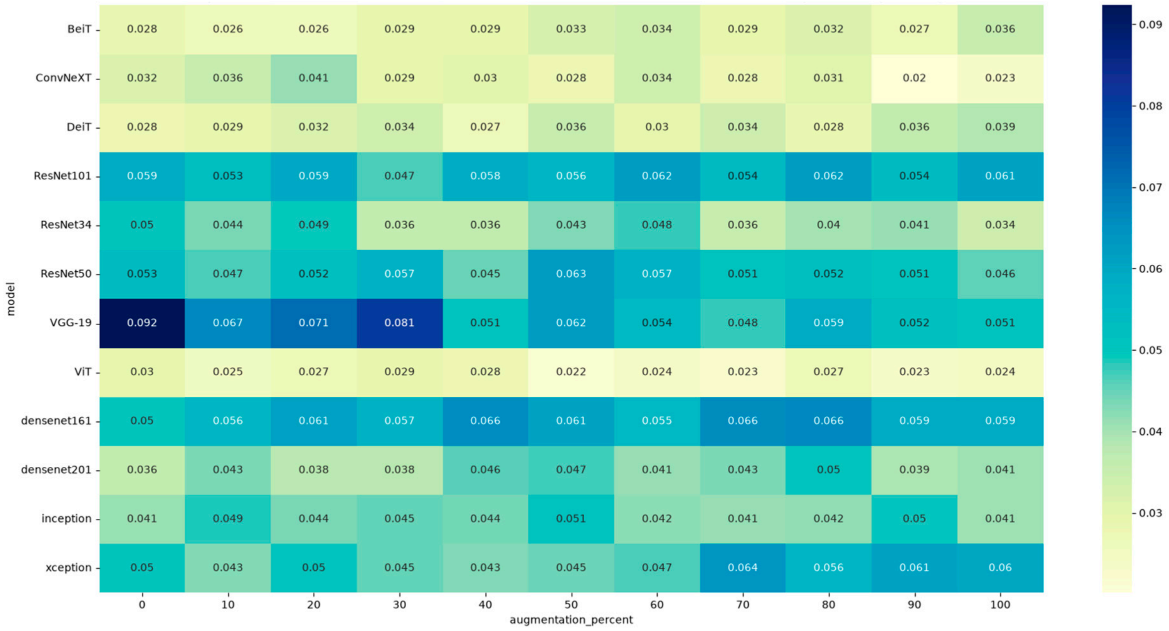

(b) Replication dataset

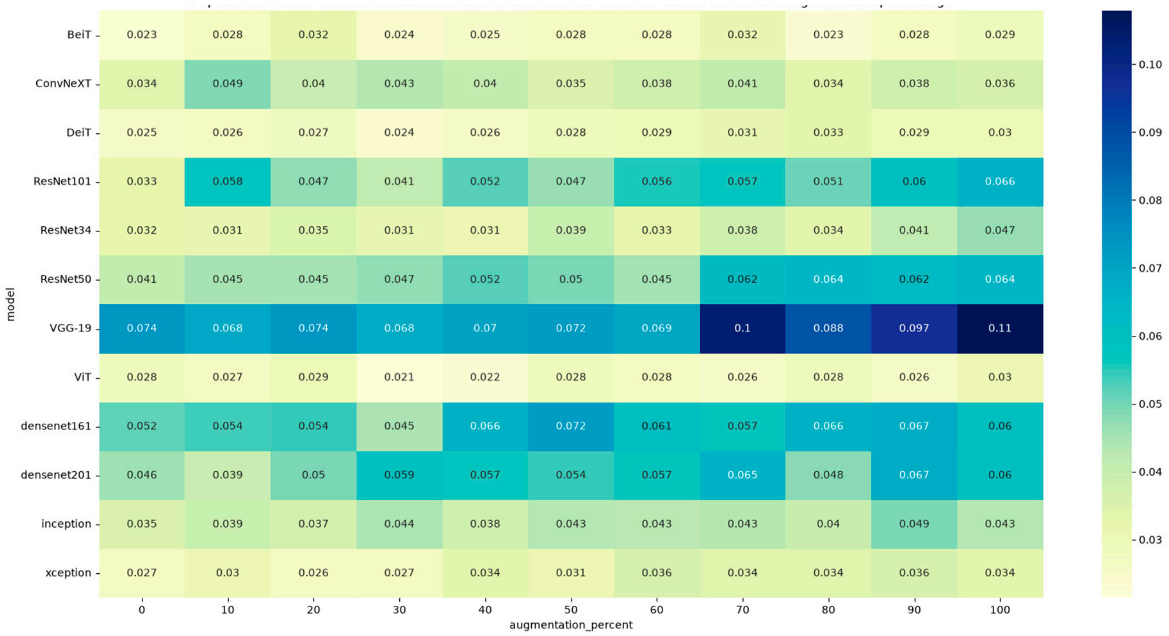

Figure S22. Median class-wise F1 dispersion

https://doi.org/10.3390/jimaging12100502

S3: 11 Matched-Level Pairs of Accuracy Box Plots.

Original box plots across the 20 runs.

(a) Main dataset

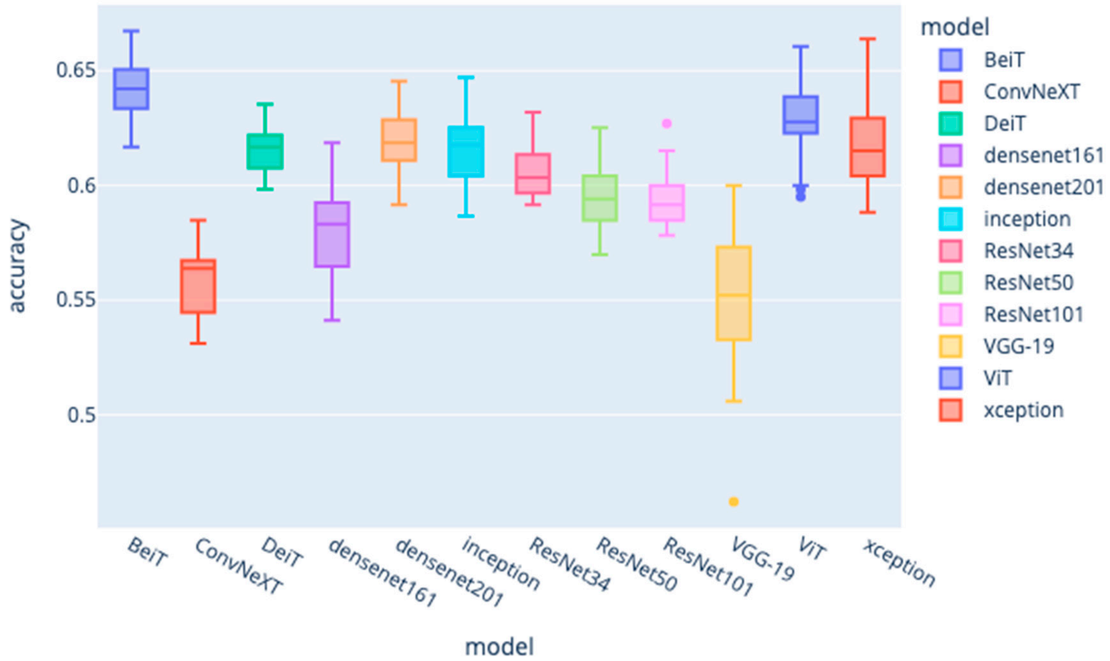

(b) Replication dataset

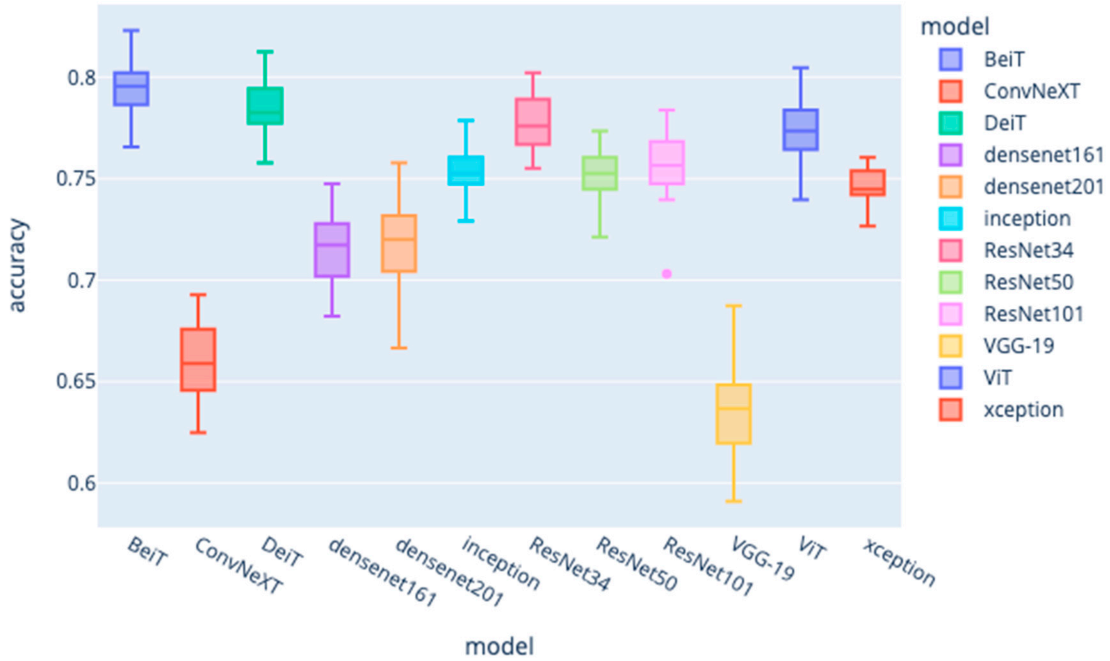

Figure S23. Accuracy distributions at r = 0%

(a) Main dataset

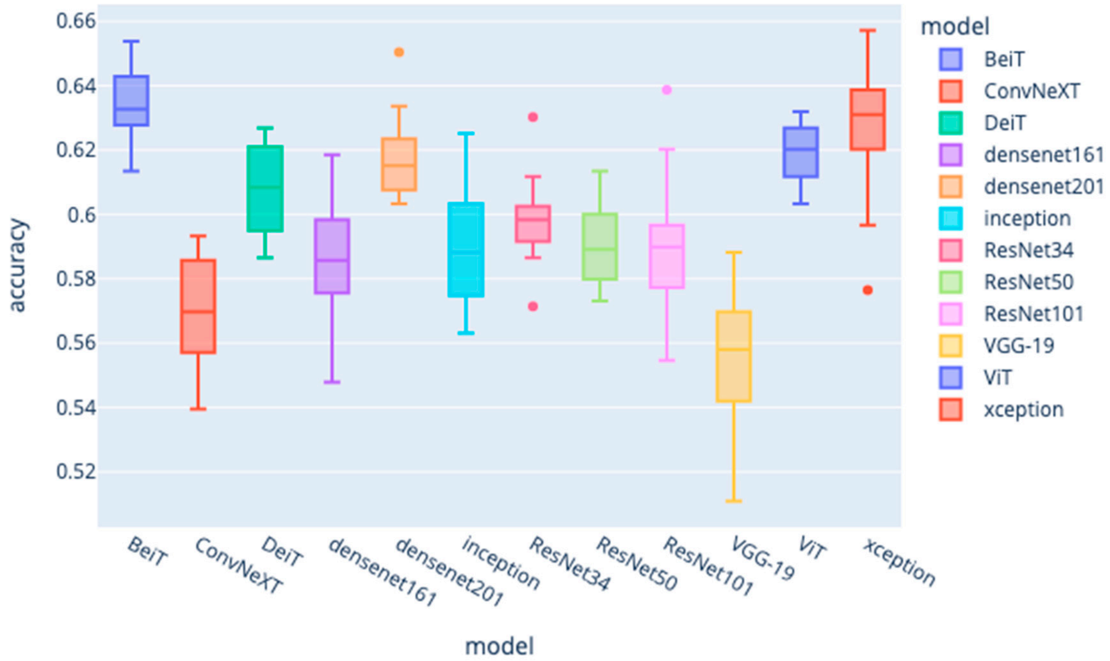

(b) Replication dataset

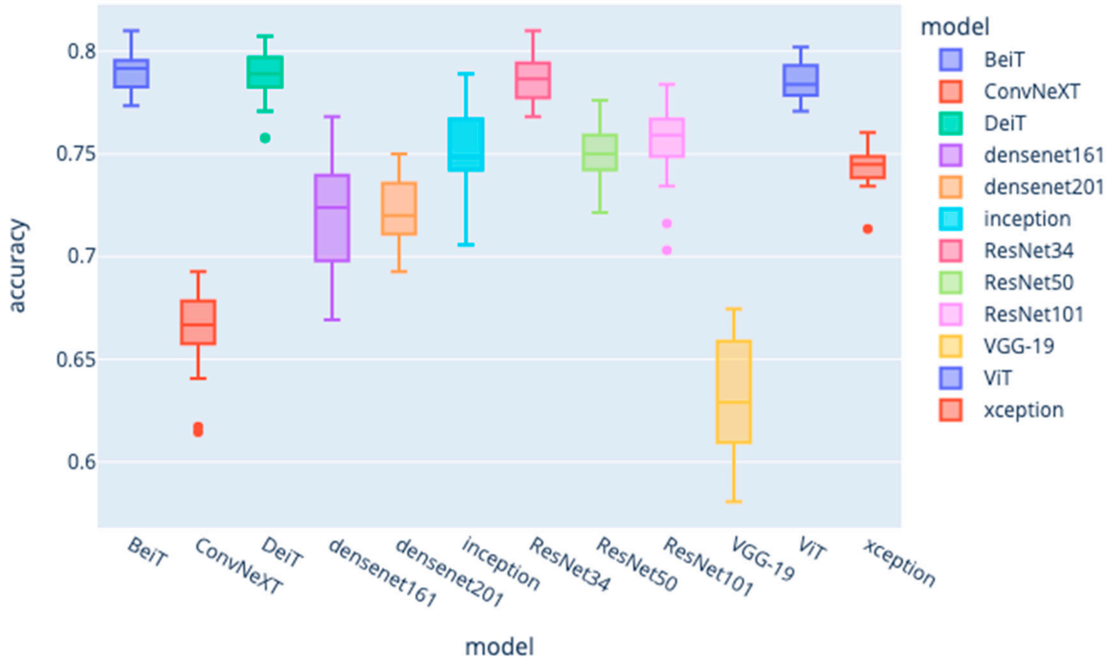

Figure S24. Accuracy distributions at r = 10%

https://doi.org/10.3390/jimaging12100502

(a) Main dataset

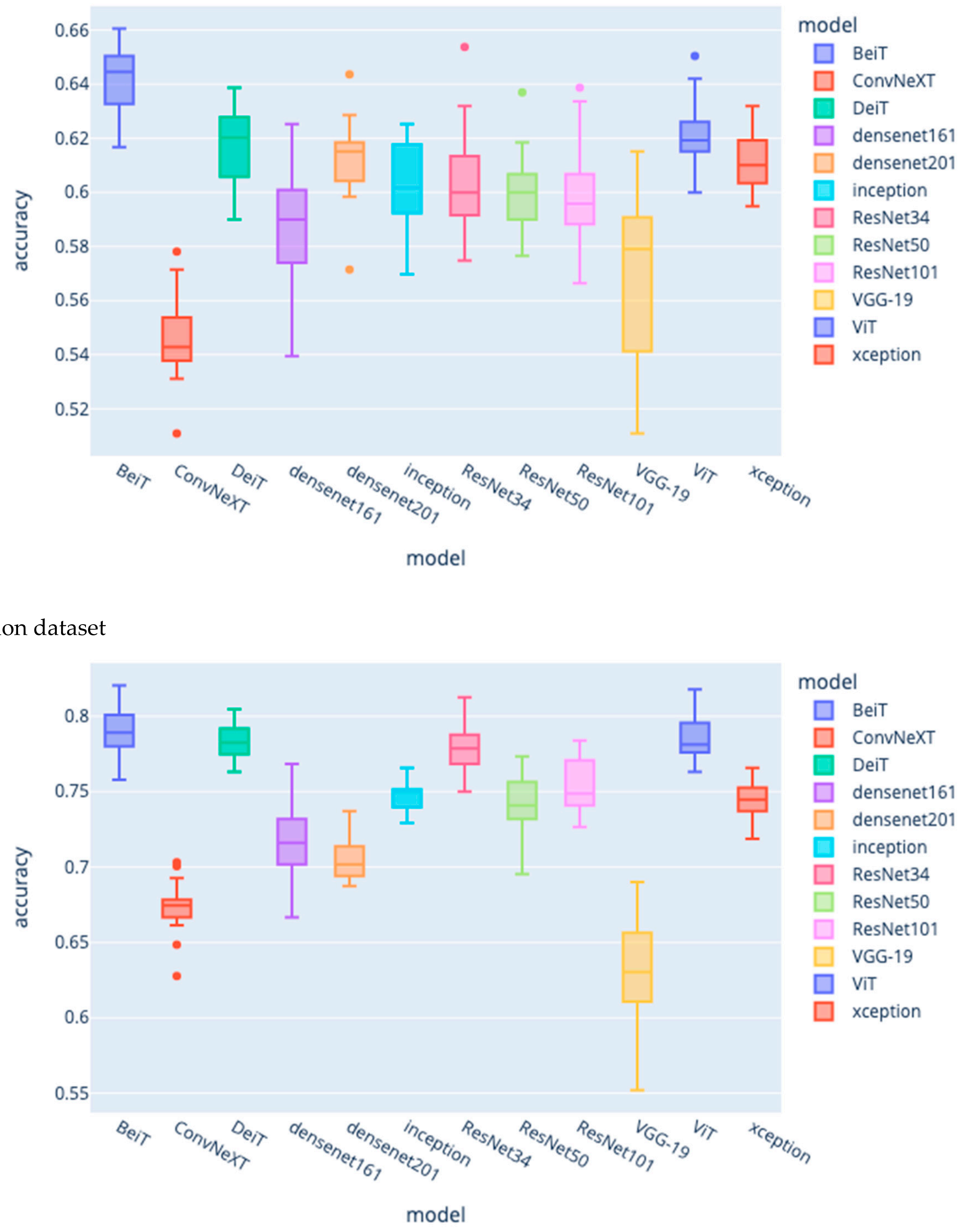

(b) Replication dataset

Figure S25. Accuracy distributions at r = 20%

(a) Main dataset

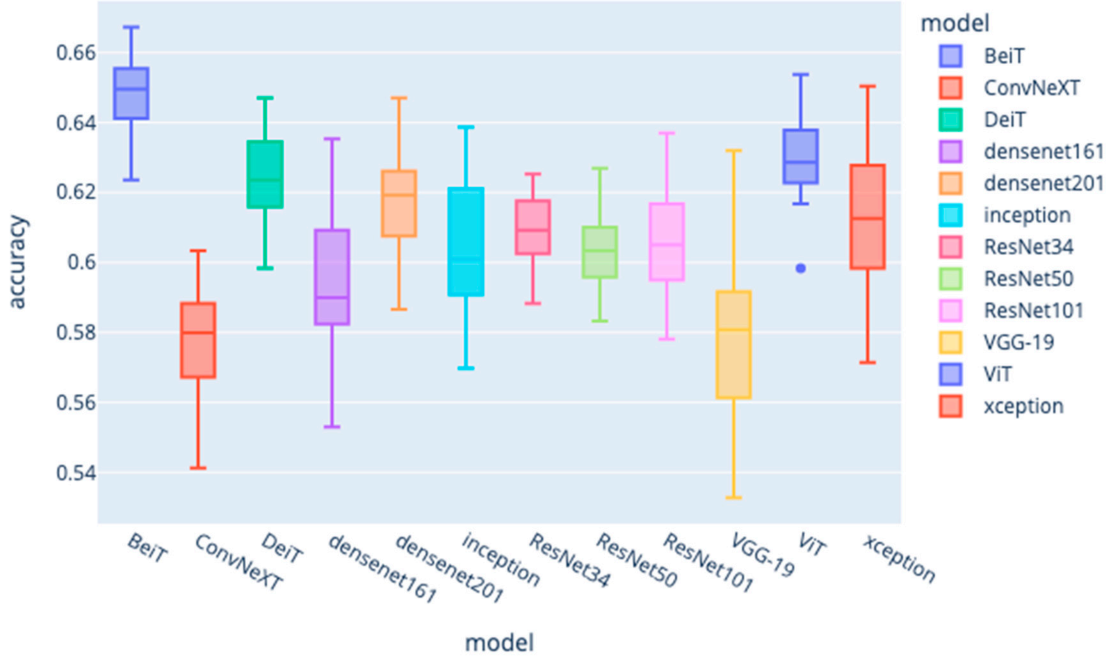

(b) Replication dataset

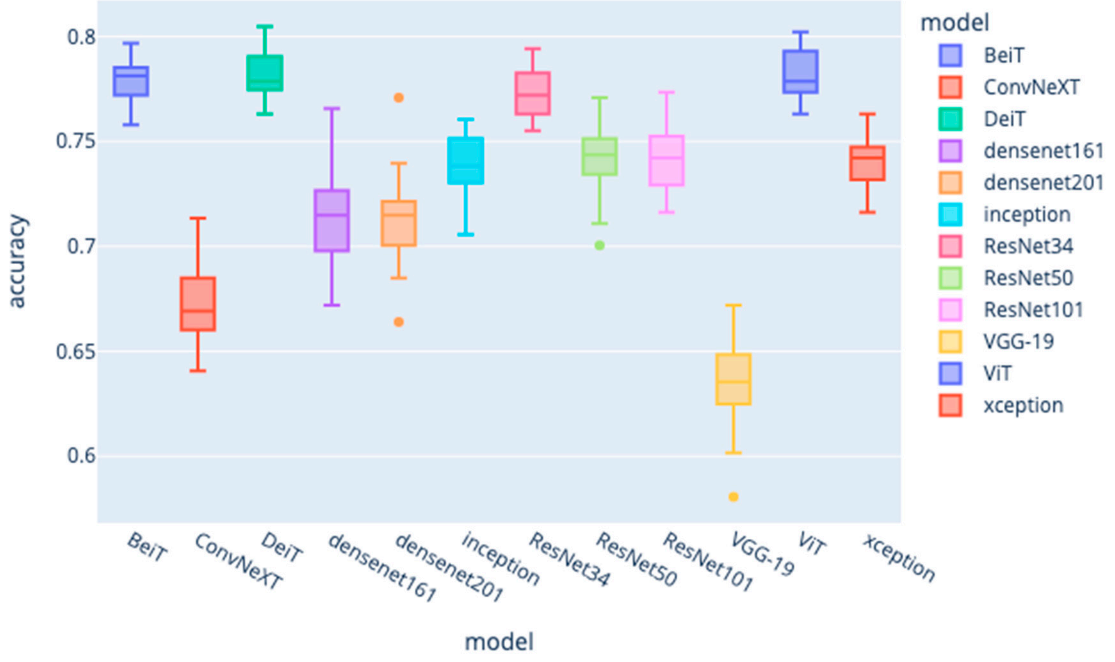

Figure S26. Accuracy distributions at r = 30%

(a) Main dataset

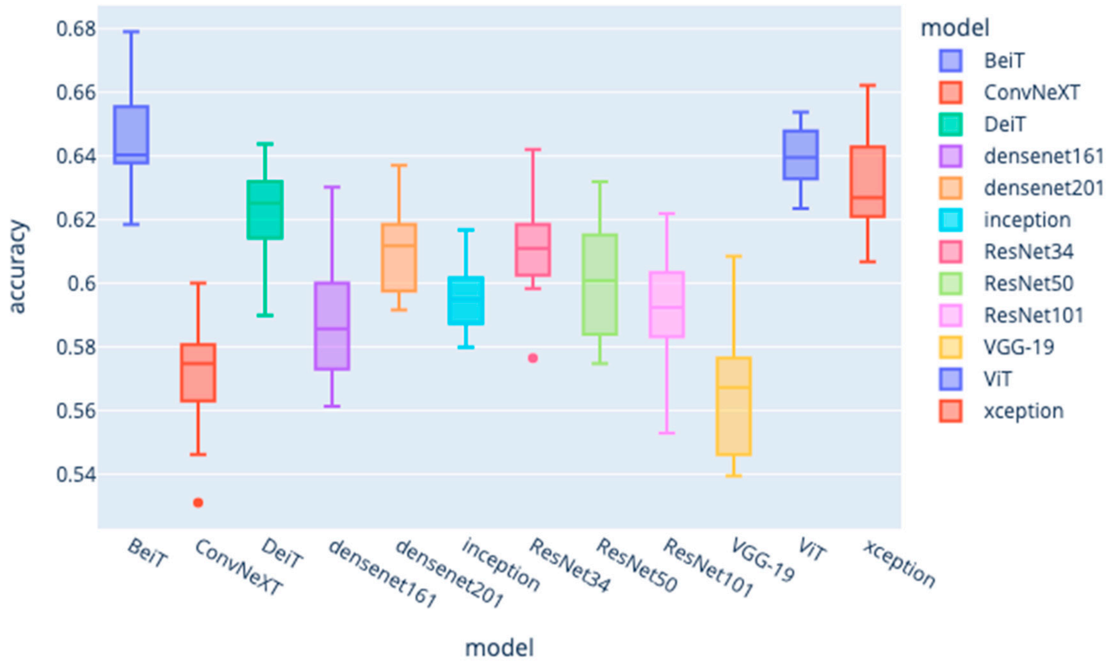

(b) Replication dataset

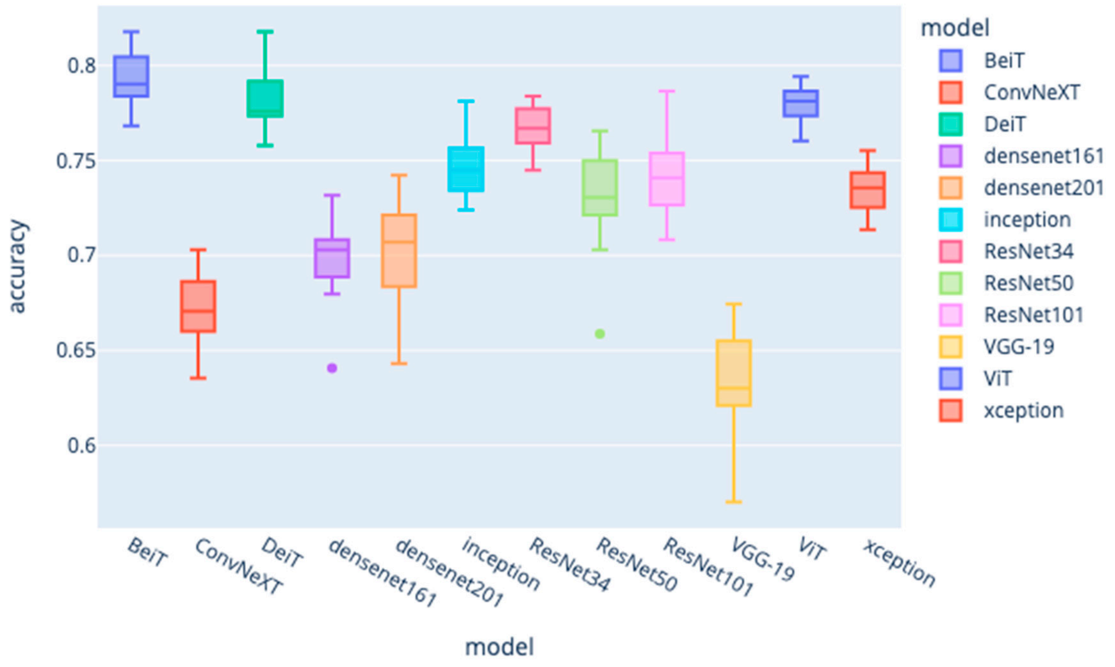

Figure S27. Accuracy distributions at r = 40%

(a) Main dataset

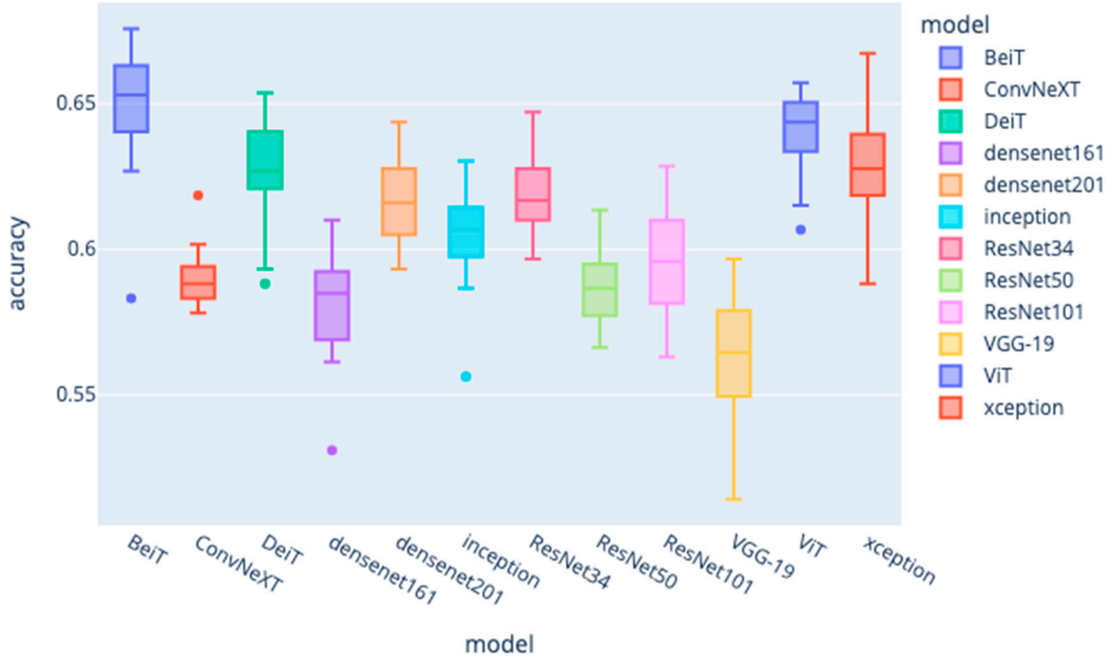

(b) Replication dataset

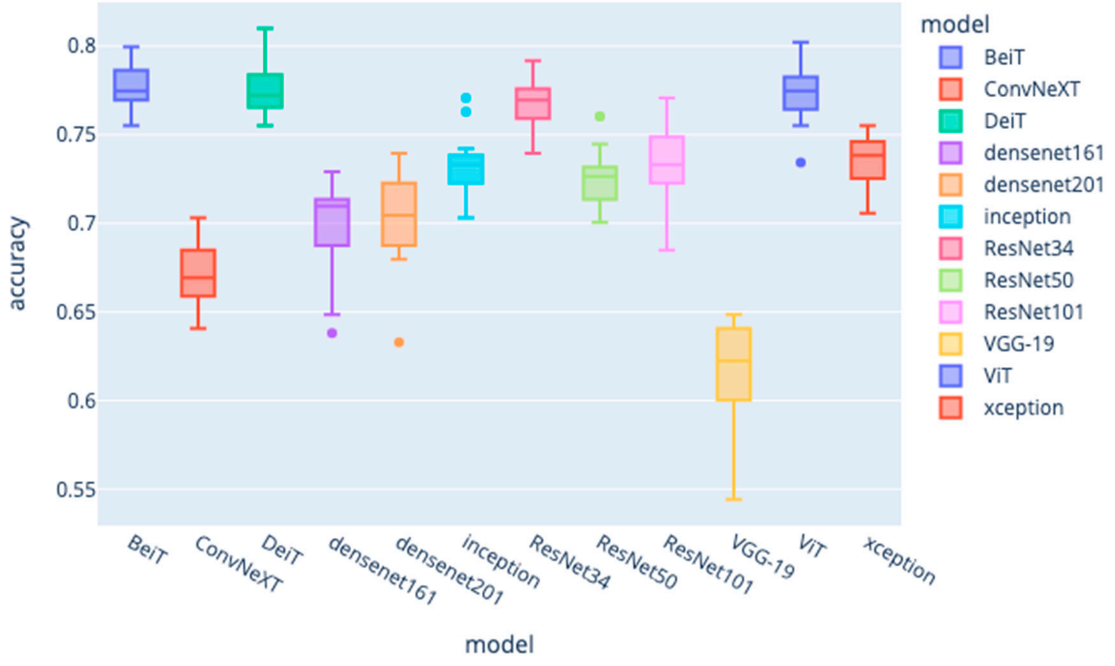

Figure S28. Accuracy distributions at r = 50%

(a) Main dataset

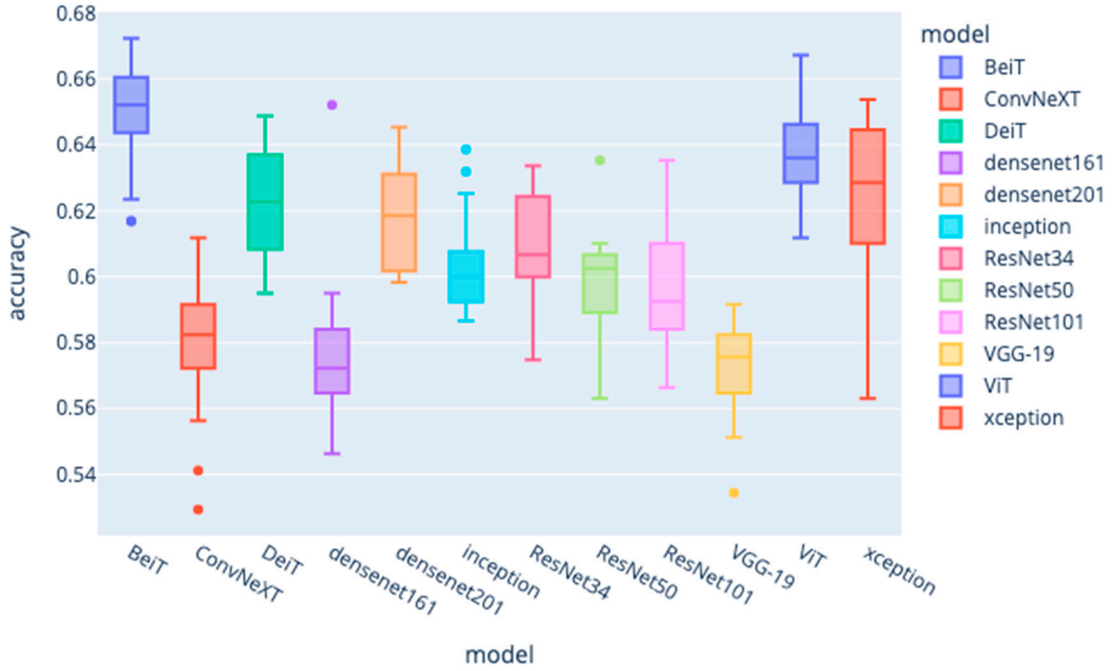

(b) Replication dataset

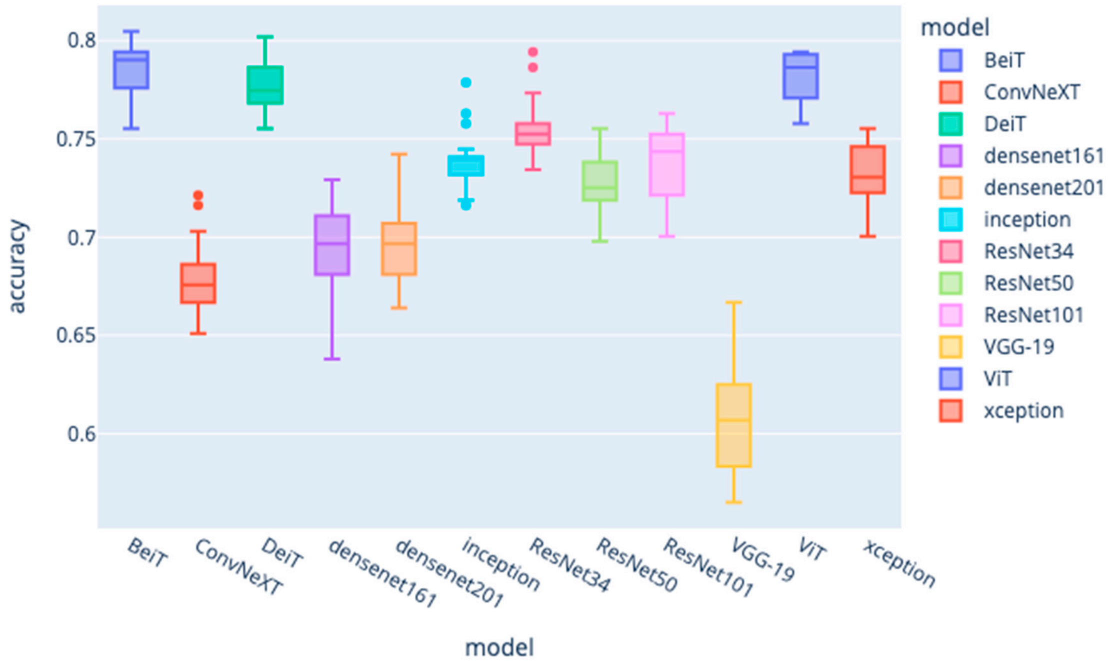

Figure S29. Accuracy distributions at r = 60%

https://doi.org/10.3390/jimaging12100502

(a) Main dataset

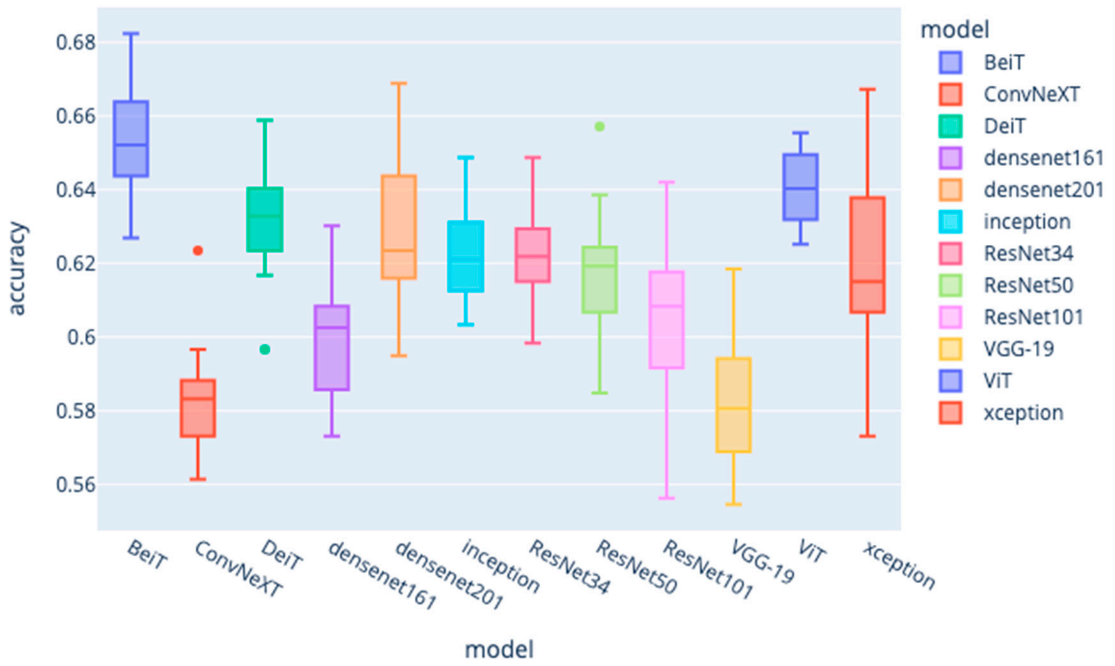

(b) Replication dataset

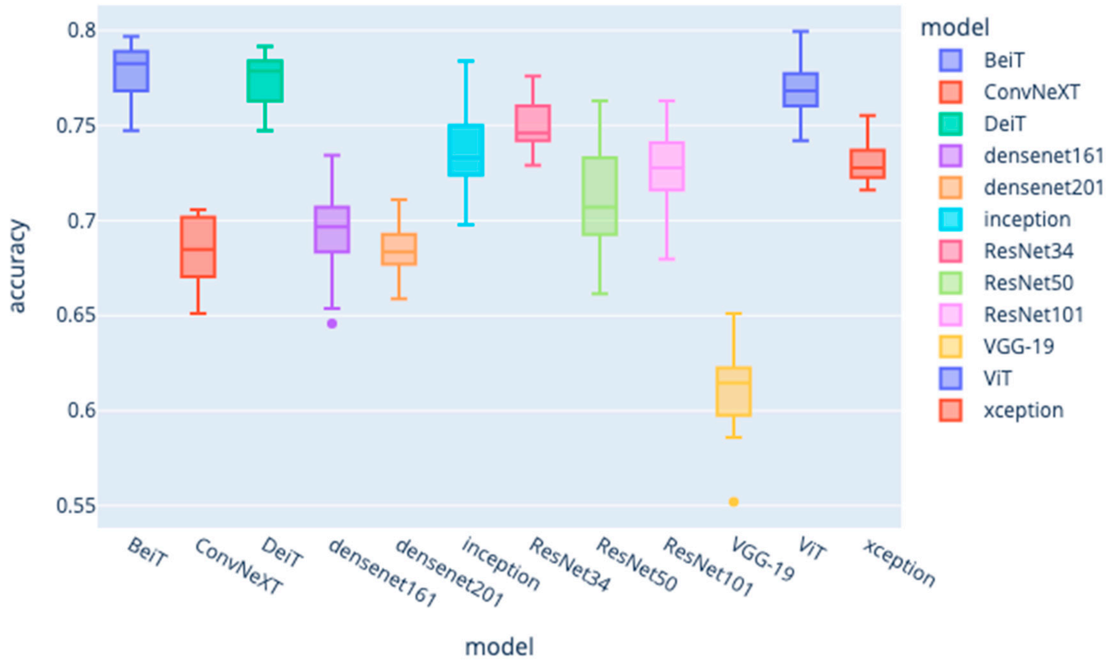

Figure S30. Accuracy distributions at r = 70%

(a) Main dataset

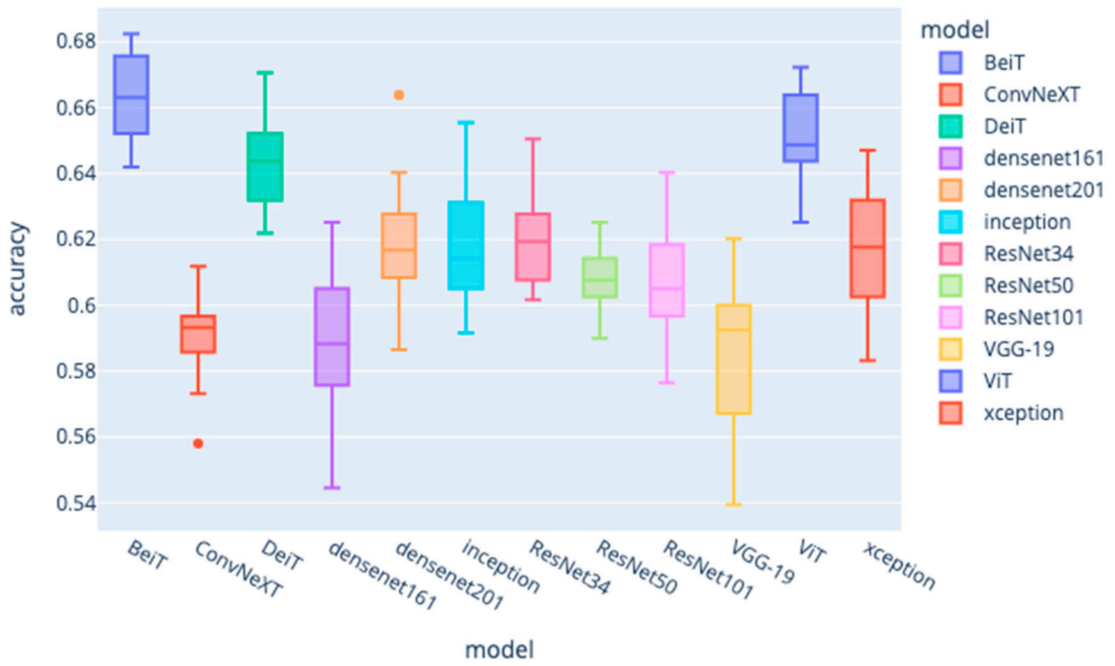

(b) Replication dataset

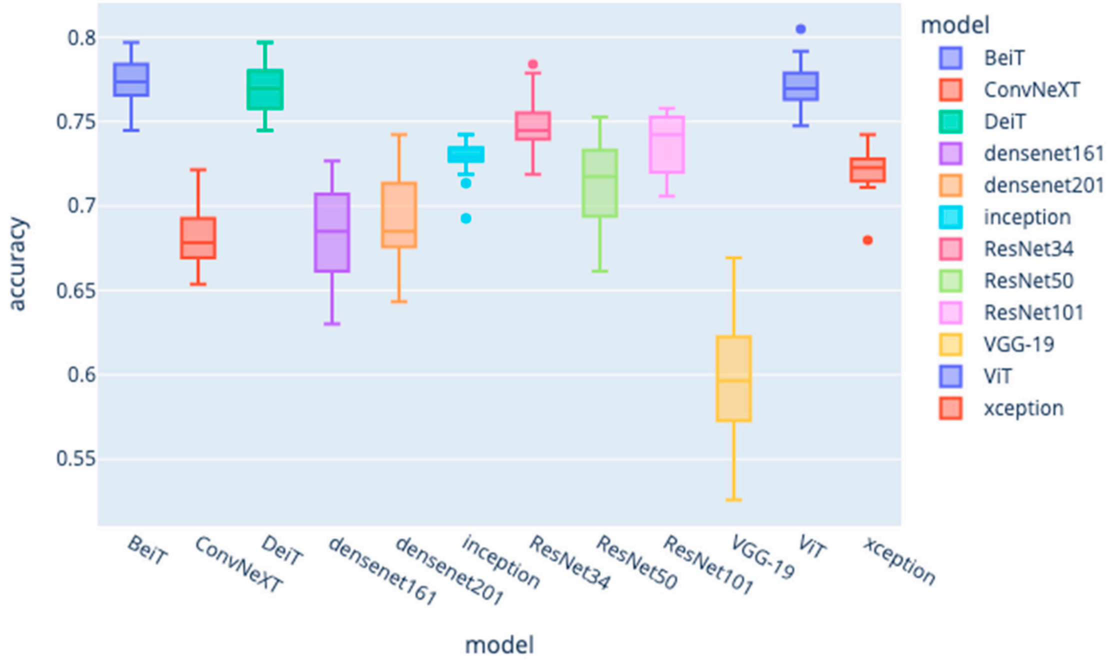

Figure S31. Accuracy distributions at r = 80%

(a) Main dataset

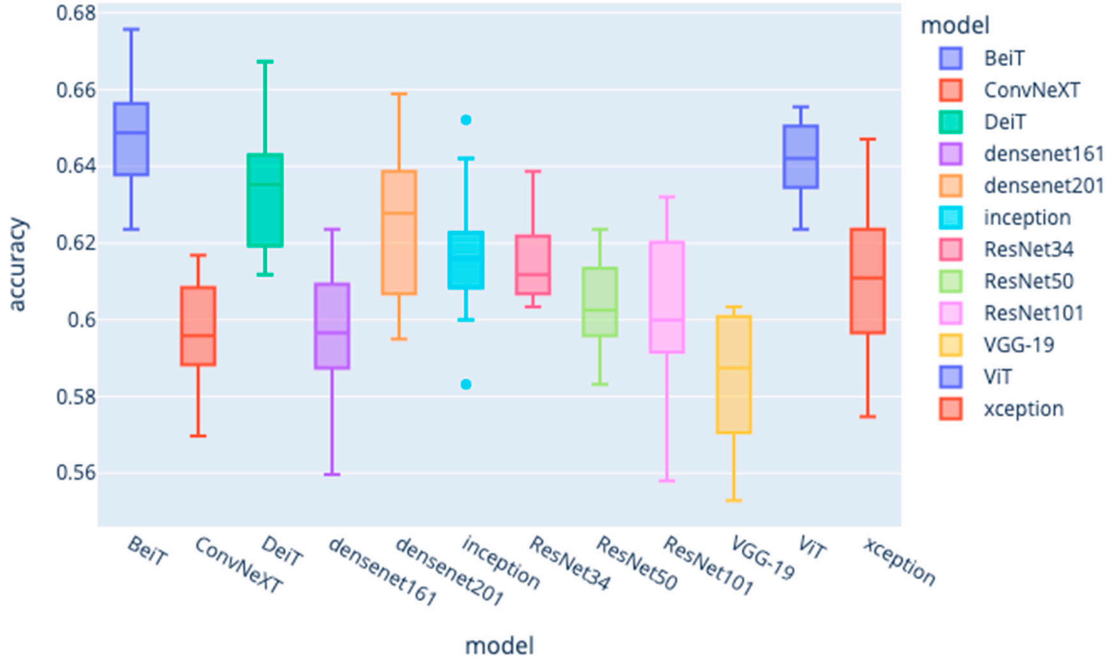

(b) Replication dataset

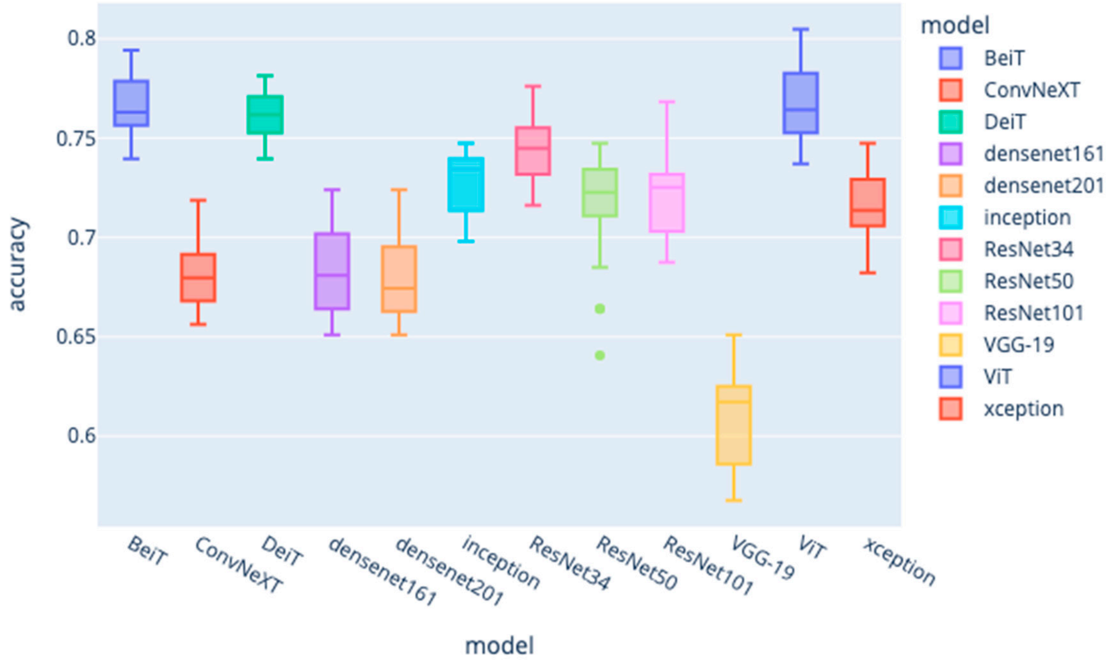

Figure S32. Accuracy distributions at r = 90%

https://doi.org/10.3390/jimaging12100502

(a) Main dataset

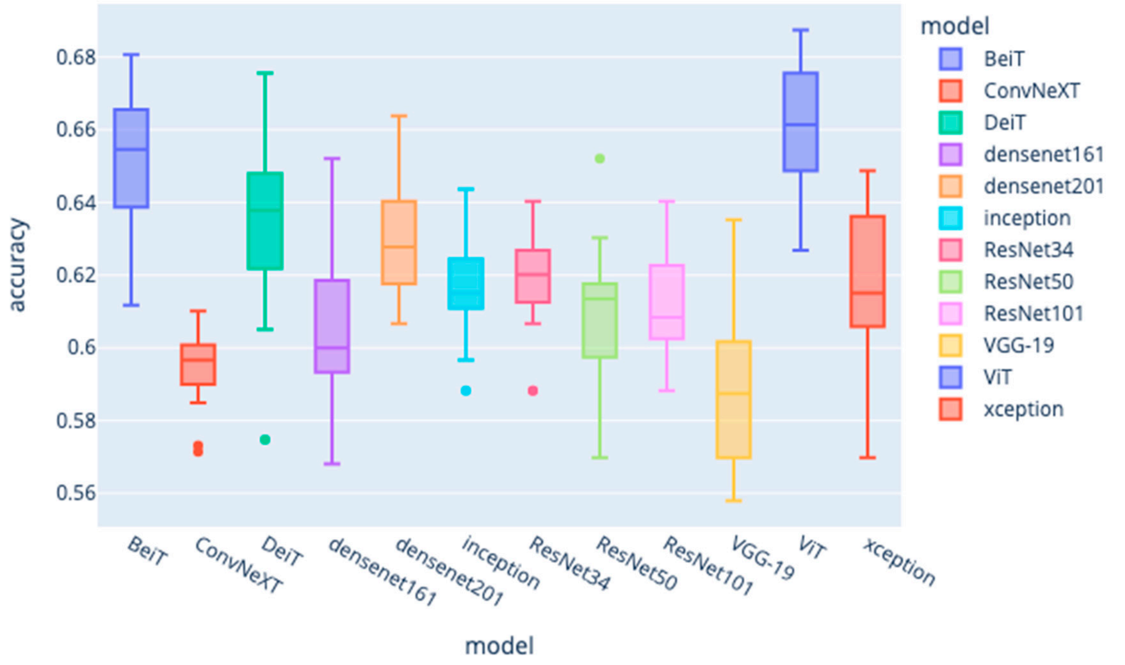

(b) Replication dataset

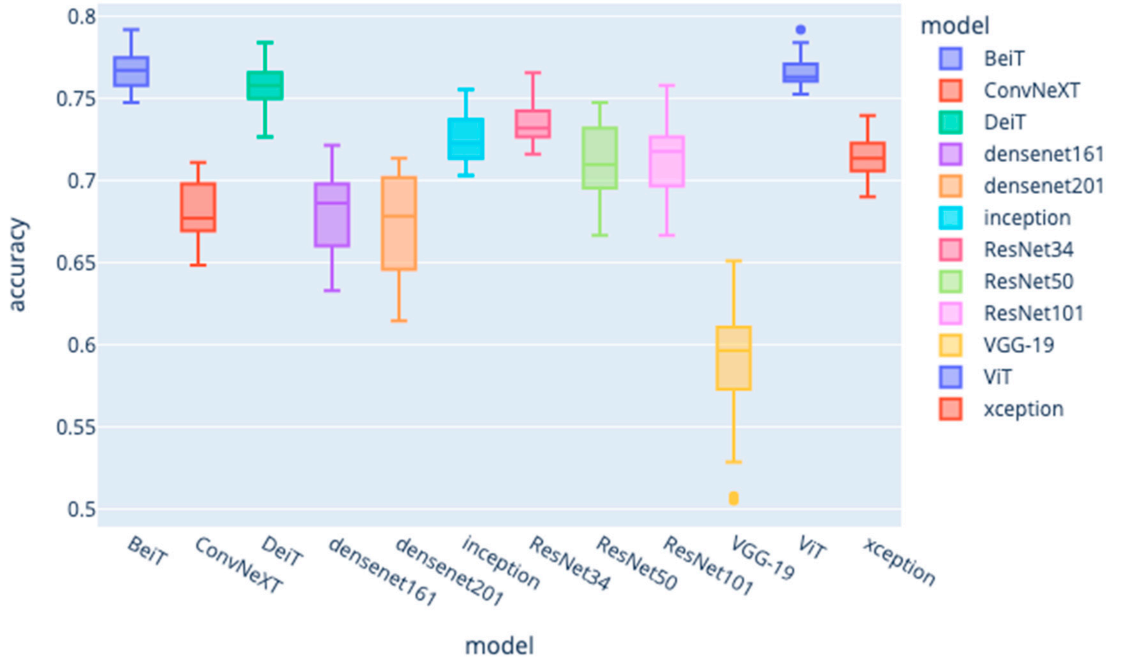

Figure S33. Accuracy distributions at r = 100%

S4. 2 Architecture-Matched Pairs of Illustrative Box Plots.

Original architecture-specific box plots, moved out of the main text. These are views of the same experimental runs, not additional experiments.

(a) Main dataset

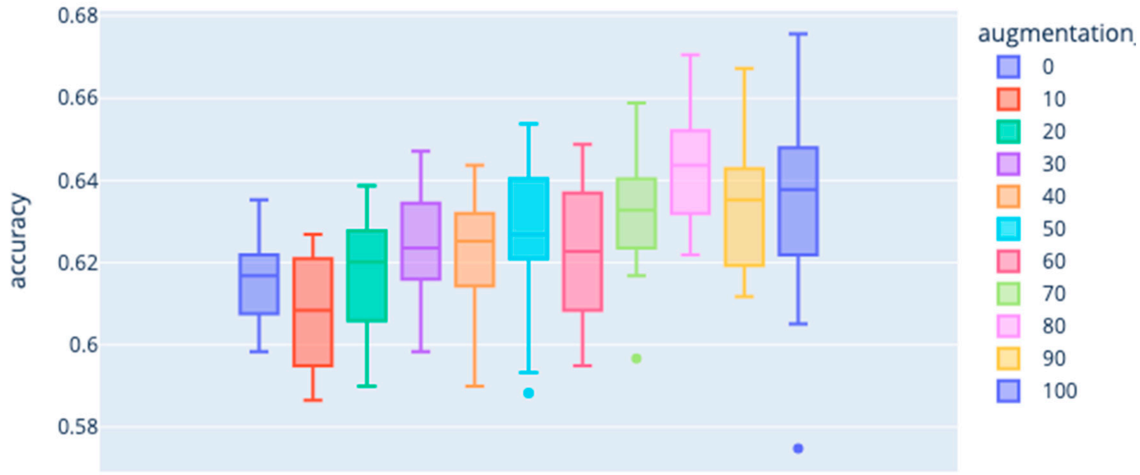

(b) Replication dataset

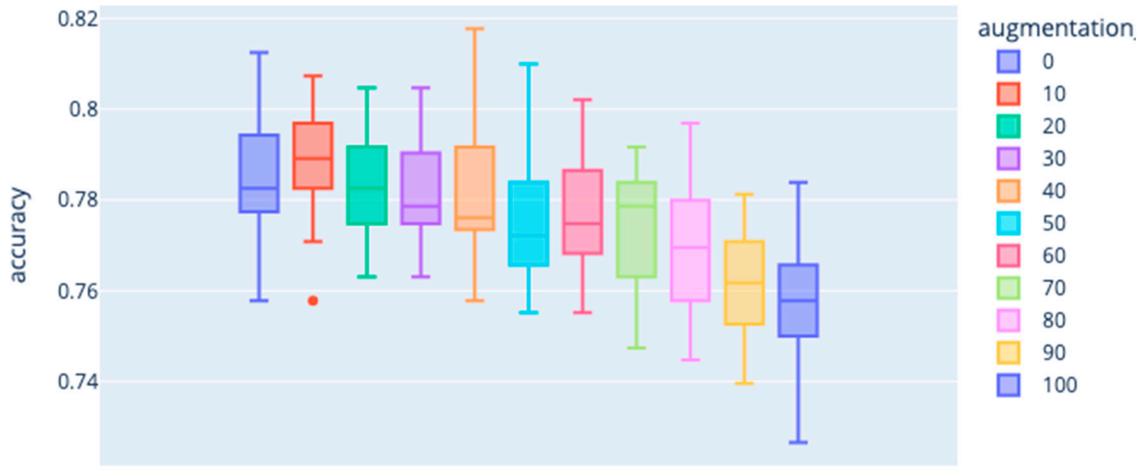

Figure S34. Illustrative DeiT distributions

https://doi.org/10.3390/jimaging12100502

(a) Main dataset

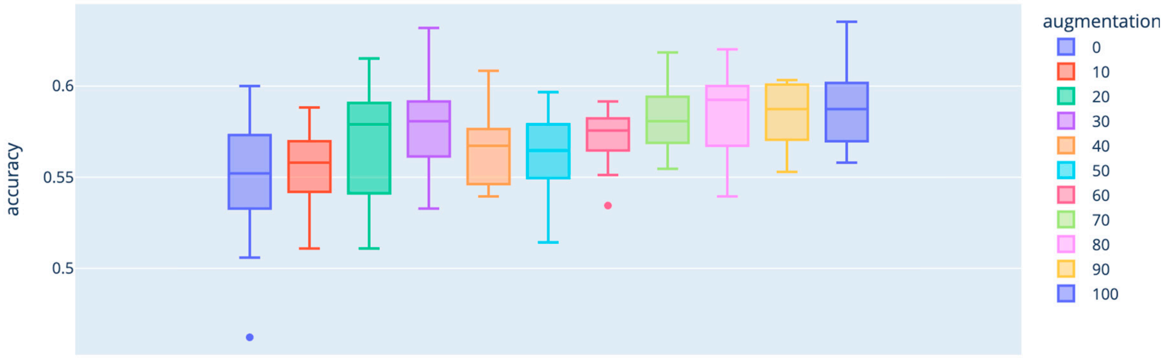

(b) Replication dataset

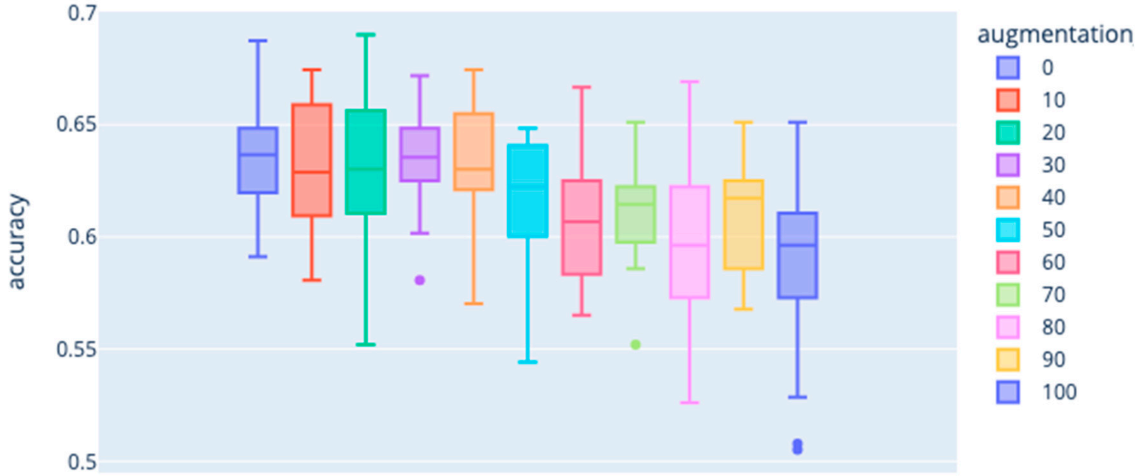

Figure S35. Illustrative VGG19 distributions

https://doi.org/10.3390/jimaging12100502
